# Design of Synthetic Surrogates for the Macrolactone Linker Motif in Coibamide A

**DOI:** 10.1021/acsmedchemlett.3c00232

**Published:** 2023-09-19

**Authors:** Rikito Suzuki, Daphne R. Mattos, Takashi Kitamura, Rina Tsujioka, Kazuya Kobayashi, Shinsuke Inuki, Hiroaki Ohno, Jane E. Ishmael, Kerry L. McPhail, Shinya Oishi

**Affiliations:** † Graduate School of Pharmaceutical Sciences, Kyoto University, Sakyo-ku, Kyoto 606-8501, Japan; ‡ Laboratory of Medicinal Chemistry, Kyoto Pharmaceutical University, Yamashina-ku, Kyoto 607-8412, Japan; § Department of Pharmaceutical Sciences, College of Pharmacy, Oregon State University, Corvallis, Oregon 97331, United States

**Keywords:** coibamide A, macrocyclic peptide, peptidomimetics, Sec61, translocon

## Abstract

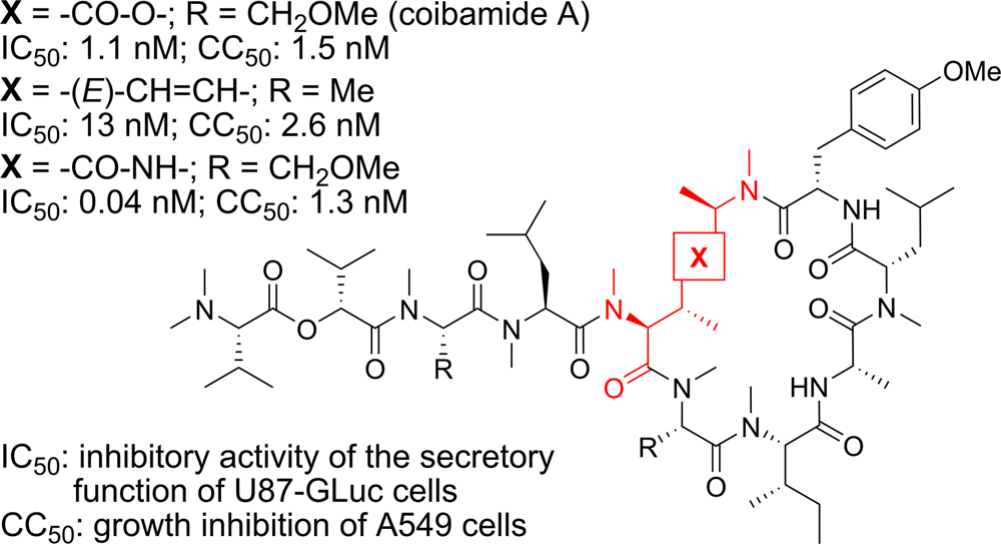

A marine cyanobacterial cyclic depsipeptide, coibamide A (CbA), inhibits the mammalian protein secretory pathway by blocking the Sec61 translocon, which is an emerging drug target for cancer and other chronic diseases. In our previous structure–activity relationship study of CbA, the macrolactone ester linker was replaced with alkyl/alkenyl surrogates to provide synthetically accessible macrocyclic scaffolds. To optimize the cellular bioactivity profile of CbA analogues, novel lysine mimetics having β- and ε-methyl groups have now been designed and synthesized by a stereoselective route. A significant increase in cytotoxicity was observed upon introduction of these two methyl groups, corresponding to the d-MeAla α-methyl and MeThr β-methyl of CbA. All synthetic products retained the ability to inhibit secretion of a model Sec61 substrate. Tandem evaluation of secretory function inhibition in living cells and cytotoxicity was an effective strategy to assess the impact of structural modifications to the linker for ring closure.

Coibamide A (CbA, **1a**) is a cyclic depsipeptide isolated from a Panamanian marine filamentous cyanobacterium.^1^ The highly *N*-methylated peptide, with one d-amino acid and a d-hydroxy acid (Figure 1A), exhibited potent antiproliferative activity against several human cancer cell lines.^1,2^ Using a synthetic CbA photoaffinity probe, we previously revealed that CbA binds to the α subunit of the Sec61 translocon (Sec61α) and prevents insertion of secreted and membrane proteins into the endoplasmic reticulum (ER) membrane or across the ER membrane into the ER luminal space.^3^ To date, several studies have examined the biological activity of CbA and the downstream target(s) that are impacted by the CbA-induced block of Sec61-dependent protein biosynthesis in the ER secretory pathway. First discovered in a screen for anticancer activity,^1^ CbA induces cell-cycle arrest, inhibits cancer cell migration and invasion *in vitro*, and retains antitumor properties in subcutaneous xenograft mouse models.^4^ This bioactivity profile may be mediated, at least in part, by inhibition of extracellular secreted proteins, such as vascular endothelial growth factor A (VEGF-A), as well as by decreases in the expression of integral membrane receptors, such as vascular endothelial growth factor receptor 2 (VEGFR2), and human epidermal growth factor receptor (HER) members, especially EGFR (ErbB-1) and HER3 (ErbB-3).^4,5^ CbA also induces macroautophagy via a mammalian target of rapamycin (mTOR)-independent mechanism.^6^ CbA-induced autophagy, following short-term exposure of cells, depends on the presence of autophagy-related protein 5 (ATG5), and eventually defects in the autophagosome–lysosomal fusion promote regulated cell death signaling.^7,8^

**Figure 1:**
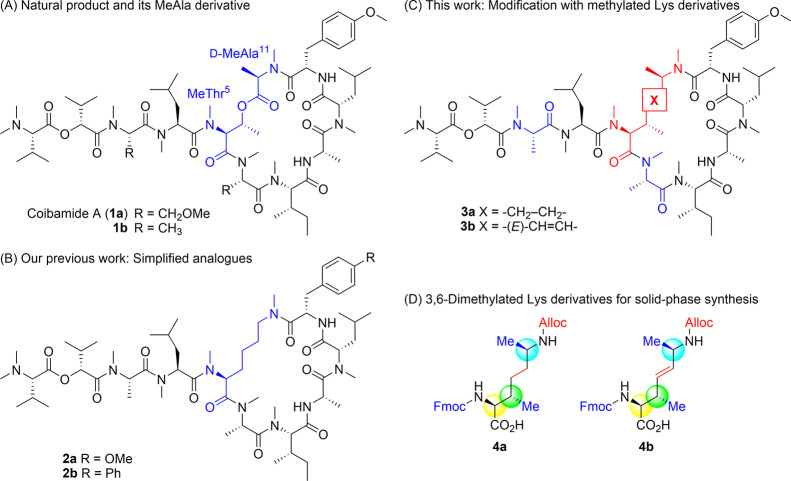
Molecular structures of coibamide A derivatives and design of 3,6-dimethylysine-containing derivatives.

There are a number of other natural products and synthetic molecules that selectively inhibit protein secretion via Sec61α inhibition, including apratoxin A,^9,10^ decatransin,^11^ eeyarestatin I,^12,13^ HUN-7293,^14−16^ ipomoeassin F,^17^ and mycolactones A/B^18^ (Figure S3). Among these compounds, synthetic cotransins prevent Sec61-mediated protein biosynthesis in a relatively substrate-specific manner, while cytotoxic natural products such as apratoxin A, ipomoeassin F, and coibamide A (CbA) inhibit co-translational translocation of a broad range of Sec61 substrates with variable sensitivity.^19^ Interestingly, although these natural products all bind to Sec61α, when screened against the National Cancer Institute 60 (NCI 60) human tumor cell line panel, individual Sec61 inhibitors had distinct profiles.^3^ These findings suggest that the natural products may bind to Sec61α in different ways and/or some cancer cells are differentially sensitive to natural Sec61 inhibitors, resulting in a wide range of cellular responses. Alterations of the CbA structure may improve the substrate specificity of Sec61α inhibition as well as the target selectivity, leading to development of potential drug candidates with favorable biological and physicochemical profiles. Thus, we investigated the structure–activity relationships (SARs) of CbA and derivatives in order to characterize key structural elements required for indispensable binding interactions as well as for attractive bioactivities.

Yao et al. reported an SAR study which demonstrated that the potent cytotoxic activity of CbA was maintained when the two *N*,*O*-dimethylserines [MeSer(Me)] in CbA were replaced with *N*-methylalanines (MeAla), whereas most derivatives with simplified structures were less potent (Figure 1A).^20^ We also previously investigated modification of the CbA macrolactone substructure.^21^ CbA mimetic **2a**, in which the d-MeAla^11^-MeThr^5^ moiety at the ring junction was substituted with a simple MeLys(Me) (Figure 1B), displayed moderate cytotoxic activity.^21^ This lysine-linked CbA mimetic was applicable to our lead optimization campaign, in which we revealed that substitution of Tyr(Me)^10^ in CbA with (biphenylyl)alanine (Bph) led to a significant increase in cytotoxic potential. The modifications of CbA with MeLys(Me) facilitated the synthesis of a series of derivatives to avoid possible racemization and less efficient couplings. However, the cytotoxic potential of **2a** was significantly less than CbA. These data suggested that the MeThr^5^ β-methyl and d-MeAla^11^ α-methyl in CbA would contribute to the cellular bioactivity via the direct interaction with the target molecule(s) and/or other indirect effect(s). The presence of these backbone methyl groups and their configurations alter the local conformations by steric hindrance and may consequently modify the patterns of intramolecular hydrogen bonding to affect the global conformations of peptide macrocycles.^22^ These may further optimize the interaction of the side chain(s) with the target molecule and/or increase the cell membrane permeability. We envisioned that the introduction of two methyl groups in the corresponding positions and stereochemical configurations would improve the cellular bioactivity of CbA mimetics. Initially, we designed two cyclic peptides **3a,b** incorporating novel lysine derivatives at the ring junction, in which two methyl groups corresponding to the MeThr^5^ β-methyl and d-MeAla^11^ α-methyl in CbA are positioned at the lysine β- and ε-positions (Figure 1C).

For the preparation of peptides **3a,b** by a standard Fmoc-based solid-phase peptide synthesis (SPPS), we designed two novel lysine derivatives, **4a,b** (Figure 1D). In order to distinguish two amino groups in the lysine analogues for the site-selective acylation, Fmoc and Alloc groups were employed for the α- and ε-amino groups, respectively. The three configurations of the α-amino, β-methyl, and ε-methyl groups should be selectively constructed for the synthesis of **4a,b**. Concomitant control of the *E*-alkene geometry existing between two asymmetric carbons in **4b** is also needed.

Synthesis of the two lysine analogues began with d-serine-derived Garner aldehyde (Scheme 1).^23^ Initially, Garner aldehyde **5** was subjected to a Wittig reaction with (triphenylphosphoranylidene)acetaldehyde to give α,β-unsaturated aldehyde **6**.^24^*n*-Bu_2_BOTf-mediated Evans aldol reaction of **6** provided the *syn*-aldol product **7** having an α-methyl group which becomes the ε-methyl in **4a,b**.^25,26^ The *S*-configuration of the hydroxy group in **7** is important for the late-stage stereoselective introduction of the β-methyl group in **4a,b**. Protection of the hydroxy group in **7** followed by hydrolytic removal of the chiral auxiliary afforded acid **9**. The stereochemistry of **9** was confirmed by X-ray crystallography (Figure S1). Diphenylphosphoryl azide (DPPA)-mediated conversion of the acid **9** into acyl azide followed by Curtius rearrangement provided an isocyanate intermediate, which was treated with benzyl and allyl alcohols to give Cbz- and Alloc-protected amines **10a,b**, respectively. In this step, the Cbz group was employed as an interim protecting group for the synthesis of **10a** to avoid the loss of function of the Alloc group by the later olefinic hydrogenation. After deprotection of the TBS group, the resulting hydroxy group was activated as mesylates **12a,b**. Introduction by site- and stereoselective *anti*-S_N_2′ alkylation of **12a,b** was achieved using organocopper reagents to provide β-methyl adducts **13a,b**.^27,28^ The configuration at the β-position in **13b** was confirmed by comparative NMR analysis with an authentic sample, which was synthesized via an alternative route (Scheme S1). Olefin hydrogenation in **13a** and simultaneous Cbz deprotection followed by Alloc protection gave lysine precursor **14** with an aminoalkyl side chain. Protecting group manipulations in acetonides **13b** and **14** followed by AZADOL-mediated oxidation of the resulting alcohols **15a,b** afforded the expected lysine derivatives **4a,b**.

**Scheme 1:**
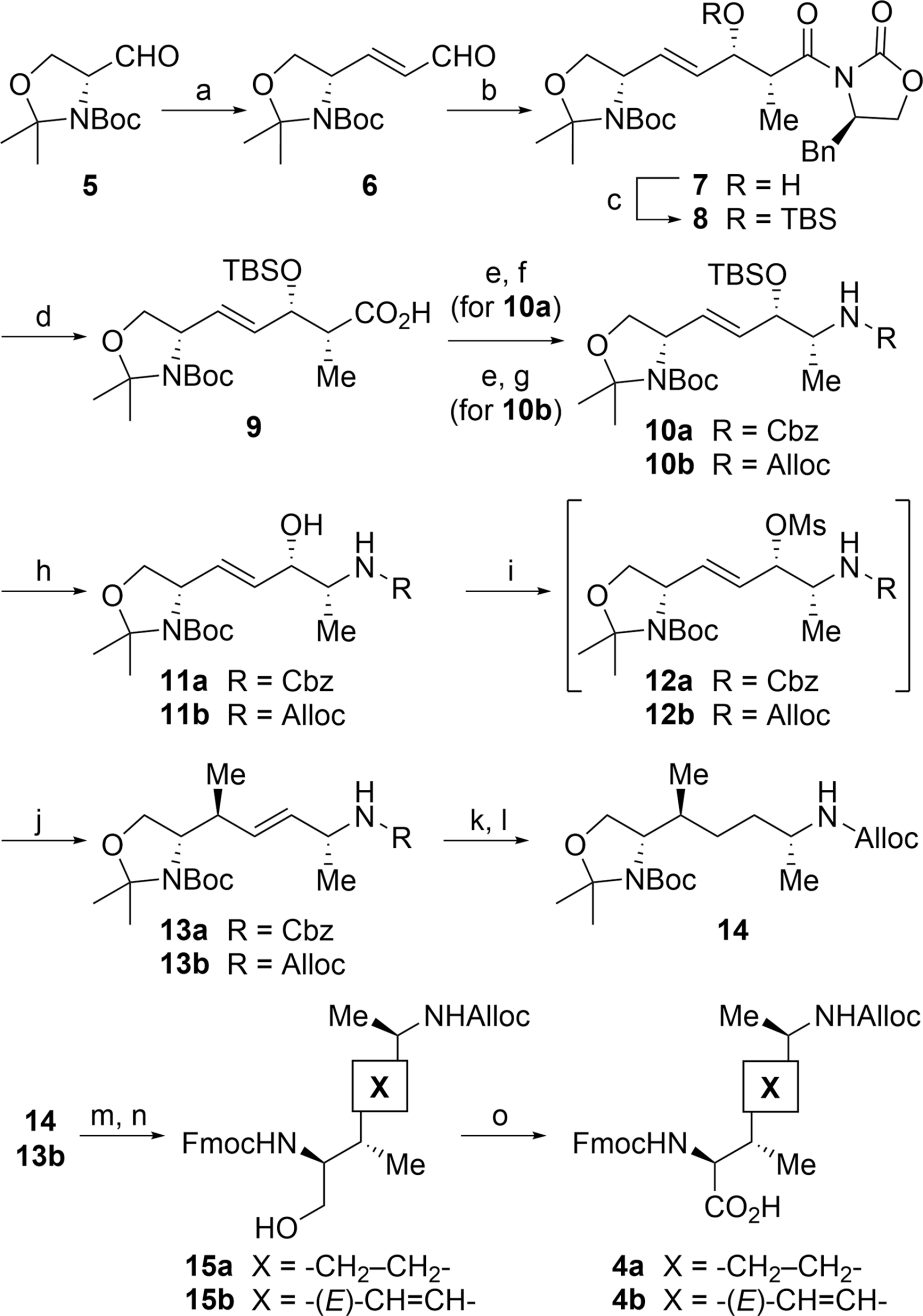
Stereoselective Synthesis of Precursor Lys Derivatives *Reagents and conditions*: (a) Ph_3_P=CHCHO, CH_2_Cl_2_, 20–25 °C, 75%; (b) (*R*)-4-benzyl-3-propanoyl-2-oxazolinone, *n*-Bu_2_BOTf, DIEA, CH_2_Cl_2_, −78 °C to −10 °C, 75%; (c) TBSOTf, 2,6-lutidine, CH_2_Cl_2_, 0 °C to 20–25 °C, 98%; (d) LiOH·H_2_O, H_2_O_2_, THF, H_2_O, 0 °C, 75%; (e) DPPA, Et_3_N, toluene, 50 °C; (f) BnOH, toluene, 50 °C, 84% (2 steps); (g) allyl alcohol, toluene, 50 °C, 90% (2 steps); (h) TBAF, THF, 20–25 °C, 80% (**11a**) and 83% (**11b**); (i) MsCl, pyridine, CHCl_3_, 0 °C; (j) MeMgBr, CuCN, LiCl, THF, −78 °C, 79% (**13a**) and 75% (**13b**) (2 steps); (k) 10% Pd/C, MeOH, 20–25 °C; (l) Alloc-Cl, DIEA, THF, 0 °C to 20–25 °C, 85% (2 steps from **13a**); (m) TFA, CHCl_3_, 0 °C to 20–25 °C; (n) Fmoc-OSu, DIEA, MeCN, H_2_O, 20–25 °C, 71% (**15a**) and 87% (**15b**) (2 steps); (o) AZADOL, NaClO, NaClO_2_, MeCN, phosphate buffer (pH 7.0), 0 °C, 75% (**4a**) and 71% (**4b**).

Next, we synthesized CbA derivatives **3a,b** by Fmoc-based SPPS using Fmoc/Alloc protected lysine derivatives **4a,b**, according to the synthetic procedure for CbA mimetics in our previous study (Scheme S2).^21^ The bioactivity profile of the resulting peptides **3a,b** was investigated by using a standard MTS antiproliferative/cell viability assay to assess the cytotoxic potential of CbA analogues against human A549 lung cancer cells (Table 1). Peptide **3a**, with a 3,6-dimethyllysine moiety at the ring junction, showed enhanced cytotoxic activity compared to that of nonmethylated peptide **2a** [CC_50_ (**3a**) = 41 nM]. Peptide **3b**, with restricted rotation of the C^γ^=C^δ^ double bond in the lysine linker, was equipotent with the parent ester **1b** [CC_50_ (**3b**) = 2.6 nM], and also 10-fold more potent than **3a**. As such, the two β- and ε-methyls in the lysine linkages of **3a,b**, which correspond to the MeThr^5^ β-methyl and d-MeAla^11^ α-methyl in CbA, contribute to potent bioactivity, possibly due to direct interactions with the target molecule and/or an indirect effect on the bioactive conformations. Additionally, the presence of a δ-C(sp^2^) in the lysine linker of **3b** and the corresponding d-MeAla^11^ carbonyl carbon in **1b** would contribute to stabilization of favorable CbA macrocycle conformations.

**Table 1:** Structure–Activity Relationships of CbA Analogues with a Modified Lysine Linker

Peptide	R	X	CC_50_ (nM)^a^	IC_50_ (nM)^b^
**1b**	Me	-CO-O-	3.1 ± 0.8	2.0 ± 0.3
**2a**	H	-CH_2_-CH_2_-	420 ± 30	19 ± 4
**3a**	Me	-CH_2_-CH_2_-	41 ± 4	18 ± 1
**3b**	Me	-(*E*)-CH=CH-	2.6 ± 0.4	13 ± 2

a Cytotoxic concentration (CC_50_) values are the concentrations needed for a 50% decrease in viability of human A549 lung cancer cells relative to the control (*n* = 3).

b Inhibitory concentration (IC_50_) values are the concentrations needed for a 50% inhibition of GLuc expression. The secretory function of U87-GLuc cells was measured after 18-h incubation in the presence or absence of the compound (*n* = 3). At the 18-h end point of the protein secretion assay in U87-GLuc cells, no effect on cell viability was observed at 0.3 nM to 3.0 μM.

Given the favorable effect of limited ring junction flexibility on bioactive conformations of the macrocycle, caused by the ester bond in **1a,b** and olefin in **3b**, we subsequently investigated the SARs of amide moieties at the ring junction. Amides and alkenes have been used as mutually exchangeable isosteres in medicinal chemistry^28−32^ because their planar architecture maintains the parent structure to reproduce desired bioactivities.^33^ Additionally, there have been a number of examples of ester bond substitution to enhance chemical and metabolic stabilities of cyclic depsipeptides with attractive bioactivities.^34−37^

In order to investigate the effect on bioactivity of a macrolactam, we designed and synthesized peptide **16a**, in which an amide bond was substituted for the d-MeAla^11^-MeThr^5^ ester bond in depsipeptide **1b** (Table 2, Scheme S3). Peptide **16a** maintained potent cytotoxic activity equivalent to that exhibited by parent depsipeptide **1b** [CC_50_ (**16a**) = 3.6 nM]. Based on these results, we further designed and synthesized macrolactam CbA analogue **16b**, in which MeThr^5^ of CbA is substituted with *N*^α^-methyl-2,3-diaminobutyric acid (MeDab). Peptide **16b** was also equipotent to natural CbA (**1a**) [CC_50_ (**16b**) = 1.3 nM]. Furthermore, peptide **16c**, which contains an additional substitution of Tyr(Me)^10^ with Bph, was approximately 5-fold more potent than **1a**, and more cytotoxic than macrolactam **16b** [CC_50_ (**16c**) = 0.27 nM]. These results indicate that replacement of the ester bond of d-MeAla^11^-MeThr^5^ in CbA with an amide surrogate(s) maintains the potent cytotoxic activity of coibamide compounds. In contrast, altering the methylation pattern around the ring junction rendered the macrolactam CbA analogues less potent. Use of a β-methyl-deficient *N*^α^-methyl-2,3-diaminopropionic acid (MeDap) in place of MeDab^5^ in **16a** led to a 16-fold decrease in the bioactivity of peptide **17a** [CC_50_ (**17a**) = 48 nM]. *N*^β^-Methylation of MeDap^5^ in peptide **17b** was ineffective in recovering the bioactivity from **17a** [CC_50_ (**17b**) = 6500 nM].

**Table 2:**
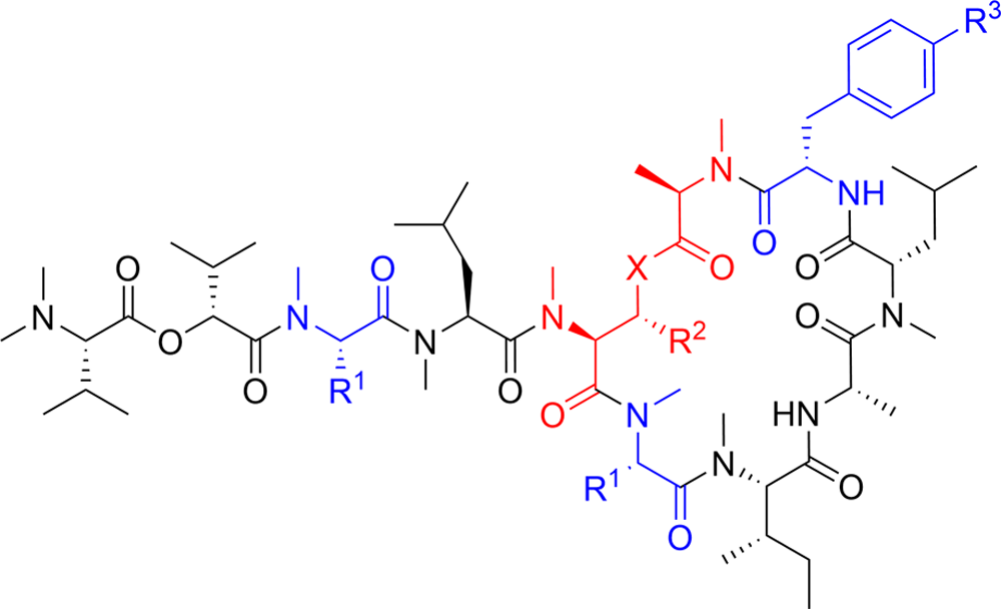
Structure–Activity Relationships of CbA Analogues with an Amide Linkage

Peptide	R^1^	R^2^	X	R^3^	CC_50_ (nM)^a^	IC_50_ (nM)^b^
Coibamide A (**1a**)	CH_2_OMe	Me	-O-	OMe	1.5 ± 0.4	1.1 ± 0.3
	[MeSer(Me)]			[Tyr(Me)]		
**1b**	Me	Me	-O-	OMe	3.1 ± 0.8	2.0 ± 0.3
	[MeAla]			[Tyr(Me)]		
**16a**	Me	Me	-NH-	OMe	3.6 ± 1.4	0.71 ± 0.09
	[MeAla]			[Tyr(Me)]		
**16b**	CH_2_OMe	Me	-NH-	OMe	1.3 ± 0.3	0.04 ± 0.02
	[MeSer(Me)]			[Tyr(Me)]		
**16c**	CH_2_OMe	Me	-NH-	Ph	0.27 ± 0.10	0.08 ± 0.03
	[MeSer(Me)]			(Bph)		
**17a**	Me	H	-NH-	OMe	48 ± 10	30 ± 3.9
	[MeAla]			[Tyr(Me)]		
**17b**	Me	H	-NMe-	OMe	6500	–c
	[MeAla]			[Tyr(Me)]		

a Cytotoxic concentration (CC_50_) values are the concentrations needed for 50% inhibition of human A549 lung cancer cells relative to control (*n* = 3).

b Inhibitory concentration (IC_50_) values are the concentrations needed for 50% inhibition of GLuc expression. The secretory function of U87-GLuc cells was measured after 18-h incubation in the presence or absence of the compound (*n* = 3). At the 18-h end point of the protein secretion assay in U87-GLuc cells, no effect on cell viability was observed at 0.03 to 300 nM for CbA (**1a**) and 0.3 nM to 3.0 μM for all other peptides.

c Not tested.

Thus, it is inferred that substitution of the CbA ester bond with the amide bond in **16a**–**c** does not alter the conformation of these bioactive peptides significantly. The amide NH hydrogen bond donor in **16a**–**c** did not lead to unfavorable interactions or, presumably, a significant change in macrocyclic conformation. In the case of CbA amide analogues, the presence of the β-methyl group in Dab^5^, which corresponds to the MeThr^5^ β-methyl in CbA, was indispensable for potent cytotoxic activity.

Representative CbA peptides from the SAR study were evaluated for cytotoxic activity against two additional human cancer cell lines and normal human dermal fibroblasts (HDFs) (Table S1). Consistent with our previous studies, CbA (**1a**) showed low nanomolar toxicity to HCT116 colon cancer cells and was approximately 30-fold less potent against U87-MG glioblastoma cells.^3,6^ The SAR for cytotoxic potential of the CbA derivatives against HCT116 colon, U87-MG glioblastoma, and normal HDFs was roughly the same as that observed for A549 lung cancer cells employed for the initial SAR analysis, although cell-type specific differences in sensitivity were also observed (Table S1). Among the CbA analogues investigated, Bph^10^ analogue **16c** was the most potent cytotoxic peptide against all three cancer cell lines and HDFs. Peptides **2a** and **17a** with no β-methyl groups at position 5 exhibited less cytotoxicity against all cell lines compared with other peptides possessing two methyl groups at positions corresponding to those of the original d-MeAla^11^-MeThr^5^. Peptides **2a**, **3a**, and **17a** were generally least toxic to normal HDFs although these cells also displayed a distinct pattern of sensitivity (Table S1). Taken together, these data suggest that the appropriate arrangement of the methyl groups around the ring junction plays an important role in cellular bioactivity and that the relative safety of individual peptides against normal cells cannot necessarily be predicted *a priori* due to heterogeneity in the sensitivity of various histological cancer cell types to CbA^1^ and CbA analogues.

To investigate the mechanistic basis for cytotoxicity, all CbA derivatives were evaluated for their ability to inhibit expression of a model Sec61 substrate using U87-MG cells engineered to express a naturally secreted reporter protein, *Gaussia* luciferase (GLuc) (Tables 1 and 2). For these studies, U87-MG-GLuc cells were exposed to increasing concentrations of each peptide, or vehicle, for 18 h, and the cell culture medium was assayed for the presence of GLuc. All CbA derivatives showed concentration dependent inhibition of GLuc secretion (Figure S4). Both CbA (**1a**) and MeAla^3^/MeAla^6^ analogue **1b** showed potent inhibition of GLuc secretion. The methyl-deficient analogue **2a**, and modification of the d-MeAla^11^-MeThr^5^ substructure in CbA with lysine derivatives in peptides **3a,b**, resulted in a slight decrease in potency. Interestingly, peptides **16a**–**c** with an amide surrogate at the ring closure exhibited potent (<1 nM) inhibition of GLuc protein secretion. In particular, peptides **16b** and **16c** which retain the MeSer(Me)^3^/MeSer(Me)^6^ in CbA exhibited more than a 10-fold increase in potency of GLuc inhibition relative to CbA (**1a**). Notably, the methyl-deficient derivatives **2a** and **17a** were less potent inhibitors of GLuc secretion, suggesting that the presence of two methyl groups corresponding to the MeThr^5^ β-methyl and d-MeAla^11^ α-methyl in CbA contribute to functional inhibition of GLuc secretion. Independent evaluation of the ability of peptides **2a**, **3a**, and **16b** to inhibit expression of secreted VEGF-A from human SF-296 glioblastoma cells was also consistent with the pattern of activity observed for GLuc inhibition (Figure S5). All peptides induced statistically significant and concentration-dependent decreases in expression of endogenous VEGF-A; however, a ranked order of decreasing potency was observed as follows: **16b** ≤ **1a** < **3a** < **2a** (Figure S5).

These investigations revealed that there are differences in cytotoxic potential of CbA analogues harboring modifications at ring closure. Further, all CbA analogues inhibited secretion of a GLuc reporter protein in living cells, providing independent confirmation of Sec61 target engagement and additional refinement of the SAR established through basic cell viability assays. We can assume that the enhanced cellular bioactivity of macrolactam derivatives **16b,c**, relative to **1a**, are derived predominantly from potent inhibition of Sec61-dependent secretory function, resulting in broad inhibition of Sec61 substrate biosynthesis and enhanced cytotoxicity.^38^ In contrast, lysine derivatives **3a,b** were over 10-fold less potent inhibitors of GLuc secretion than **1a,b**, and retained the same rank order for inhibition of GLuc secretion and general cytotoxic potency with peptide **3a** showing a better safety profile against normal HDFs than **3b** (Table S1). However, the ability of **2a** to retain GLuc inhibitory potency (where **2a** ≈ **3a**), was not predicted by assessment of cell viability alone (Tables 1 and S1) Thus, peptide **2a** represents an example of the potential to separate functional inhibition of secretory pathway function from overt cytotoxicity by chemical modification. The nonmethylated peptide **2a** inhibited GLuc and VEGF-A secretion at nM concentrations (Figures S4 and S5) yet was 160-fold less toxic to normal HDFs than **1a** (Table S1).

In conclusion, we conducted an SAR study of the d-MeAla^11^-MeThr^5^ substructure in CbA in order to establish alternative macrocycle linkers. Substitution of the ester linkage with an olefin or amide bond reproduced the potent cytotoxicity of CbA. The two methyl groups in this macrocycle linker, which correspond to MeThr^5^ β-methyl and d-MeAla^11^ α-methyl, are critical determinants of CbA bioactivity. Using a sensitive screening assay that reports on cellular secretory function, we established that modification of the ring junction with lysine mimetics preserves the ability of the molecule to inhibit GLuc secretion while inducing less overt cytotoxicity. In contrast, the macrolactam increased both GLuc inhibitory potency and cytotoxic activity relative to the natural product. The ability to interrogate SARs based on functional inhibition of secretion in living cells, in addition to the cytotoxic potential of CbA derivatives, indicates that the appropriate optimization of the macrocyclic scaffold of CbA through chemical synthesis could eventually lead to the development of more selective, less cytotoxic protein secretion inhibitors. Further SAR investigations as well as the investigations of the binding mode of CbA analogues are ongoing.

## Supporting Information Available

Experimental procedures of synthesis and biological evaluations, characterization data, and supporting figures (PDF)

## Abbreviations

Alloc: allyloxycarbonyl
ATG5: authophagy-related protein 5
Bph: β-(4-biphenylyl)alanine
CbA: coibamide A
GLuc: *Gaussia* luciferase
HDF: human dermal fibroblast
HER: human epidermal growth factor receptor
Hva: 2-hydroxyisovaleric acid
Me_2_Val: *N*,*N*-dimethylvaline
MeAla: *N*-methylalanine
MeLys(Me): *N*,*N′*-dimethyllysine
MeSer(Me): *N*,*O*-dimethylserine
MeThr: *N*-methylthreonine
mTOR: mammalian target of rapamycin
SPPS: solid-phase peptide synthesis
Tyr(Me): *O*-methyltyrosine
VEGF-A: vascular endothelial growth factor A
VEGFR2: vascular endothelial growth factor receptor 2
