# Supplementary material for: Design of Synthetic Surrogates for the Macrolactone Linker Motif in Coibamide A
Source: ACS Med Chem Lett. 2023 Sep 19;14(10):1344–50. doi: 10.1021/acsmedchemlett.3c00232 (PMC10578308; doi:10.1021/acsmedchemlett.3c00232)
Supplement: Supplementary file 1 — ml3c00232_si_001.pdf [file ml3c00232_si_001.pdf]

Supporting Information

Design of Synthetic Surrogates for the Macrolactone Linker Motif in Coibamide A

Rikito Suzuki,[1,2] Daphne R. Mattos,[3] Takashi Kitamura,[1] Rina Tsujioka,[2] Kazuya Kobayashi,[2] Shinsuke Inuki,[1] Hiroaki Ohno,[1] Jane E. Ishmael,[3] Kerry L. McPhail,[3] and Shinya Oishi*,[1,2]

[1]*Graduate School of Pharmaceutical Sciences, Kyoto University, Sakyo-ku, Kyoto 606-8501, Japan*

[2]*Laboratory of Medicinal Chemistry, Kyoto Pharmaceutical University, Yamashina-ku, Kyoto 607-8412, Japan*

[3]*Department of Pharmaceutical Sciences, College of Pharmacy, Oregon State University, Corvallis, Oregon 97331, United States*

E-mail: soishi@mb.kyoto-phu.ac.jp

<u>*Table of Contents*</u>

Experimental section	S2
Structures of Sec61 inhibitors	S23
Cytotoxic activities of coibamide A derivatives	S24
Inhibition of secretory function in viable cells by coibamide A derivatives	S25
Detection of secreted VEGF-A from human SF-268 glioblastoma cells	S26
NMR spectra	S27

Experimental section

General

^{1}H and ^{13}C NMR spectra were recorded using a JEOL ECA-500 or JEOL ECZ600R spectrometer. Chemical shifts are reported in δ (ppm), relative to Me_4Si (in $CDCl_3$) as an internal standard for ^{1}H, and referenced to the residual solvent signal for ^{13}C (δ 77.0). Exact mass (HRMS) data were recorded on a JEOL SX-102A (FAB) or Shimadzu LC-ESI-IT-TOF-MS (ESI-TOF) equipment. Optical rotations were measured using a JASCO P-1020 polarimeter. For flash chromatography, Wakogel C-300E (Wako) was employed. For analytical HPLC, a Cosmosil 5C18-ARII column (4.6 × 250 mm, Nacalai Tesque, Inc.) was employed with a linear gradient of CH_3CN (with 0.05% (v/v) TFA or with 0.1% (v/v) TFA) in H_2O, and eluting products were detected by UV at 220 nm. Preparative HPLC was performed using a Cosmosil 5C18-ARII preparative column (20 × 250 mm, Nacalai Tesque, Inc.). The compound purity for the bioassays was determined to be >90% by HPLC analysis. No unexpected or unusually high safety hazards were encountered.

***tert*-Butyl (*S,E*)-2,2-dimethyl-4-(3-oxoprop-1-en-1-yl)oxazolidine-3-carboxylate (6).** To a stirred solution of D-Garner aldehyde **5** (1.00 g, 4.34 mmol) in dry CH_2Cl_2 (7 mL) was added triphenylphosphoranylidene-acetaldehyde (1.34 g, 4.34 mmol) at 20–25 °C. The reaction mixture was stirred for 46 h. The solution was concentrated and the residue was purified by flash chromatography over silica gel (hexane/EtOAc = 7/1) to give compound **6** (0.836 g, 75%) as a yellow oil. The spectral data were in good agreement with those previous reported.[22]

***tert*-Butyl (*S*)-4-{(3*S*,4*R*,*E*)-5-[(*R*)-4-benzyl-2-oxooxazolidin-3-yl]-3-hydroxy-4-methyl-5-oxopent-1-en-1-yl}-2,2-dimethyloxazolidine-3-carboxylate (7).** To a stirred solution of (*R*)-4-benzyl-3-propionyl-2-oxazolidinone (754 mg, 3.23 mmol) in dry CH_2Cl_2 (24 mL) under argon were added *n*-Bu_2BOTf (1.0 M in CH_2Cl_2; 3.88 mL, 3.88 mmol) and DIEA (676 μL, 3.88 mmol) at –78 °C. After stirring for 1 h, the reaction mixture was warmed to 0 °C and stirred for another 30 min. To this mixture was added a solution of **6** (826 mg, 3.23 mmol) in CH_2Cl_2 (8 mL) at –78 °C. After stirring for 1 h, the mixture was warmed to –10 °C and stirred for another 1 h. The mixture was diluted with pH 7.0 phosphate buffer solution (4 mL) and 30% H_2O_2 in MeOH (1:2, 12 mL) and stirred overnight. The whole was concentrated under reduced pressure and extracted with Et_2O. The extract was washed with aqueous saturated $NaHCO_3$ and brine, and dried over Na_2SO_4. The filtrate was concentrated under reduced pressure and the residue was purified by flash chromatography over silica gel (hexane/EtOAc = 4/1 to 2/1) to give compound **7** (1.18 g, 75%) as a colorless oil: $[\alpha]^{26}_{D}$ –28.1 (*c* 1.02, $CHCl_3$); IR

(neat): 3496 (OH), 1778 (C=O), 1690 (C=O); ^{1}H NMR (500 MHz, $CDCl_3$, mixture of rotamers) δ: 1.24 (d, *J* = 7.0 Hz, 3H), 1.45 (s, 9H), 1.45 (s, 1.5H), 1.51 (s, 1.5H), 1.57 (s, 1.5H), 1.62 (s, 1.5H), 2.79 (dd, *J* = 13.4, 9.5 Hz, 1H), 2.82–2.94 (m, 1H), 3.25 (dd, *J* = 13.4, 3.2 Hz, 1H), 3.75 (dd, *J* = 8.8, 1.6 Hz, 1H), 3.80–3.92 (m, 1H), 4.00–4.06 (m, 1H), 4.15–4.27 (m, 2H), 4.28–4.32 (m, 0.5H), 4.38–4.45 (m, 0.5H), 4.47–4.56 (m, 1H), 4.65–4.74 (m, 1H), 5.56–5.69 (m, 1H), 5.72–5.81 (m, 1H), 7.20 (d, *J* = 7.1 Hz, 2H), 7.28 (m, 1H), 7.33 (m, 2H); ^{13}C{^{1}H} NMR (125 MHz, $CDCl_3$, mixture of rotamers) δ: 10.9, 11.2, 23.5, 24.8, 26.7, 27.4, 28.4, 37.8, 42.4, 42.6, 53.8, 55.1, 58.5, 58.6, 66.2, 67.9, 68.2, 71.3, 71.9, 79.7, 94.0, 127.4, 128.9, 129.0, 129.4, 130.4, 131.1, 131.3, 134.9, 151.9, 153.0, 176.7; HRMS (ESI-TOF) *m/z*: [M + Na]$^+$ calcd for $C_{26}H_{36}N_2NaO_7$, 511.2415; found: 511.2415.

***tert*-Butyl (*S*)-4-{(3*S*,4*R*,*E*)-5[(*R*)-4-benzyl-2-oxooxazolidin-3-yl]-3-[(*tert*-butyldimethylsilyl)oxy]-4-methyl-5-oxopent-1-en-1-yl}-2,2-dimethyloxazolidine-3-carboxylate (8).** To a stirred solution of **7** (2.03 g, 4.16 mmol) in dry CH_2Cl_2 (16 mL) under argon were added TBSOTf (1.15 mL, 5.00 mmol) and 2,6-lutidine (1.74 mL, 17.0 mmol) at 0 °C. The reaction mixture was warmed to 20–25 °C and stirred for 2 h. The reaction was quenched with aqueous saturated $NaHCO_3$. The whole was extract with EtOAc and the extract was washed with saturated citric acid and brine, and dried over Na_2SO_4. The filtrate was concentrated under reduced pressure and the residue was purified by flash chromatography over silica gel (hexane/EtOAc = 3/1) to give compound **8** (2.45 g, 98%) as a colorless oil: $[\alpha]^{24}_{D}$ –7.5 (*c* 1.02, $CHCl_3$); IR (neat): 1782 (C=O), 1696 (C=O); ^{1}H NMR (500 MHz, $CDCl_3$, mixture of rotamers) δ: 0.01 (s, 3H), 0.01 (s, 3H), 0.88 (s, 9H), 1.21 (d, *J* = 6.5 Hz, 3H), 1.45 (s, 9H), 1.47 (s, 1.5H), 1.51 (s, 1.5H), 1.55 (s, 1.5H), 1.59 (s, 1.5H), 2.73–2.82 (m, 1H), 3.23–3.31 (m, 1H), 3.65–3.72 (m, 1H), 3.87–3.97 (m, 1H), 4.00 (m, 1H), 4.11–4.18 (m, 1.5H), 4.28 (m, 0.5H), 4.31–4.42 (m, 2H), 4.55–4.63 (m, 1H), 5.60–5.74 (m, 2H), 7.21 (d, *J* = 7.1 Hz, 2H), 7.28 (m, 1H), 7.33 (dd, *J* = 7.3, 7.3 Hz, 2H); ^{13}C{^{1}H} NMR (125 MHz, $CDCl_3$, mixture of rotamers) δ: −5.2, −4.3, −0.03, 11.9, 12.7, 18.1, 23.4, 24.7, 25.7, 26.7, 26.9, 27.3, 28.4, 37.7, 41.9, 44.4, 55.6, 55.7, 58.0, 66.0, 67.8, 68.6, 73.2, 74.5, 79.6, 79.9, 93.5, 93.8, 127.2, 128.9, 129.1, 129.4, 130.2, 131.2, 131.9, 132.1, 135.3, 135.5, 151.7, 153.2, 174.3, 174.8; HRMS (ESI-TOF) *m/z*: [M + Na]$^+$ calcd for $C_{32}H_{50}N_2NaO_7Si$, 625.3279; found: 625.3273.

(2*R*,3*S*,*E*)-5-[(*S*)-3-(*tert*-Butoxycarbonyl)-2,2-dimethyloxazolidin-4-yl]-3-[(*tert*-butyldimethyl-silyl)oxy]-2-methylpent-4-enoic acid (9). To a stirred solution of **8** (84 mg, 0.14 mmol) in THF (2 mL) and H_2O (0.5 mL) were added LiOH·H_2O (11.8 mg, 0.281 mmol) and 30% H_2O_2 in H_2O (100 µL) at 0 °C. The reaction mixture was stirred at the same temperature for 4 h. The reaction was quenched with aqueous saturated Na_2SO_3. The mixture was acidified with saturated citric acid to pH

3 and extracted with EtOAc. The extract was washed with water and brine, and dried over Na_2SO_4. The filtrate was concentrated under reduced pressure and the residue was purified by flash chromatography over silica gel (hexane/EtOAc = 3/1) to give compound **9** (46.6 mg, 75%) as colorless block crystals: mp 105−109 °C; $[\alpha]^{25}_{D}$ +40.4 (*c* 1.04, $CHCl_3$); IR (neat): 1703 (C=O); ^{1}H NMR (500 MHz, $CDCl_3$, mixture of rotamers) δ: 0.07 (s, 4.5H), 0.09 (s, 1.5H), 0.90 (br s, 9H), 1.10 (br s, 3H), 1.41–1.62 (m, 15H), 2.58–2.66 (m, 1H), 3.69 (m, 1H), 3.99–4.04 (m, 1H), 4.28–4.34 (m, 0.5H), 4.36–4.42 (m, 1.5H), 5.51–5.75 (m, 2H); ^{13}C{^{1}H} NMR (150 MHz, $CDCl_3$, mixture of rotamers) δ: –5.3, –5.2, –4.2, 0.0, 11.3, 11.6, 18.5, 23.3, 24.7, 25.7, 26.8, 27.5, 28.4, 45.4, 45.5, 58.1, 58.2, 67.8, 68.4, 74.2, 74.6, 79.8, 80.2, 93.4, 94.3, 129.9, 130.1, 131.8, 132.7, 151.7, 151.8, 176.4; HRMS (ESI-TOF) *m/z*: $[M + Na]^+$ calcd for $C_{22}H_{41}NNaO_6Si$, 466.2595; found: 466.2594.

X-ray Crystallographic Analysis of 9 (Figure S1)

The data of the compound **9** ($C_{22}H_{41}NO_6Si$) was collected with a Rigaku XtaLAB AFC11 (RCD3): quarter-chi single diffractometer using mirror monochromated Cu*Kα* radiation at 93 K. The substance was crystallized from *n*-hexane as colorless block crystals and solved in monoclinic space group $P2_1/_n$ with Z = 2. The structure was solved by direct methods (SHELXT-2018/2) and refined by the full-matrix least-squares on F2 (SHELXL-2018/3). All non-hydrogen atoms were refined anisotropically and all hydrogen atoms were placed using AFIX instructions. The crystal data are as follows: $C_{22}H_{41}NO_6Si$, FW: 443.65. a = 6.7065(1), b = 22.0348(2), c = 9.0980(1), V =1293.05(3) Å^3, Z = 2, D_{calc} = 1.139 g/cm^3, μ = 1.078 mm^{-1}, R_1 = 0.0313 ($I > 2\sigma(I)$), wR_2 = 0.0895 (all data), GOF = 1.057. The CCDC deposition number: CCDC 2239668.

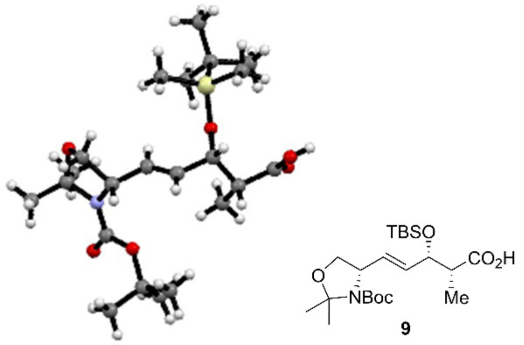

Figure S1. Crystal structure of carboxylic acid **9**.

***tert*-Butyl (*S*)-4-[(3*S*,4*R*,*E*)-4-{[(benzyloxy)carbonyl]amino}-3-[(*tert*-butyldimethylsilyl)oxy]-pent-1-en-1-yl]-2,2-dimethyloxazolidine-3-carboxylate (10a).** To a stirred solution of **9** (518 mg, 1.17 mmol) in dry toluene (5 mL) were added diphenylphosphoryl azide (377 µL, 1.75 mmol) and triethylamine (488 µL, 3.51 mmol) at 20–25 °C. The reaction mixture was warmed to 50 °C and stirred

for 24 h. To this mixture was added benzyl alcohol (602 μL, 5.84 mmol) at 50 °C. The reaction mixture was stirred at the same temperature for 24 h. Then, water was added to the mixture at 20–25 °C. The whole was extracted with EtOAc and the extract was washed with brine, and dried over Na_2SO_4. The filtrate was concentrated under reduced pressure and the residue was purified by flash chromatography over silica gel (hexane/EtOAc = 5/1) to give compound **10a** (540 mg, 84%) as a colorless oil: $[\alpha]^{25}_{D}$ +56.8 (*c* 1.02, $CHCl_3$); IR (neat): 1699 (C=O); ^1H NMR (500 MHz, $CDCl_3$, mixture of rotamers) δ: 0.00 (s, 6H), 0.89 (s, 9H), 1.06 (d, J = 6.3 Hz, 3H), 1.43 (s, 9H), 1.48–1.60 (m, 6H), 3.69 (d, J = 8.6 Hz, 1H), 3.75–4.02 (m, 2H), 4.27–4.39 (m, 2H), 4.78–4.87 (m, 1H), 5.08 (s, 2H), 5.53–5.71 (m, 2H), 7.33 (m, 5H); $^{13}C\{^1H\}$ NMR (150 MHz, $CDCl_3$, mixture of rotamers) δ: –5.1, –4.5, 13.9, 14.5, 18.2, 23.4, 25.0, 25.8, 26.7, 28.4, 28.4, 51.3, 51.5, 58.3, 58.5, 66.4, 66.5, 67.8, 68.4, 73.8, 74.0, 79.6, 80.0, 93.3, 94.0, 127.9, 128.1, 128.5, 130.1, 131.0, 131.9, 136.6, 151.8, 155.7; HRMS (ESI-TOF) *m/z*: [M + Na]$^+$ calcd for $C_{29}H_{48}N_2NaO_6Si$, 571.3174; found: 571.3170.

***tert*-Butyl (*S*)-4-[(3*S*,4*R*,*E*)-4-{[(allyloxy)carbonyl]amino}-3-[(*tert*-butyldimethylsilyl)oxy]pent-1-en-1-yl]-2,2-dimethyloxazolidine-3-carboxylate (10b).** To a stirred solution of **9** (1.07 g, 2.41 mmol) in dry toluene (10 mL) were added diphenylphosphoryl azide (622 μL, 2.89 mmol) and triethylamine (1.01 mL, 7.24 mmol) at 20–25 °C. The reaction mixture was warmed to 50 °C and stirred for 24 h. To this mixture was added allyl alcohol (828 μL, 12.1 mmol) at 50 °C. The reaction mixture was stirred at the same temperature for 24 h. Then, water was added to the mixture at 20–25 °C. The whole was extracted with EtOAc and the extract was washed with brine, and dried over Na_2SO_4. The filtrate was concentrated under reduced pressure and the residue was purified by flash chromatography over silica gel (hexane/EtOAc = 5/1) to give compound **10b** (1.08 g, 90%) as a colorless oil: $[\alpha]^{25}_{D}$ +62.5 (*c* 1.01, $CHCl_3$); IR (neat): 1698 (C=O); ^1H NMR (500 MHz, $CDCl_3$, mixture of rotamers) δ: 0.02 (s, 3H), 0.04 (s, 3H), 0.91 (s, 9H), 1.06 (d, J = 6.3 Hz, 3H), 1.39–1.64 (m, 15H), 3.68–3.78 (m, 2H), 4.02 (m, 1H), 4.26–4.42 (m, 2H), 4.55 (m, 2H), 4.76–4.90 (m, 1H), 5.16–5.23 (m, 1H), 5.29 (d, J = 17.2 Hz, 1H), 5.50–5.59 (m, 0.5H), 5.60–5.78 (m, 1.5H), 5.85–5.97 (m, 1H); $^{13}C\{^1H\}$ NMR (125 MHz, $CDCl_3$, mixture of rotamers) δ: –5.2, –4.5, 13.9, 14.5, 18.1, 23.4, 24.9, 25.8, 25.8, 26.6, 27.4, 28.4, 51.2, 51.4, 58.3, 58.5, 65.2, 65.3, 67.7, 68.4, 73.8, 74.0, 79.6, 80.0, 93.3, 93.9, 117.3, 117.5, 130.0, 131.0, 131.9, 132.9, 133.1, 151.8, 155.5; HRMS (FAB) *m/z*: [M + H]$^+$ calcd for $C_{25}H_{47}N_2O_6Si$, 499.3198; found: 499.3202.

***tert*-Butyl (*S*)-4-[(3*S*,4*R*,*E*)-4-{[(benzyloxy)carbonyl]amino}-3-hydroxypent-1-en-1-yl]-2,2-dimethyloxazolidine-3-carboxylate (11a).** To a stirred solution of **10a** (582 mg, 1.06 mmol) in THF (5 mL) under argon was added TBAF (1.0 M solution in THF; 5.31 mL, 5.31mmol) at 20–25 °C. After

being stirred for 1 h, the reaction mixture was diluted with aqueous saturated NH_4Cl. The whole was extracted with Et_2O and the extract was washed with brine, and dried over Na_2SO_4. The filtrate was concentrated under reduced pressure and the residue was purified by flash chromatography over silica gel (hexane/EtOAc = 2/1) to give compound **11a** (369 mg, 80%) as a colorless oil: $[\alpha]^{24}_{D}$ +36.8 (*c* 1.02, $CHCl_3$); IR (neat): 3353 (OH), 1697 (C=O); ^{1}H NMR (500 MHz, $CDCl_3$, mixture of rotamers) δ: 1.12 (d, *J* = 6.9 Hz, 3H), 1.42 (s, 9H), 1.46–1.63 (m, 6H), 2.27–2.56 (m, 1H), 3.68-3.75 (m, 1H), 3.80–3.99 (m, 1H), 4.03 (dd, *J* = 9.2, 6.3 Hz, 1H), 4.22–4.39 (m, 2H), 4.89–5.19 (m, 3H), 5.53–5.79 (m, 2H), 7.28–7.39 (m, 5H); ^{13}C{^{1}H} NMR (150 MHz, $CDCl_3$, mixture of rotamers) δ: 14.9, 15.3, 15.9, 23.5, 25.1, 26.7, 27.1, 28.3, 28.5, 51.2, 51.4, 58.6, 59.1, 65.9, 66.7, 67.8, 68.3, 73.8, 74.3, 79.8, 80.3, 93.6, 94.1, 128.1, 128.2, 128.5, 130.2, 130.4, 131.0, 131.8, 136.5, 151.8, 152.1, 156.9; HRMS (FAB) *m/z*: $[M + H]^+$ calcd for $C_{23}H_{35}N_2O_6$, 435.2490; found: 435.2505.

***tert*-Butyl (*S*)-4-[(3*S*,4*R*,*E*)-4-{[(allyloxy)carbonyl]amino}-3-hydroxypent-1-en-1-yl]-2,2-dimethyloxazolidine-3-carboxylate (11b).** To a stirred solution of **10b** (1.40 g, 2.81 mmol) in THF (14 mL) under argon was added TBAF (1.0 M solution in THF; 14.0 mL, 14.0 mmol) at 20–25 °C. After being stirred for 1 h, the reaction mixture was diluted with aqueous saturated NH_4Cl. The whole was extracted with Et_2O and the extract was washed with brine, and dried over Na_2SO_4. The filtrate was concentrated under reduced pressure and the residue was purified by flash chromatography over silica gel (hexane/EtOAc = 2/1) to give compound **11b** (895 mg, 83%) as a colorless oil: $[\alpha]^{25}_{D}$ +43.5 (*c* 1.00, $CHCl_3$); IR (neat): 3361 (OH), 1694 (C=O); ^{1}H NMR (500 MHz, $CDCl_3$, mixture of rotamers) δ: 1.11 (d, *J* = 6.9 Hz, 3H), 1.38–1.64 (m, 15H), 2.56– 2.80 (m, 1H), 3.69–3.74 (m, 1H), 3.77-3.95 (m, 1H), 4.03 (dd, *J* = 8.9, 6.2 Hz, 1H), 4.21–4.26 (m, 1H), 4.26–4.39 (m, 1H), 4.48–4.59 (m, 2H), 4.98–5.21 (m, 3H), 5.53–5.79 (m, 2H), 5.83–5.94 (m, 1H); ^{13}C{^{1}H} NMR (125 MHz, $CDCl_3$, mixture of rotamers) δ: 14.9, 15.8, 23.5, 25.1, 26.7, 27.1, 28.4, 51.1, 51.4, 58.6, 59.1, 65.5, 67.8, 68.2, 73.8, 74.2, 79.7, 80.3, 93.6, 94.0, 117.6, 117.8, 130.2, 130.5, 131.1, 131.6, 132.7, 132.9, 151.8, 152.1, 156.2, 156.7; HRMS (ESI-TOF) *m/z*: $[M + Na]^+$ calcd for $C_{19}H_{32}N_2NaO_6$, 407.2153; found: 407.2152.

***tert*-Butyl (*S*)-4-[(2*S*,5*R*,*E*)-5-{[(benzyloxy)carbonyl]amino}hex-3-en-2-yl]-2,2-dimethyloxazolidine-3-carboxylate (13a).** To a stirred solution of **11a** (257 mg, 0.591 mmol) in $CHCl_3$ (1 mL) under argon were added pyridine (952 μL, 11.8 mmol) and MsCl (458 μL, 5.91 mmol) at 0 °C. After being stirred for 2 h, the reaction mixture was diluted with water. The whole was extracted with EtOAc and extract was washed with saturated citric acid and brine, and dried over Na_2SO_4. The filtrate was concentrated under reduced pressure to give compound **12a**, which was used further purification. To a stirred solution of LiCl (250 mg, 5.91 mmol) and CuCN (265 mg, 2.95 mmol) in dry THF (3 mL)

under argon was added MeMgBr (1.02 M solution in THF; 2.90 mL, 2.95 mmol) at –78 °C. The reaction mixture was warmed to 0 °C and stirred for 15 min. To this mixture was added a solution of **12a** in dry THF (2 mL) at –78 °C. After being stirred for 30 min, the reaction mixture was diluted with aqueous saturated NH_4Cl and 28% ammonia solution. The whole was extracted with EtOAc and the extract was washed with brine, and dried over Na_2SO_4. The filtrate was concentrated under reduced pressure and the residue was purified by flash chromatography over silica gel (hexane/EtOAc = 2/1) to give compound **13a** (202 mg, 79%) as a colorless oil: $[\alpha]^{25}_D$ +7.5 (*c* 1.00, $CHCl_3$); IR (neat): 1698 (C=O); ^{1}H NMR (300 MHz, DMSO-d_6, 323 K) δ: 0.89 (d, J = 6.9 Hz, 3H), 1.11 (d, J = 6.8 Hz, 3H), 1.39 (s, 3H), 1.41 (s, 9H), 1.48 (s, 3H), 2.54–2.65 (m, 1H), 3.66–3.86 (m, 3H), 3.96–4.11 (m, 1H), 5.01 (s, 2H), 5.33–5.50 (m, 2H), 7.08 (br s, 1H), 7.26–7.41 (m, 5H); ^{13}C{^{1}H} NMR (150 MHz, $CDCl_3$, mixture of rotamers) δ: 14.3, 15.0, 21.0, 21.3, 22.8, 24.3, 26.0, 27.0, 28.4, 28.5, 38.7, 48.0, 48.4, 60.8, 61.2, 64.4, 64.5, 66.5, 79.7, 80.1, 93.6, 94.2, 128.1, 128.1, 128.5, 131.6, 132.1, 132.8, 136.6, 152.3, 152.8, 155.4; HRMS (FAB) *m/z*: $[M + H]^+$ calcd for $C_{24}H_{37}N_2O_5$, 433.2697; found: 433.2715.

***tert*-Butyl (*S*)-4-[(2*S*,5*R*,*E*)-5-{[(allyloxy)carbonyl]amino}hex-3-en-2-yl]-2,2-dimethyloxazolidine-3-carboxylate (13b).** To a stirred solution of **11b** (438 mg, 1.14 mmol) in $CHCl_3$ (2 mL) under argon were added pyridine (1.83 mL, 22.8 mmol) and MsCl (881 μL, 11.4 mmol) at 0 °C. After being stirred for 2 h, the reaction mixture was diluted with water. The whole was extracted with EtOAc and extract was washed with saturated citric acid and brine, and dried over Na_2SO_4. The filtrate was concentrated under reduced pressure to give compound **12b**, which was used further purification. To a stirred solution of LiCl (482 mg, 11.4 mmol) and CuCN (510 mg, 5.69 mmol) in distilled THF (6 mL) under argon was added MeMgBr (1.02 M solution in THF; 5.57 mL, 5.69 mmol) at –78 °C. The reaction mixture was warmed to 0 °C and stirred 15 min. To this mixture was added a solution of **12b** in dry THF (4 mL) at –78 °C. After being stirred for 30 min, the reaction mixture was diluted with aqueous saturated NH_4Cl and 28% ammonia solution. The whole was extracted with EtOAc and the extract was washed with brine, and dried over Na_2SO_4. The filtrate was concentrated under reduced pressure and the residue was purified by flash chromatography over silica gel (hexane/EtOAc = 2/1) to give compound **13b** (327 mg, 75%) as a colorless oil: $[\alpha]^{25}_D$ +16.9 (*c* 1.00, $CHCl_3$); IR (neat): 1698 (C=O); ^{1}H NMR (300 MHz, DMSO-d_6, 323 K) δ: 0.90 (d, J = 6.9 Hz, 3H), 1.10 (d, J = 6.8 Hz, 3H), 1.39 (s, 3H), 1.41 (s, 9H), 1.48 (s, 3H), 2.54–2.65 (m, 1H), 3.67–3.88 (m, 3H), 3.95–4.08 (m, 1H), 4.42–4.48 (m, 2H), 5.12–5.19 (m, 1H), 5.22–5.31 (m, 1H), 5.34–5.50 (m, 2H), 5.82–5.98 (m, 1H), 7.02 (br s, 1H); ^{13}C{^{1}H} NMR (150 MHz, $CDCl_3$, mixture of rotamers) δ: 14.3, 15.0, 21.0, 21.3, 22.8, 24.3, 26.0, 27.0, 28.4, 28.5, 38.7, 48.0, 48.4, 60.8, 61.2, 64.5, 64.5, 65.4, 79.7, 80.1, 93.6, 94.3, 117.5, 131.6, 132.1, 132.7, 133.0, 152.3, 152.8, 155.3; HRMS (ESI-TOF) *m/z*: $[M + Na]^+$ calcd for $C_{20}H_{34}N_2NaO_5$,

405.2360; found: 405.2360.

***tert*-Butyl (*S*)-4-[(2*S*,5*R*)-5-{[(allyloxy)carbonyl]amino}hexan-2-yl]-2,2-dimethyloxazolidine-3-carboxylate (14).** To a stirred solution of **13a** (170 mg, 0.393 mmmol) in MeOH (17 mL) was added 10% Pd/C (34 mg) at 20–25 °C, and the mixture was flushed with H_2 gas (1 atm). After being stirred for 1 h, the reaction mixture was filtered through membrane filter. The filtrate was concentrated under reduced pressure to give the corresponding amine, which was used without further purification. To a stirred solution of the above amine in THF were added allyl chloroformate (49.9 μL, 0.472 mmol) and DIEA (82.2 μL, 0.472 mmol) at 0 °C. After being stirred for 1 h, the reaction mixture warmed to 20–25 °C and stirred for 2 h. Water was added to this mixture at 20–25 °C. The whole was extracted with EtOAc and the extract was washed with brine, and dried over Na_2SO_4. The filtrate was concentrated under reduced pressure and the residue was purified by flash chromatography over silica gel (hexane/EtOAc = 2/1) to give compound **14** (129 mg, 85%) as a colorless oil: $[\alpha]^{24}_{D}$ +2.6 (*c* 1.02, $CHCl_3$); IR (neat): 1697 (C=O); ^{1}H NMR (500 MHz, $CDCl_3$, mixture of rotamers) δ: 0.86 (d, *J* = 6.8 Hz, 3H), 1.09–1.31 (m, 5H), 1.35–1.64 (m, 17H), 1.85–2.08 (m, 1H), 3.56–3.71 (m, 1H), 3.72–3.91 (m, 3H), 4.43–4.66 (m, 3H), 5.20 (d, *J* = 10.3 Hz, 1H), 5.29 (d, *J* = 17.2 Hz, 1H), 5.84−5.97 (m, 1H); ^{13}C{^{1}H} NMR (150 MHz, $CDCl_3$, mixture of rotamers) δ: 14.4, 14.5, 21.5, 22.8, 24.3, 26.1, 26.9, 28.5, 30.1, 30.3, 34.3, 34.6, 35.3, 35.4, 47.3, 47.6, 60.8, 61.2, 63.7, 63.9, 65.3, 79.6, 80.1, 93.5, 94.0, 117.5, 117.6, 133.0, 133.1, 152.4, 153.0, 155.7; HRMS (FAB) *m/z*: $[M + H]^+$ calcd for $C_{20}H_{37}N_2O_5$, 385.2697; found: 385.2702.

(9*H*-Fluoren-9-yl)methyl allyl [(2*S*,3*S*,6*R*)-1-hydroxy-3-methylheptane-2,6-diyl]dicarbamate (15a). To a stirred solution of **14** (108 mg, 0.281 mmol) in CH_2Cl_2 (1 mL) was added TFA/CH_2Cl_2 (1:3, 4 mL) at 0 °C. After being stirred for 30 min, the mixture was warmed to 20–25 °C and stirred for 1 h. The mixture was concentrated under reduced pressure to give the corresponding amine, which was used without further purification. To a stirred solution of the above amine in MeCN (2 mL) and H_2O (2 mL) were added Fmoc-OSu (123 mg, 0.365 mmol) in MeCN (1 mL) and DIEA (196 μL, 1.12 mmol) at 20–25 °C. The reaction mixture was stirred same temperature for 2 h. Then, water was added to the mixture at 20–25 °C. The whole was extracted with EtOAc and the extract was washed with saturated citric acid and brine, and dried over Na_2SO_4. The filtrate was concentrated under reduced pressure and the residue was purified by flash chromatography over silica gel (hexane/EtOAc = 1/1) to give compound **15a** (92.7 mg, 71%) as a white amorphous solid: $[\alpha]^{25}_{D}$ –14.6 (*c* 0.80, DMF); IR (neat): 3321 (OH), 1698 (C=O); ^{1}H NMR (300 MHz, DMSO-d_6, 323K) δ: 0.83 (d, *J* = 6.3 Hz, 3H), 1.00 (m, 4H), 1.21–1.68 (m, 4H), 3.28–3.51 (m, 4H), 4.17–4.39 (m, 4H), 4.40–4.47 (m, 2H), 5.01–

5.17 (m, 1H), 5.19–5.30 (m, 1H), 5.81–5.96 (m, 1H), 6.77–6.93 (m, 2H), 7.27–7.36 (m, 2H), 7.36–7.45 (m, 2H), 7.70 (d, J = 7.3 Hz, 2H), 7.87 (d, J = 7.3 Hz, 2H); $^{13}C\{^{1}H\}$ NMR (150 MHz, $CDCl_3$, mixture of rotamers) δ: 16.2, 22.1, 28.2, 32.6, 33.7, 46.3, 47.3, 56.9, 63.3, 65.5, 66.7, 117.6, 119.9, 125.0, 125.1, 127.0, 127.0, 127.7, 132.8, 141.3, 143.9, 156.2, 157.1; HRMS (FAB) *m/z*: $[M + H]^+$ calcd for $C_{27}H_{35}N_2O_5$, 467.2541; found: 467.2543.

(9*H*-Fluoren-9-yl)methyl allyl [(2*R*,5*S*,6*S*,*E*)-7-hydroxy-5-methylhept-3-ene-2,6-diyl]dicarbamate (15b). To a stirred solution of **13b** (403 mg, 1.05 mmol) in CH_2Cl_2 (2 mL) was added TFA/CH_2Cl_2 (1:3, 8 mL) at 0 °C. After being stirred for 30 min, the mixture was warmed to 20–25 °C and stirred for 1 h. The mixture was concentrated under reduced pressure to give the corresponding amine, which was used without further purification. To a stirred solution of the above amine in MeCN (4 mL) and H_2O (4 mL) were added Fmoc-OSu (462 mg, 1.37 mmol) in MeCN (2 mL) and DIEA (733 µL, 4.21 mmol) at 20–25 °C. The reaction mixture was stirred at 20–25 °C for 2 h. Then, water was added to the mixture at 20–25 °C. The whole was extracted with EtOAc and the extract was washed with saturated citric acid and brine, and dried over Na_2SO_4. The filtrate was concentrated under reduced pressure and the residue was purified by flash chromatography over silica gel (hexane/EtOAc = 1/1) to give compound **15b** (425 mg, 87%) as a white amorphous solid: $[\alpha]^{26}_{D}$ +17.8 (*c* 1.04, $CHCl_3$); IR (neat): 3314 (OH), 1688 (C=O); ^{1}H NMR (300 MHz, DMSO-d_6, 323 K) δ: 0.92 (d, J = 6.7 Hz, 3H), 1.11 (d, J = 6.8 Hz, 3H), 2.33–2.45 (m, 1H), 3.28–3.52 (m, 3H), 3.95–4.10 (m, 1H), 4.16–4.36 (m, 3H), 4.40–4.50 (m, 3H), 5.11–5.19 (m, 1H), 5.21–5.32 (m, 1H), 5.22–5.57 (m, 2H), 5.80–5.98 (m, 1H), 6.69 (d, J = 7.4 Hz, 1H), 6.92–7.10 (m, 1H), 7.28–7.36 (m, 2H), 7.37–7.45 (m, 2H), 7.69 (d, J = 7.4 Hz, 2H), 7.87 (d, J = 7.4 Hz, 2H); $^{13}C\{^{1}H\}$ NMR (150 MHz, $CDCl_3$, mixture of rotamers) δ: 16.7, 21.0, 28.4, 37.3, 47.3, 48.6, 48.9, 57.0, 63.8, 65.5, 66.7, 117.7, 120.0, 125.1, 127.0, 127.1, 127.7, 131.4, 132.7, 132.8, 132.9, 141.3, 143.9, 143.9, 155.5, 157.0; HRMS (ESI-TOF) *m/z*: $[M + Na]^+$ calcd for $C_{27}H_{32}N_2NaO_5$, 487.2203; found: 487.2203.

(2*S*,3*S*,6*R*)-2-({[(9*H*-Fluoren-9-yl)methoxy]carbonyl}amino)-6-{[(allyloxy)carbonyl]amino}-3-methylheptanoic acid (4a). To a stirred solution of **15a** (92.0 mg, 0.197 mmol) in MeCN (4.5 mL) and pH 7.0 phosphate buffer solution (1.5 mL) were added AZADOL (6.04 mg, 0.0394 mmol), $NaClO_2$ (44.5 mg, 0.394 mmol) and NaClO aqueous solution (120 µL, 0.197 mmol) at 0 °C. After being stirred for 1.5 h, the reaction mixture was diluted with 1 M HCl. The whole was extracted with EtOAc and the extract was washed with brine, and dried over Na_2SO_4. The filtrate was concentrated under reduced pressure and the residue was purified by preparative thin-layer chromatography over silica gel ($CHCl_3$/MeOH = 9/1) to give compound **4a** (71 mg, 75%) as a colorless amorphous solid: $[\alpha]^{25}_{D}$ +12.7

(c 0.613, $CHCl_3$); IR (neat): 1701 (C=O); ^{1}H NMR (300 MHz, DMSO-d_6, 323 K) δ: 0.87 (d, J = 7.0 Hz, 3H), 1.03 (d, J = 6.6 Hz, 3H), 1.16–1.55 (m, 4H), 1.79–1.89 (m, 1H), 3.37–3.49 (m, 1H), 3.82–3.92 (m, 1H), 4.18–4.30 (m, 3H), 4.41–4.47 (m, 2H), 5.10–5.18 (m, 1H), 5.21–5.30 (m, 1H), 5.81–5.97 (m, 1H), 6.89 (m, 1H), 7.25–7.46 (m, 5H), 7.71 (d, J = 7.0 Hz, 2H), 7.87 (d, J = 7.3 Hz, 2H), 12.44 (br s, 1H); ^{13}C{^{1}H} NMR (150 MHz, $CDCl_3$, mixture of rotamers) δ: 14.1, 15.8, 16.3, 21.5, 21.7, 22.7, 28.2, 29.7, 31.6, 34.2, 34.6, 35.4, 46.2, 46.8, 47.1, 59.3, 65.5, 66.0, 66.2, 66.9, 67.1, 117.6, 119.9, 125.2, 127.1, 127.7, 129.0, 129.4, 132.6, 132.8, 141.2, 143.8, 144.0, 156.2, 156.7, 176.6; HRMS (FAB) *m/z*: $[M + H]^+$ calcd for $C_{27}H_{33}N_2O_6$, 481.2334; found: 481.2331.

(2*S*,3*S*,6*R*,*E*)-2-({[(9*H*-Fluoren-9-yl)methoxy]carbonyl}amino)-6-{[(allyloxy)carbonyl]amino}-3-methylhept-4-enoic acid (4b). To a stirred solution of **15b** (139 mg, 0.299 mmol) in MeCN (6 mL) and pH 7.0 phosphate buffer solution (2 mL) were added AZADOL (9.14 mg, 0.0597 mmol), $NaClO_2$ (67.5 mg, 0.597 mmol) and NaClO aqueous solution (181 μL, 0.299 mmol) at 0 °C. After being stirred for 1.5 h, the reaction mixture was diluted with 1 M HCl. The whole was extracted with EtOAc and the extract was washed with brine, and dried over Na_2SO_4. The filtrate was concentrated under reduced pressure and the residue was purified by preparative thin-layer chromatography over silica gel ($CHCl_3$/MeOH = 9/1) to give compound **4b** (102 mg, 71%) as a colorless amorphous solid: $[\alpha]^{24}_D$ +18.2 (c 0.991, $CHCl_3$); IR (neat): 1708 (C=O); ^{1}H NMR (300 MHz, DMSO-d_6, 323 K) δ: 0.95 (d, J = 6.2 Hz, 3H), 1.10 (d, J = 6.8 Hz, 3H), 2.55–2.69 (m, 1H), 3.78–3.89 (m, 1H), 3.94–4.08 (m, 1H), 4.16–4.30 (m, 3H), 4.41–4.48 (m, 2H), 5.10–5.18 (m, 1H), 5.19–5.30 (m, 1H), 5.34–5.63 (m, 2H), 5.81–5.97 (m, 1H), 6.76–6.88 (m, 1H), 6.94–7.08 (m, 1H), 7.27–7.36 (m, 2H), 7.36–7.45 (m, 2H), 7.69 (d, J = 7.3 Hz, 2H), 7.87 (d, J = 7.2 Hz, 2H); ^{13}C{^{1}H} NMR (150 MHz, $CDCl_3$, mixture of rotamers) δ: 16.2, 20.9, 29.7, 38.6, 47.1, 48.7, 59.1, 65.6, 66.9, 67.3, 117.8, 119.9, 124.9, 125.2, 127.0, 127.7, 130.0, 132.7, 133.1, 141.2, 143.8, 144.0, 155.9, 156.7, 176.7; HRMS (FAB) *m/z*: $[M + Na]^+$ calcd for $C_{27}H_{30}N_2NaO_6$, 501.1996; found: 501.2003.

Determination of the Stereochemistry of Lysine Derivative 13b (Scheme S1)

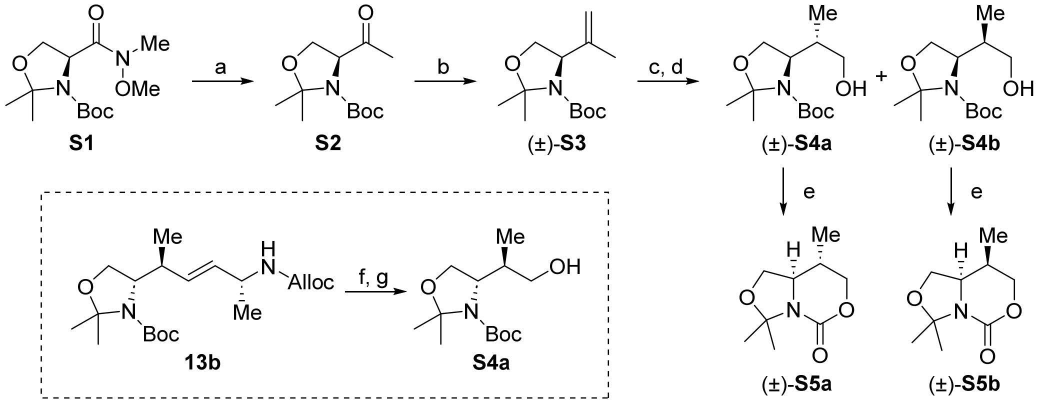

Scheme S1. Synthesis of an authentic sample for the determination of the stereochemistry of compound **13b**. *Reagents and conditions*: (a) MeLi, THF–Et_2O, –78 °C; (b) $Ph_3P\text{-}CH_3^+Br^-$, *t*-BuOK, 20–25 °C, 56% (2 steps); (c) $BH_3\cdot SMe_2$, THF, 0 °C; (d) H_2O_2, 20–25 °C, 12% ((±)-**S4a**) and 45% ((±)-**S4b**); (e) NaH, THF, 0 °C, then reflux, 67% ((±)-**S5a**) and 71% ((±)-**S5b**); (f) O_3, MeOH, –78 °C; (g) $NaBH_4$, –78 °C to 20–25 °C, 43%.

***tert*-Butyl 2,2-dimethyl-4-(prop-1-en-2-yl)oxazolidine-3-carboxylate ((±)-S3).** (±)-**S3** was synthesized by the identical procedure reported previously.[S1,S2] To a stirred solution of amide **S1** (1.00 g, 3.47 mmol) in dry THF (10 mL) under argon was added MeLi (1.09 M solution in Et_2O; 6.37 mL, 6.93 mmol) at –78 °C. After being stirred for 2 h, the reaction mixture was diluted with aqueous saturated NH_4Cl. The whole was extracted with EtOAc and the extract was washed with brine, and dried over Na_2SO_4. The filtrate was concentrated under reduced pressure to give ketone **S2**, which was used without further purification. To a stirred solution of methyltriphenylphosphonium bromide (3.72 g, 10.4 mmol) in dry THF (10 mL) under argon was added *t*-BuOK (1.17 g, 10.4 mmol) at 0 °C. The reaction mixture was warmed to 20–25 °C and stirred for 10 min. To this mixture was added a solution of the ketone **S2** in dry THF (5 mL) at 20–25 °C. After being stirred for 10 min, the reaction mixture was diluted with brine. The whole was extracted with EtOAc and the extract was dried over Na_2SO_4. The filtrate was concentrated under reduced pressure and the residue was purified by flash chromatography over silica gel (hexane/EtOAc = 7/1) to give compound (±)-**S3** (472 mg, 56%) as a colorless oil of racemic mixture. The spectral data were in good agreement with those previous reported.[S2]

***tert*-Butyl (*S*/*R*)-4-[(*R*/*S*)-1-hydroxypropan-2-yl]-2,2-dimethyloxazolidine-3-carboxylate** ((±)-**S4**). To a stirred solution of (±)-**S3** (300 mg, 1.24 mmol) in dry THF (9 mL) under argon was added $BH_3\cdot SMe_2$ (2.0 M in THF solution; 2.47 mL, 4.97 mmol) at 0 °C. After the mixture was stirred for 4

h, aqueous solutions of 30% H_2O_2 and saturated NH_4Cl were added to the mixture, which was warmed to 20–25 °C and stirred overnight. The whole was extracted with EtOAc and the extract was washed with brine, and dried over Na_2SO_4. The filtrate was concentrated under reduced pressure and the residue was purified by flash chromatography over silica gel (hexane/EtOAc = 4/1) to give compound (±)-**S4a** (37.0 mg, 12%) as a white amorphous solid and compound (±)-**S4b** (144 mg, 45%) as a colorless oil.

(±)-**S4a**: IR (neat): 3286 (OH), 1690 (C=O); ^{1}H NMR (600 MHz, $CDCl_3$, mixture of rotamers) δ: 1.04 (d, *J* = 6.8 Hz, 3H), 1.48–1.52 (m, 15H), 1.62–1.69 (m, 1H), 3.35–3.43 (m, 1H), 3.59–3.65 (m, 1H), 3.79–3.86 (m, 2H), 3.92 (dd, *J* = 8.9, 5.3 Hz, 2H); $^{13}C\{^1H\}$ NMR (150 MHz, $CDCl_3$, mixture of rotamers) δ: 14.8, 24.4, 27.6, 28.4, 38.6, 59.2, 63.4, 66.3, 81.2, 93.5, 154.3; HRMS (ESI-TOF) *m/z*: $[M + Na]^+$ calcd for $C_{13}H_{25}NNaO_4$, 282.1676; found: 282.1676.

(±)-**S4b**: IR (neat): 3486 (OH), 1695 (C=O); ^{1}H NMR (500 MHz, $CDCl_3$, mixture of rotamers) δ: 0.85 (d, *J* = 6.8 Hz, 3H), 1.48 (s, 12H), 1.58 (s, 3H), 1.85–1.94 (m, 1H), 3.23–3.31 (m, 1H), 3.42–3.50 (m, 1H), 3.79–3.84 (m, 1H), 4.00–4.07 (m, 1H), 4.12–4.19 (m, 1H), 4.24–4.30 (m, 1H); $^{13}C\{^1H\}$ NMR (150 MHz, $CDCl_3$, mixture of rotamers) δ: 11.3, 23.9, 26.1, 28.2, 40.1, 56.7, 64.6, 66.4, 80.8, 94.3, 154.5; HRMS (ESI-TOF) *m/z*: $[M + Na]^+$ calcd for $C_{13}H_{25}NNaO_4$, 282.1676; found: 282.1676.

(8*R/S*,8a*S/R*)-3,3,8-Trimethyltetrahydro-3*H*,5*H*-oxazolo[3,4-*c*][1,3]oxazin-5-one ((±)-**S5a**). To a stirred suspension of NaH (34.0 mg, 0.848 mmol) in dry THF (0.5 mL) under argon was added a solution of (±)-**S4a** (44.0 mg, 0.170 mmol) in dry THF (1 mL) at 0 °C. The reaction mixture was refluxed for 15 min. Then, aqueous saturated NH_4Cl was added to the mixture at 20–25 °C. The whole was extracted with EtOAc and the extract was washed with aqueous saturated $NaHCO_3$ and brine, and dried over Na_2SO_4. The filtrate was concentrated under reduced pressure and the residue was purified by flash chromatography over silicagel (hexane/EtOAc = 1/1) to give compound (±)-**S5a** (21.1 mg, 67%) as colorless block crystals: mp 103–105 °C; IR (neat): 1687 (C=O); ^{1}H NMR (500 MHz, $CDCl_3$) δ: 0.97 (d, *J* = 6.6 Hz, 3H), 1.57 (s, 3H), 1.61 (s, 3H), 1.81–1.92 (m, 1H), 3.39 (ddd, *J* = 10.0, 10.0, 5.6 Hz, 1H), 3.54 (dd, *J* = 9.9, 8.5 Hz, 1H), 3.83 (dd, *J* = 11.3, 11.3 Hz, 1H), 4.16–4.24 (m, 2H); $^{13}C\{^1H\}$ NMR (150 MHz, $CDCl_3$) δ: 12.6, 23.7, 25.9, 31.2, 60.5, 68.7, 72.2, 95.5, 149.5; HRMS (ESI-TOF) *m/z*: $[M + Na]^+$ calcd for $C_9H_{15}NNaO_3$, 208.0944; found: 208.0944.

(8*R/S*,8a*R/S*)-3,3,8-Trimethyltetrahydro-3*H*,5*H*-oxazolo[3,4-*c*][1,3]oxazin-5-one ((±)-**S5b**). To a stirred suspension of NaH (110 mg, 2.76 mmol) in dry THF (2 mL) under argon was added a solution of (±)-**S4b** (143 mg, 0.552 mmol) in dry THF (4 mL) at 0 °C. The reaction mixture was refluxed for 15 min. Then, aqueous saturated NH_4Cl was added to the mixture at 20–25 °C. The whole was

extracted with EtOAc and the extract was washed with aqueous saturated $NaHCO_3$ and brine, and dried over Na_2SO_4. The filtrate was concentrated under reduced pressure and the residue was purified by flash chromatography over silica gel (hexane/EtOAc = 1/1) to give compound (±)-**S5b** (72.7 mg, 71%) as a colorless oil: IR (neat): 1688 (C=O); ^{1}H NMR (500 MHz, $CDCl_3$) δ: 1.06 (d, J = 7.1 Hz, 3H), 1.56 (s, 3H), 1.62 (s, 3H), 2.15–2.23 (m, 1H), 3.64 (dd, J = 10.1, 8.7 Hz, 1H), 3.89–3.95 (m, 1H), 4.04 (dd, J = 8.6, 5.8 Hz, 1H), 4.11 (dd, J = 11.0, 1.7 Hz, 1H), 4.28 (dd, J = 11.0, 2.6 Hz, 1H); ^{13}C{^{1}H} NMR (150 MHz, $CDCl_3$) δ: 10.4, 23.4, 25.9, 26.1, 57.7, 65.7, 72.9, 94.6, 149.2; HRMS (ESI-TOF) *m/z*: $[M + Na]^+$ calcd for $C_9H_{15}NNaO_3$, 208.0944; found: 208.0944.

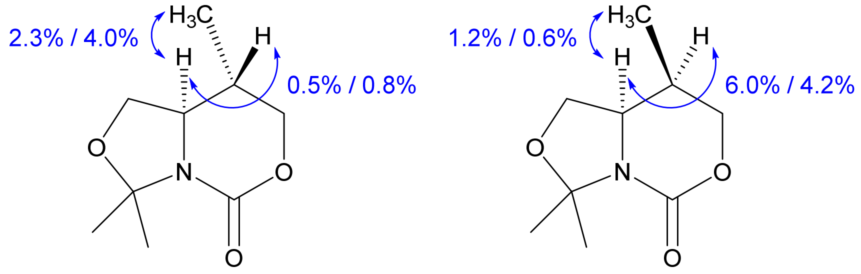

(*S*,*R*)-**S5a** (*R*,*R*)-**S5b**

Figure S2. NOE correlations for the determination of the stereochemistries in CbA Lys derivatives.

***tert*-Butyl (*S*)-4-[(*R*)-1-hydroxypropan-2-yl]-2,2-dimethyloxazolidine-3-carboxylate (S4a).** O_3 gas was bubbled into a stirred solution of **13b** (49.5 mg, 0.129 mmol) in MeOH (5 mL) at −78 °C for 20 min. O_2 was then bubbled through the solution for 10 min to remove excess O_3. To the reaction mixture was added $NaBH_4$ (49.0 mg, 1.29 mmol). The reaction mixture was warmed to 20–25 °C and stirred for 2 h. The reaction was quenched with saturated citric acid at 0 °C. The whole was extracted with EtOAc and the extract was washed with aqueous saturated $NaHCO_3$ and brine, and dried over Na_2SO_4. The filtrate was concentrated under reduced pressure and the residue was purified by flash chromatography over silica gel (hexane/EtOAc = 2/1) to give compound **S4a** (14.5 mg, 43%) as a white amorphous solid: $[\alpha]^{23}_D$ –5.58 (*c* 0.63, $CHCl_3$); IR (neat): 3253 (OH), 1688 (C=O); ^{1}H NMR (600 MHz, $CDCl_3$, mixture of rotamers) δ: 1.03 (d, J = 6.8 Hz, 3H), 1.47–1.52 (m, 15H), 1.61–1.70 (m, 1H), 3.34–3.41 (m, 1H), 3.58–3.63 (m, 1H), 3.74–3.85 (m, 2H), 3.91 (dd, J = 8.9, 5.3 Hz, 2H); ^{13}C{^{1}H} NMR (150 MHz, $CDCl_3$, mixture of rotamers) δ: 14.8, 24.4, 27.5, 28.3, 38.6, 59.1, 63.3, 66.2, 81.2, 93.5, 154.3; HRMS (ESI-TOF) *m/z*: $[M + Na]^+$ calcd for $C_{13}H_{25}NNaO_4$, 282.1676; found: 282.1677.

General Procedure for Solid-Phase Peptide Synthesis.

Loading of an amino acid on the solid support. A solution of Fmoc amino acid (0.198 mmol) and DIEA (138 μL, 0.792 mmol) in dry CH_2Cl_2 (2.0 mL), was reacted with (2-Cl)Trt chloride resin (204 mg, 0.326 mmol). The reaction was continued for 2 h at 20–25 °C.

Deprotection of Fmoc group. The Fmoc-protected peptidyl resin was treated with 20% piperidine/DMF for 20 min.

Coupling reaction using HATU/DIEA. DIEA (104 μL, 0.600 mmol) was added to a solution of Fmoc amino acid (0.30 mmol) and HATU (110 mg, 0.290 mmol) in DMF. The whole was poured into the peptidyl resin (0.10 mmol), and the reaction was continued for 1.5 h at 40 °C. For the coupling of N-terminal Me_2Val-D-Hva-OH, an excess amount of DIEA (157 μL, 0.900 mmol) was used.

Coupling reaction using DIC/HOBt. DIC (46 μL, 0.30 mmol) was added to a solution of Fmoc amino acid (0.30 mmol) and HOBt·H_2O (46 mg, 0.30 mmol) in DMF. The whole was poured into the peptidyl resin (0.100 mmol), and the reaction was continued for 1.5 h at 40 °C.

N-Methylation on solid support.[S3] 2,4,6-Collidine (132 μL, 1.00 mmol) was added to a solution of NsCl (111 mg, 0.500 mmol) in NMP. The whole was poured into the peptidyl resin (0.100 mmol), and the reaction was continued for 15 min at 20–25 °C. After removal of the reagent solution, a solution of MeOH (20 μL, 0.50 mmol) and Ph_3P (131 mg, 0.500 mmol) in dry THF was added into the peptidyl resin. DEAD (228 μL, 0.500 mmol) was added dropwise, and the reaction was continued for 30 min at 20–25 °C. This reaction was repeated twice. To a suspension of the peptidyl resin in NMP, DBU (75 μL, 0.50 mmol) and 2-mercaptoethanol (70 μL, 1.0 mmol) were added, and the reaction was continued for 5 min. This deprotection process was repeated twice.

Deprotection of Alloc group. To the peptidyl resin (0.100 mmol) were added $PhSiH_3$ (247 μL, 2.00 mmol) and $Pd(PPh_3)_4$ (23 mg, 0.020 mmol) in dry CH_2Cl_2, and the reaction was continued for 10 min.

Cleavage from the resin. The peptidyl resin was treated with 1,1,1,3,3,3-hexafluoropropan-2-ol (HFIP)/CH_2Cl_2 (3:7) for 2 h at 20–25 °C. After filtration of the residual resin, the filtrate was concentrated under reduced pressure to give a crude peptide, which was used for the next step without further purification.

Synthesis of Coibamide A Derivatives with a Modified Lysine (3a,b).

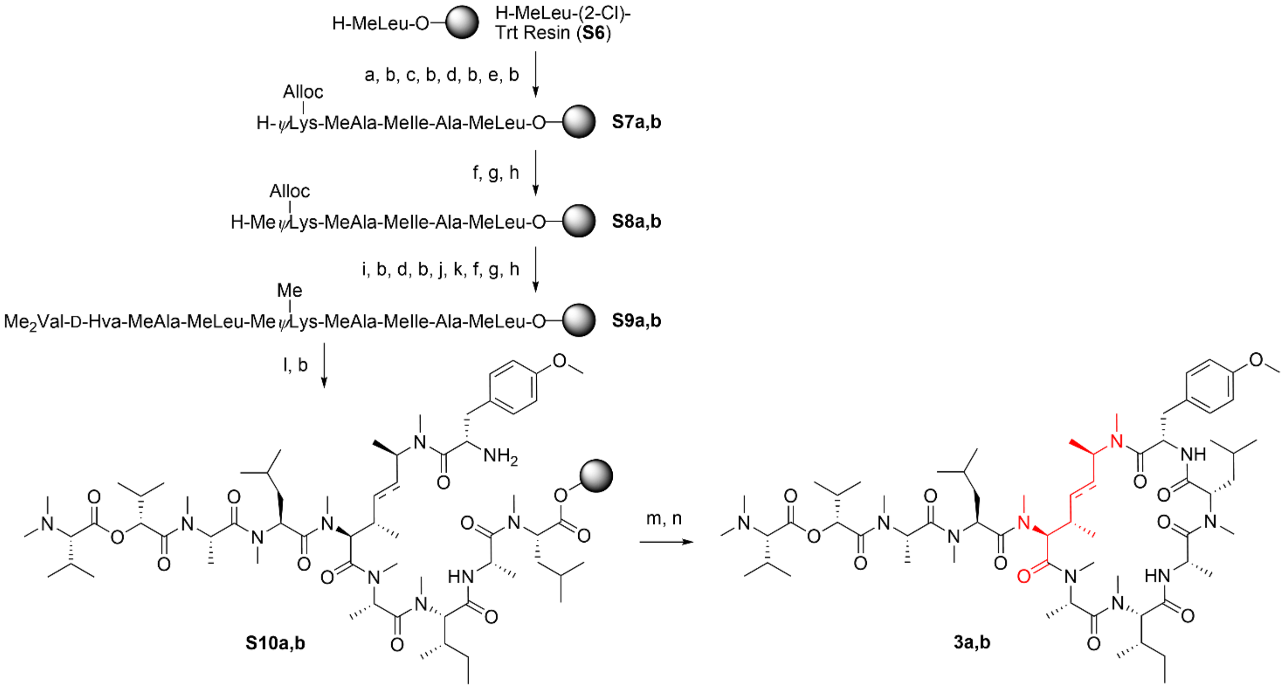

Scheme S2. Synthesis of coibamide A derivatives with a modified lysine (**3a,b**). *Reagents and conditions*: (a) Fmoc-Ala-OH·H_2O, HATU, DIEA, DMF, 40 °C; (b) 20% piperidine/DMF, 20–25 °C; (c) Fmoc-MeIle-OH, HOBt·H_2O, DIC, DMF, 40 °C; (d) Fmoc-MeAla-OH, HATU, DIEA, DMF, 40 °C; (e) Fmoc- ψLys(Alloc)-OH, HATU, DIEA, DMF, 40 °C; (f) NsCl, 2,4,6-collidine, NMP, 20–25 °C; (g) Ph_3P, DEAD, MeOH, THF, 20–25 °C; (h) 2-mercaptoethanol, DBU, NMP, 20–25 °C; (i) Fmoc-MeLeu-OH, HATU, DIEA, DMF, 40 °C; (j) Me_2Val-D-Hva-OH·HCl, HATU, DIEA, NMP, 40 °C; (k) $Pd(PPh_3)_4$, $PhSiH_3$, CH_2Cl_2, 20–25 °C; (l) Fmoc-Tyr(Me)-OH, HATU, DIEA, NMP, 40 °C; (m) 30% HFIP/CH_2Cl_2, 20–25 °C; (n) EDCI·HCl, HOAt, DIEA, DMF, 0 °C to 20–25 °C.

Synthesis of compound 3a. The linear peptides were constructed by solid-phase peptide synthesis on peptidyl resin **S6** (0.490 mmol/g, 102 mg, 0.0500 mmol). After the cleavage from the resin **S10a** as described above, EDCI·HCl (96 mg, 0.50 mmol) was added to a solution of crude linear peptide, HOAt (68 mg, 0.50 mmol), and DIEA (348 μL, 2.00 mmol) in dry DMF (50 mL) at 0 °C. The reaction mixture was allowed to warm up to 20–25 °C and the stirring was continued for 18 h. The reaction mixture was concentrated *in vacuo* and the residue was purified by RP-HPLC to give compound **3a** (3.7 mg, 3% from resin) as a white powder: ^{1}H NMR (600 MHz, $CDCl_3$, mixture of rotamers) δ: 0.43–2.29 (m, 58H), 2.47–3.10 (m, 29H), 3.19–3.30 (m, 3H), 3.69–3.82 (m, 3H), 3.82–3.92 (m, 1H), 3.92–4.06 (m, 1H), 4.41–5.66 (m, 9H), 6.58–6.83 (m, 2H), 6.83–6.94 (m, 1H), 6.94–7.18 (m, 2H), 8.67–8.98 (m, 1H); ^{13}C{^{1}H} NMR (150 MHz, $CDCl_3$, mixture of rotamers) δ: 10.1, 12.0, 14.0, 14.1, 14.4, 14.7 15.4, 15.5, 16.1, 16.8, 16.9, 18.0, 18.7, 18.8, 19.1, 19.2, 20.7, 21.3, 21.5, 22.6, 23.3, 23.47, 23.52, 24.5, 24.6,

25.0, 25.2, 25.5, 26.8, 26.9, 28.2, 28.25, 28.28, 29.1, 29.6, 29.89, 29.93, 30.1, 30.4, 31.4, 31.6, 32.7, 35.6, 37.2, 37.4, 45.2, 46.3, 46.9, 49.5, 49.7, 50.2, 50.4, 51.2, 51.3, 55.1, 55.2, 56.5, 56.7, 65.7, 70.8, 113.6, 115.5, 117.5, 127.5, 130.9, 158.7, 162.2, 162.5, 167.4, 167.7, 168.1, 169.5, 170.6, 170.7, 171.5, 172.6, 173.6, 173.7; HRMS (FAB) *m/z*: $[M + H]^+$ calcd for $C_{64}H_{111}N_{10}O_{12}$, 1211.8378; found: 1211.8407.

Synthesis of compound 3b. According to the procedure described for the preparation of **3a**, peptidyl resin **S6** (0.650 mmol/g, 77 mg, 0.050 mmol) was converted into **3b** (1.2 mg, 1% from resin) as a white powder: ^{1}H NMR (600 MHz, $CDCl_3$, mixture of rotamers) δ: 0.75–2.37 (m, 54H), 2.37–2.63 (m, 4H), 2.63–3.30 (m, 29H), 3.65–3.99 (m, 5H), 4.80–4.91 (m, 1H), 4.91–5.02 (m, 2H), 5.02–5.28 (m, 3H), 5.28–5.43 (m, 1H), 5.43–5.52 (m, 2H), 5.52–5.67 (m, 1H), 6.59–6.95 (m, 3H), 6.95–7.17 (m, 3H); ^{13}C{^{1}H} NMR (150 MHz, $CDCl_3$, mixture of rotamers) δ: 11.7, 14.1, 14.4, 14.7, 16.0, 17.3, 17.6, 18.6, 18.8, 18.9, 19.1, 19.3, 21.0, 22.1, 22.7, 23.2, 24.6, 24.8, 25.0, 25.3, 28.2, 29.0, 29.3, 29.7, 30.0, 30.1, 30.6, 31.9, 32.6, 37.5, 38.1, 46.8, 50.0, 51.1, 51.5, 55.3, 65.1, 71.0, 113.8, 113.9, 127.6, 130.2, 130.4, 158.5, 167.6, 168.0, 168.7, 169.6, 170.6, 172.2; HRMS (FAB) *m/z*: $[M + H]^+$ calcd for $C_{64}H_{109}N_{10}O_{12}$, 1209.8221; found: 1209.8239.

Synthesis of Coibamide A Derivative with a D-MeAla-MeDab Linkage (16a)

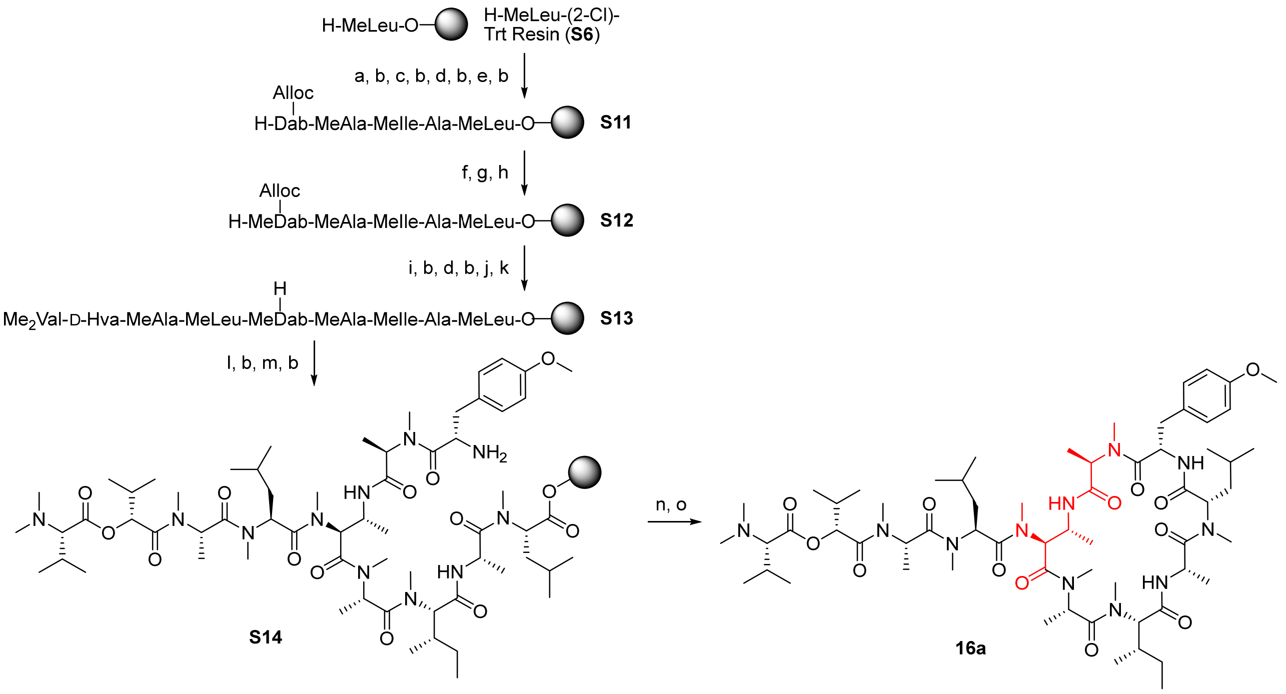

Scheme S3. Synthesis of coibamide A derivative with a D-MeAla-MeDab linkage (**16a**). *Reagents and conditions*: (a) Fmoc-Ala-OH·H_2O, HATU, DIEA, DMF, 40 °C; (b) 20% piperidine/DMF, 20–25 °C; (c) Fmoc-MeIle-OH, HOBt·H_2O, DIC, DMF, 40 °C; (d) Fmoc-MeAla-OH, HATU, DIEA, DMF, 40 °C; (e) Fmoc-Dab(Alloc)-OH, HATU, DIEA, DMF, 40 °C; (f) NsCl, 2,4,6-collidine, NMP, 20–25 °C; (g) Ph_3P, DEAD, MeOH, THF, 20–25 °C; (h) 2-mercaptoethanol, DBU, NMP, 20–25 °C; (i) Fmoc-MeLeu-OH, HATU, DIEA, DMF, 40 °C; (j) Me_2Val-D-Hva-OH, HATU, DIEA, NMP, 40 °C; (k) $Pd(PPh_3)_4$, $PhSiH_3$, CH_2Cl_2, 20–25 °C; (l) Fmoc-D-MeAla-OH, HOBt·H_2O, DIC, DMF, 40 °C; (m) Fmoc-Tyr(Me)-OH, HATU, DIEA, NMP, 40 °C; (n) 30% HFIP/CH_2Cl_2, 20–25 °C; (o) EDCI·HCl, HOAt, DIEA, DMF, 0 °C to 20–25 °C.

Synthesis of compound 16a. The linear peptides were constructed by solid-phase peptide synthesis on peptidyl resin **S6** (0.490 mmol/g, 206 mg, 0.100 mmol). After the cleavage from the resin **S14** as described above, EDCI·HCl (192 mg, 1.00 mmol) was added to a solution of crude linear peptide, HOAt (136 mg, 1.00 mmol), and DIEA (697 μL, 4.00 mmol) in dry DMF (100 mL) at 0 °C. The reaction mixture was allowed to warm up to 20–25 °C and the stirring was continued for 18 h. The reaction mixture was concentrated in vacuo and the residue was purified by RP-HPLC to give compound **16a** (10.4 mg, 8% from resin) as a white powder: ^{1}H NMR (600 MHz, $CDCl_3$, mixture of rotamers) δ: 0.53–1.72 (m, 48H), 1.90–2.15 (m, 2H), 2.15–2.36 (m, 3H), 2.36–2.59 (m, 4H), 2.59–2.82 (m, 6H), 2.82–3.30 (m, 23H), 3.52–3.89 (m, 5H), 4.15–4.24 (m, 0.5H), 4.40–4.68 (m, 1.5H), 4.68–4.91 (m, 1.5H), 4.91–5.15 (m, 2.5H), 5.34–5.68 (m, 3H), 5.68–6.15 (m, 1H), 6.62–6.88 (m, 2.5H),

6.88–7.01 (m, 0.5H), 7.01–7.18 (m, 2H), 7.30–7.43 (m, 1H); $^{13}C\{^{1}H\}$ NMR (150 MHz, $CDCl_3$, mixture of rotamers) δ: 11.7, 11.8, 12.6, 12.8, 16.0, 17.3, 17.4, 17.5, 18.0, 18.5, 18.59, 18.64, 18.9, 19.0, 20.8, 22.2, 22.4, 22.7, 23.1, 23.4, 24.3, 24.9, 25.0, 25.2, 25.5, 28.2, 28.8, 29.3, 29.4, 29.5, 29.7, 29.8, 30.0, 30.1, 30.17, 30.21, 30.3, 30.9, 32.1, 34.0, 35.9, 36.9, 37.4, 37.9, 38.1, 39.0, 44.3, 44.4, 46.5, 47.1, 51.0, 51.4, 51.7, 51.8, 51.9, 52.3, 52.7, 52.9, 53.0, 55.30, 55.33, 56.0, 58.5, 58.75, 58.80, 58.86, 58.91, 64.6, 64.7, 64.8, 68.4, 68.6, 68.88, 68.93, 71.3, 71.4, 113.9, 114.3, 126.9, 127.8, 130.1, 130.2, 158.8, 159.1, 167.1, 167.7, 167.8, 169.0, 169.1, 169.6, 169.8, 170.0, 170.1, 170.3, 170.4, 170.8, 171.7, 171.9, 172.6, 173.0; HRMS (FAB) *m/z*: $[M + Na]^+$ calcd for $C_{63}H_{107}N_{11}NaO_{13}$, 1248.7942; found: 1248.7963.

Synthesis of compound 16b. According to the procedure described for the preparation of **16a**, peptidyl resin **S6** (0.490 mmol/g, 206 mg, 0.100 mmol) was converted into **16b** (15.0 mg, 11% from resin) as a white powder: ^{1}H NMR (600 MHz, $CDCl_3$, mixture of rotamers) δ: 0.71–1.70 (m, 48H), 1.87–2.14 (m, 1H), 2.14–2.32 (m, 2H), 2.41–2.55 (m, 3H), 2.67–2.76 (m, 3H), 2.76–3.26 (m, 23H), 3.26–3.39 (m, 6H), 3.47–3.92 (m, 9H), 4.19–4.60 (m, 2H), 4.70–4.94 (m, 2H), 4.94–5.10 (m, 2H), 5.31–5.47 (m, 1H), 5.67–5.89 (m, 2H), 6.00–6.22 (m, 1H), 6.61–6.73 (m, 0.5H), 6.73–6.89 (m, 2.5H), 6.89–6.97 (m, 0.5H), 6.97–7.12 (m, 2H), 7.12–7.23 (m, 0.5H); $^{13}C\{^{1}H\}$ NMR (150 MHz, $CDCl_3$, mixture of rotamers) δ: 11.7, 11.8, 12.6, 12.8, 16.0, 17.3, 17.4, 17.5, 18.0, 18.5, 18.59, 18.64, 18.9, 19.0, 19.4, 20.8, 22.2, 22.4, 22.7, 23.1, 23.4, 24.3, 24.9, 25.0, 25.2, 25.5, 28.2, 28.8, 29.3, 29.4, 29.5, 29.7, 29.8, 30.0, 30.1, 30.17, 30.21, 30.3, 30.9, 32.1, 34.0, 35.9, 36.9, 37.4, 37.87, 37.95, 38.1, 39.0, 44.3, 44.4, 46.5, 47.1, 51.0, 51.4, 51.7, 51.7, 51.8, 51.9, 52.3, 52.7, 52.9, 53.0, 55.29, 55.33, 56.0, 58.5, 58.9, 64.6, 64.7, 64.8, 68.4, 68.6, 68.88, 68.93, 71.3, 71.4, 113.9, 114.3, 126.9, 127.8, 130.1, 130.2, 158.8, 159.1, 167.1, 167.7, 168.8, 169.0, 169.1, 169.6, 169.8, 170.0, 170.1, 170.3, 170.4, 170.8, 171.7, 171.9, 172.6, 173.0; HRMS (FAB) *m/z*: $[M + H]^+$ calcd for $C_{65}H_{112}N_{11}O_{15}$, 1286.8334; found: 1286.8353.

Synthesis of compound 16c. According to the procedure described for the preparation of **16a**, peptidyl resin **S3** (0.490 mmol/g, 206 mg, 0.100 mmol) was converted into **16c** (10.5 mg, 7% from resin) as a white powder: ^{1}H NMR (600 MHz, $CDCl_3$, mixture of rotamers) δ: 0.74–1.78 (m, 48H), 1.88–2.15 (m, 1H), 2.15–2.39 (m, 2H), 2.39–2.61 (m, 3H), 2.61–2.81 (m, 3H), 2.81–3.27 (m, 23H), 3.27–3.43 (m, 6H), 3.43–3.57 (m, 1H), 3.57–3.93 (m, 5H), 4.22–4.64 (m, 2H), 4.69–4.79 (m, 1H), 4.79–4.94 (m, 1H), 4.94–5.12 (m, 2H), 5.30–5.46 (m, 1H), 5.59–5.95 (m, 2H), 6.00–6.23 (m, 1H), 6.68–6.74 (m, 1H), 6.88–6.97 (m, 1H), 7.19–7.62 (m, 9H); $^{13}C\{^{1}H\}$ NMR (150 MHz, $CDCl_3$, mixture of rotamers) δ: 11.6, 11.8, 12.5, 12.8, 14.1, 16.0, 17.1, 17.2, 17.5, 17.9, 18.5, 18.6, 18.8, 19.0, 19.1, 19.3, 20.7, 22.1, 22.2, 22.3, 22.4, 22.6, 23.1, 23.3, 24.2, 24.9, 25.0, 25.2, 25.5, 28.1, 28.7, 29.26, 29.35, 29.7, 29.8, 29.9,

30.05, 30.1, 30.17, 30.24, 30.4, 30.8, 31.9, 32.1, 34.0, 36.2, 37.2, 37.3, 37.86, 37.34, 38.92, 44.2, 44.3, 46.4, 47.1, 51.0, 51.8, 52.0, 52.6, 53.0, 53.1, 56.0, 57.6, 58.5, 58.7, 58.79, 58.83, 58.9, 64.5, 64.7, 64.8, 68.3, 68.5, 68.80, 68.84, 71.0, 71.1, 126.90, 126.93, 127.2, 127.4, 127.5, 128.8, 128.9, 129.49, 129.55, 134.1, 134.8, 140.2, 140.3, 140.4, 140.6, 162.1, 162.3, 167.1, 167.7, 168.1, 168.2, 168.4, 168.7, 168.8, 168.9, 169.4, 169.6, 169.97, 170.05, 170.3, 170.5, 171.0, 171.7, 171.8, 172.5, 172.9; HRMS (FAB) *m/z*: $[M + H]^+$ calcd for $C_{70}H_{114}N_{11}O_{14}$, 1332.8541; found: 1332.8560.

Synthesis of compound 17a. According to the procedure described for the preparation of **16a**, peptidyl resin **S6** (0.590 mmol/g, 171 mg, 0.100 mmol) was converted into **17a** (7.0 mg, 5% from resin) as a white powder: ^{1}H NMR (600 MHz, $CDCl_3$, mixture of rotamers) δ: 0.62–2.30 (m, 52H), 2.43–2.54 (m, 3H), 2.54–3.16 (m, 28H), 3.58–3.92 (m, 6H), 3.92–4.26 (m, 1H), 4.50–4.64 (m, 1H), 4.82–5.15 (m, 4H), 5.15–5.44 (m, 2H), 5.44–5.74 (m, 1H), 6.35–6.51 (m, 0.5H), 6.57–6.75 (m, 1.5H), 6.75–6.91 (m, 2H), 7.03–7.15 (m, 2H), 7.53–7.77 (m, 1H); ^{13}C{^{1}H} NMR (150 MHz, $CDCl_3$, mixture of rotamers) δ: 11.6, 11.8, 12.6, 12.9, 14.5, 14.8, 15.6, 16.0, 16.9, 17.1, 17.2, 18.7, 18.8, 18.9, 19.1, 19.22, 19.25, 19.3, 21.3, 22.1, 22.5, 22.7, 22.8, 24.8, 25.0, 25.1, 25.2, 28.2, 29.0, 29.1, 29.2, 29.4, 29.5, 30.0, 30.1, 30.2, 30.3, 30.5, 32.3, 33.2, 33.6, 35.8, 36.8, 37.1, 37.5, 37.7, 39.0, 46.1, 46.4, 48.3, 49.0, 50.1, 50.3, 50.8, 51.2, 51.4, 51.9, 52.4, 55.3, 55.4, 58.2, 58.9, 64.6, 65.1, 70.9, 113.9, 114.3, 126.7, 127.9, 130.1, 130.3, 158.8, 159.2, 167.80, 167.85, 168.0, 168.1, 168.5, 170.0, 170.7, 171.0, 171.4, 172.0, 172.1, 172.7, 173.5, 173.9; HRMS (ESI-TOF) *m/z*: $[M + Na]^+$ calcd for $C_{62}H_{105}N_{11}NaO_{13}$, 1234.7786; found: 1234.7778.

Synthesis of compound 17b. According to the procedure described for the preparation of **16a**, peptidyl resin **S6** (0.590 mmol/g, 171 mg, 0.100 mmol) was converted into **17b** (3.1 mg, 2% from resin) as a white powder: ^{1}H NMR (600 MHz, $CDCl_3$, mixture of rotamers) δ: 0.82–1.46 (m, 48H), 1.90–1.97 (m, 1H), 2.13–2.27 (m, 3H), 2.44–2.49 (m, 3H), 2.66–2.70 (m, 3H), 2.75–3.07 (m, 26H), 3.46–3.57 (m, 1H), 3.73–3.80 (m, 3H), 3.83–3.88 (m, 1H), 3.83–3.88 (m, 1H), 4.13–4.20 (m, 1H), 4.42–4.49 (m, 1H), 4.93–5.01 (m, 2H), 5.07–5.14 (m, 1H), 5.27–5.34 (m, 1H), 5.39–5.51 (m, 2H), 5.55–5.61 (m, 1H), 5.63–5.69 (m, 1H), 5.73–5.79 (m, 1H), 6.51–6.58 (m, 1H), 6.77–6.84 (m, 2H), 7.02–7.16 (m, 2H), 8.20–8.28 (m, 1H); HRMS (ESI-TOF) *m/z*: $[M + Na]^+$ calcd for $C_{63}H_{107}N_{11}NaO_{13}$, 1248.7942; found: 1248.7942.

Viability Assay Using A549 Cells[S4]

A549 cells were cultured in Dulbecco's modified Eagle's medium (DMEM; Sigma) supplemented with

10% (v/v) fetal bovine serum at 37 °C in a 5% CO_2-incubator. Viability assays using A549 cells were performed in 96-well plates (BD Falcon). A549 cells were seeded at 1000 cells/well in 90 μL of culture media, respectively, and were cultured for 24 h. Chemical compounds in DMSO were diluted 50-fold with the culture medium in advance. 30 μL of the chemical diluents were added. The final volume of DMSO in the medium was equal to 0.5% (v/v). The cells under chemical treatment were incubated for a further 72 h. The wells in the plates were washed twice with the cultured medium without phenol-red. After 1-hour incubation with 100 μL of the medium, the cell culture in each well was supplemented with 20 μL of the MTS reagent (Promega), followed by incubation for additional 40 min. Absorbance at 490 nm of each well was measured using a Wallac 1420 ARVO SX multilabel counter (Perkin Elmer). Three experiments were performed per condition and the averages of inhibition rates in each condition were evaluated to determine IC_{50} values using the GraphPad Prism software.

Viability Assays Using U87 MG Cells, HCT116 Cells and normal human dermal fibroblasts.
Human U87-MG glioblastoma, HCT116 colon cancer cells and normal human dermal fibroblasts were from American Type Culture Collection (ATCC, Manasses, VA). U87-MG cells were cultured in Minimum Essential Medium (MEM) with Earl's salts and L-glutamine (Corning Life Sciences), supplemented with 10% fetal bovine serum (FBS; Hyclone, Logan, UT) and 100 U/mL penicillin and 100 mg/mL streptomycin (1% penicillin/streptomycin). HCT116 cells were cultured in McCoy's 5A medium supplemented with 10% FBS and 1% penicillin/streptomycin. Human dermal fibroblasts were cultured in Fibroblast Basal Medium (#PCS-201-030) supplemented with Fibroblast Growth Kit (#PCS-201-041) from ATCC. All cells were grown under standard conditions and maintained at 37 ºC in an atmosphere of 5% CO_2. For analysis of biological activity, cells were seeded at 3,000 cells/well into 96-well plates and allowed to attach overnight. On the day of the experiment, all cells were treated at the same time with the test compound or vehicle (0.1% DMSO) and plates returned to the cell culture incubator for 72 h. The viability of cells was measured at 72 h using a CellTiter-Glo® Luminescent Cell Viability Assay (Promega Corp) and luminescent signals measured using a multi-mode microplate reader (Biotek Synergy HT). Concentration-response relationships were analyzed using GraphPad Prism Software version 9.5.1 (GraphPad Software, Inc., San Diego, CA), and CC_{50} values derived using nonlinear regression analysis fit to a logistic equation.

***Gaussia* Luciferase (GLuc) Secretory Assay**
The generation of U87-MG cells expressing the secreted reporter *Gaussia* luciferase (GLuc) has been described previously.[S5,S6] U87-MG-GLuc cells were cultured in Minimum Essential Medium (MEM) with Earl's salts and L-glutamine (Corning Life Sciences), supplemented with 10% fetal bovine serum

(FBS; Hyclone, Logan, UT) and 100 U/mL penicillin and 100 mg/mL streptomycin (1% penicillin/streptomycin). All cells were grown under standard conditions and maintained at 37 ºC in an atmosphere of 5% CO_2. For analysis of biological activity, cells were seeded at 3,000 cells/well into 96-well plates and allowed to attach overnight. On the day of the experiment, all cells were treated at the same time with the test compound or vehicle (0.1% DMSO) and plates returned to the cell culture incubator for 18 h. For analysis of secretory function in U87-MG-GLuc cells at 18 h, 20 μL of conditioned cell culture medium was removed from each well and transferred to new 96-well white-walled plates. Subsequently, 50 μL of 1.68 μM coelenterazine was injected into each well (final concentration = 1.2 μM), and luminescent signals were measured using a multi-mode microplate reader (Biotek Synergy HT) with Gen5® software and compared across conditions (3 sec wait, 0.5 sec integration time following coelenterazine injection). The viability of U87-MG-GLuc cells was measured at 18 h using a CellTiter-Glo® Luminescent Cell Viability Assay (Promega Corp) and luminescent signals measured using the same microplate reader. Concentration-response relationships were analyzed using GraphPad Prism Software version 9.5.1 (GraphPad Software, Inc., San Diego, CA), and IC_{50} values derived using nonlinear regression analysis fit to a logistic equation.

Detection of VEGF-using an enzyme-linked immunoassay (ELISA).

Human SF-268 glioblastoma cells were obtained from the National Cancer Institute (NCI) cell line repository (Fredrick, MD) and cultured in Minimal Essential Medium (MEM) supplemented with 10% FBS and 1% P/S. Cells were seeded into a 24-well plate and allowed to adhere overnight. The next day cells were treated with test compound, or vehicle (0.1% DMSO), and plates returned to the cell culture incubator for 24 h. For analysis of endogenously-secreted VEGF-A, the cell culture medium was harvested, cleared by centrifugation at 2,000 RPM for 10 min. and the supernatant analyzed for VEGF-A expression using an ELISA kit (Thermo Fisher Scientific; #BMS277) according to the manufacturer's instructions.

References

S1. Li, P. X.; Evans, C. D.; Wu, Y. Z.; Cao, B.; Hamel, E.; Joullie, M. M. Evolution of the total syntheses of ustiloxin natural products and their analogues. *J. Am. Chem. Soc.* **2008**, *130*, 2351-2364.

S2. Hu, X. E.; Kim, N. K.; Ledoussal, B. Synthesis of *trans*-(3*S*)-amino-(4*R*)-alkyl and -(4*S*)-aryl-piperidines via ring-closing metathesis reaction. *Org. Lett.* **2002**, *4*, 4499-4502.

S3. Biron, E.; Chatterjee, J.; Kessler, H. Optimized selective *N*-methylation of peptides on solid support. *J. Pept. Sci.* **2006**, *12*, 213-219.

S4. Hou, Z.; Nakanishi, I.; Kinoshita, T.; Takei, Y.; Yasue, M.; Misu, R.; Suzuki, Y.; Nakamura, S.; Kure, T.; Ohno, H.; Murata, K.; Kitaura, K.; Hirasawa, A.; Tsujimoto, G.; Oishi, S.; Fujii, N. Structure-based design of novel potent protein kinase CK2 (CK2) inhibitors with phenyl-azole scaffolds. *J. Med. Chem.* **2012**, *55*, 2899-2903.

S5. Badr, C. E.; Hewett, J. W.; Breakefield, X. O.; Tannous, B. A. A highly sensitive assay for monitoring the secretory pathway and ER stress. *PLoS One* **2007**, *2*, e571.

S6. Kazemi, S.; Kawaguchi, S.; Badr, C. E.; Mattos, D. R.; Ruiz-Saenz, A.; Serrill, J. D.; Moasser, M. M.; Dolan, B. P.; Paavilainen, V. O.; Oishi, S.; McPhail, K. L.; Ishmael, J. E. Targeting of HER/ErbB family proteins using broad spectrum Sec61 inhibitors coibamide A and apratoxin A. *Biochem. Pharmacol.* **2021**, *183*, 114317.

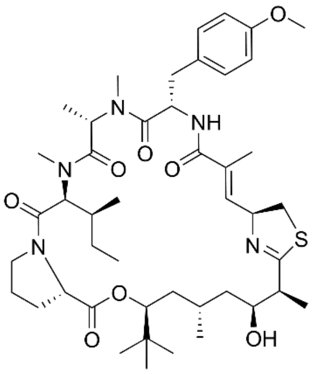

apratoxin A (**S15**)

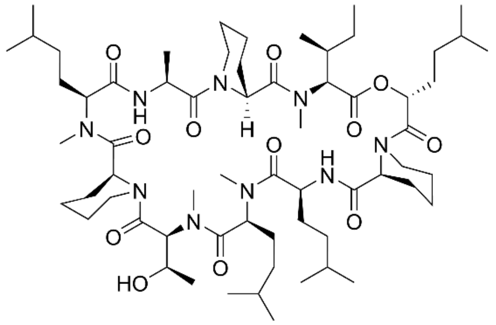

decatransin (**S16**)

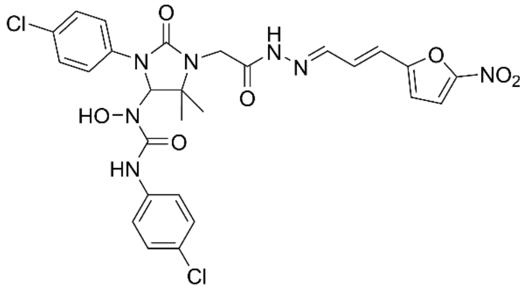

eeyarestatin I (**S17**)

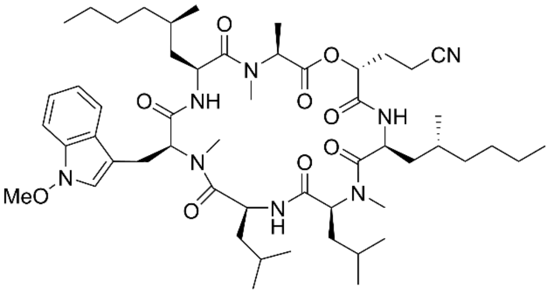

HUN-7293/pestahivin (**S18**)

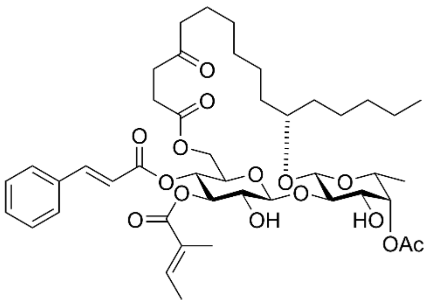

ipomoeassin F (**S19**)

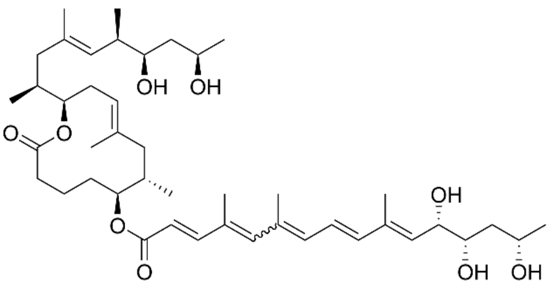

mycolactone A/B (**S20**)

Figure S3. Molecular structures of natural product Sec61α inhibitors.

Table S1. Cytotoxic activity of coibamide A derivatives against human U87-MG, HCT116, A549 cancer cells and normal human dermal fibroblasts (HDFs).

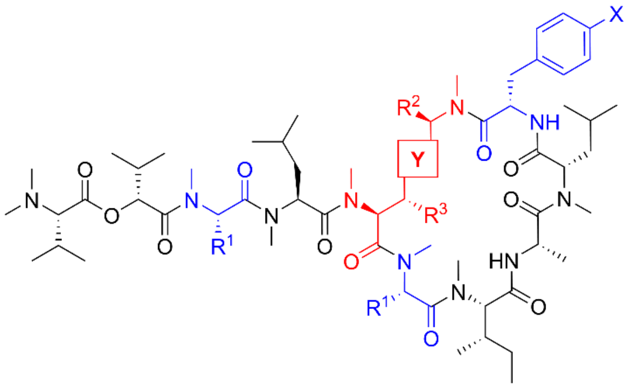

Peptide	R[1]	R[2]	R[3]	X	Y	A549 CC$_{50}$ (nM)[b]	U87-MG CC$_{50}$ (nM)[a]	HCT116 CC$_{50}$ (nM)[a]	HDF CC$_{50}$ (nM)[a]
**1a**	CH_2OMe	Me	Me	OMe	-CO-O-	1.5 ± 0.4	50 ± 6	2.7 ± 0.5	6.7 ± 2.1
**1b**	Me	Me	Me	OMe	-CO-O-	3.1 ± 0.8	2.6 ± 0.1	3.9 ± 0.5	4.6 ± 1.5
**2a**	Me	H	H	OMe	$-CH_2CH_2-$	420 ± 30	830 ± 110	2,300 ± 100	1,088 ± 115
**3a**	Me	Me	Me	OMe	$-CH_2CH_2-$	41 ± 4	56 ± 2	121 ± 23	213 ± 29
**3b**	Me	Me	Me	OMe	-(*E*)-CH=CH-	2.6 ± 0.4	6.2 ± 0.1	11 ± 1	8.3 ± 2.1
**16a**	Me	Me	Me	OMe	-CO-NH-	3.6 ± 1.4	3.9 ± 0.5	4.5 ± 0.5	4.6 ± 1.4
**16b**	CH_2OMe	Me	Me	OMe	-CO-NH-	1.3 ± 0.25	2.2 ± 0.4	3.4 ± 0.6	3.9 ± 1.5
**16c**	CH_2OMe	Me	Me	Ph	-CO-NH-	0.27 ± 0.10	0.6 ± 0.2	0.80 ± 0.23	0.38 ± 0.08
**17a**	Me	Me	H	OMe	-CO-NH-	48 ± 10	48 ± 3	60 ± 4	123 ± 25

[a] Cytotoxic Concentration (CC$_{50}$) values are the concentrations for 50% viability after 72-hr incubation of cells (n = 3).

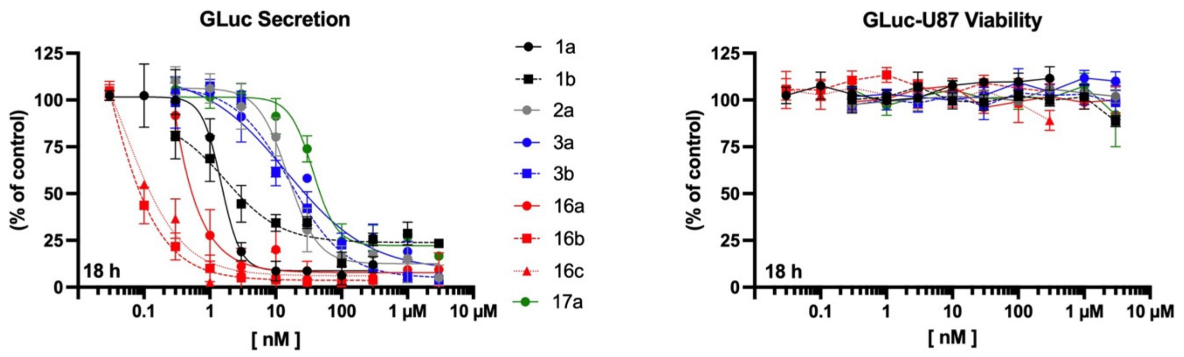

Figure S4. Inhibition of secretory function in viable cells by coibamide A (CbA) derivatives. Human U87-MG cells expressing *Gaussia Luciferase* (GLuc) under the control of a CMV promoter were treated with, or without (0.1% DMSO), increasing concentrations of CbA (**1a**) or CbA derivatives as indicated for 18 h under standard growth conditions. Dual assays were used to assess (A) GLuc activity from aliquots of the conditioned cell culture medium and (B) the viability of adherent U87-GLuc-expressing cells at the end-point (18 h) of the study. Data points show mean luminescence ± S.E. (*n* = 3 wells per treatment) expressed as a percentage (%) of control (vehicle-treated) cells. Graph shows a single comparison of all synthetic derivatives that was repeated three times.

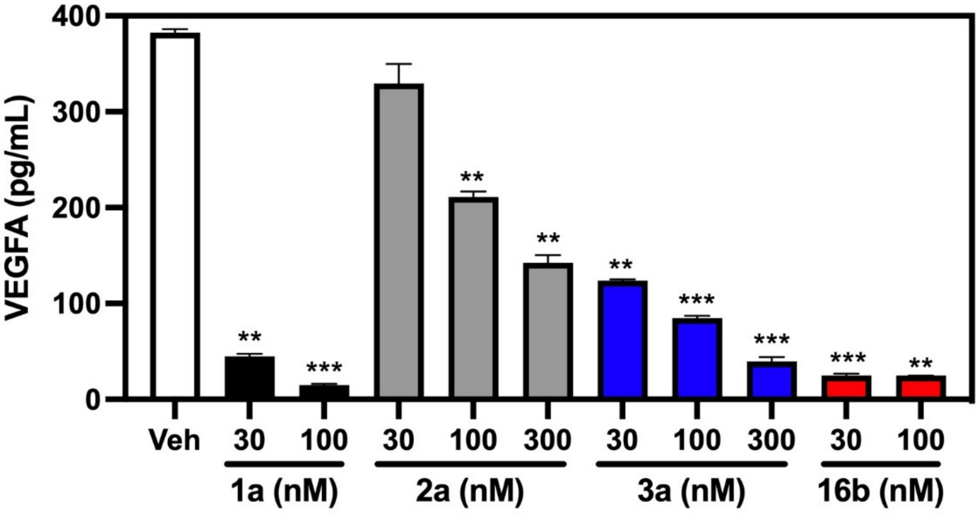

Figure S5. CbA derivatives induce concentration-dependent decreases in endogenous VEGF-A secreted from human SF-296 glioblastoma cells.

Human SF-268 glioblastoma cells were treated with CbA (**1a**), **2a**, **3a**, **16b** or vehicle (0.1% DMSO) for 24 h under standard growth conditions. For analysis of secreted VEGF-A, the cell culture medium was harvested, cleared by centrifugation at 2,000 RPM for 10 min and the supernatant analyzed for VEGF-A expression by ELISA (Thermo Fisher Scientific; #BMS277). Histogram shows quantification of VEGF-A expression (pg/mL) from a representative experiment that was repeated three times. Statistically significant differences in treated- versus vehicle (Veh)-treated cells is indicated as ** p< 0.01 or ***p< 0.001.

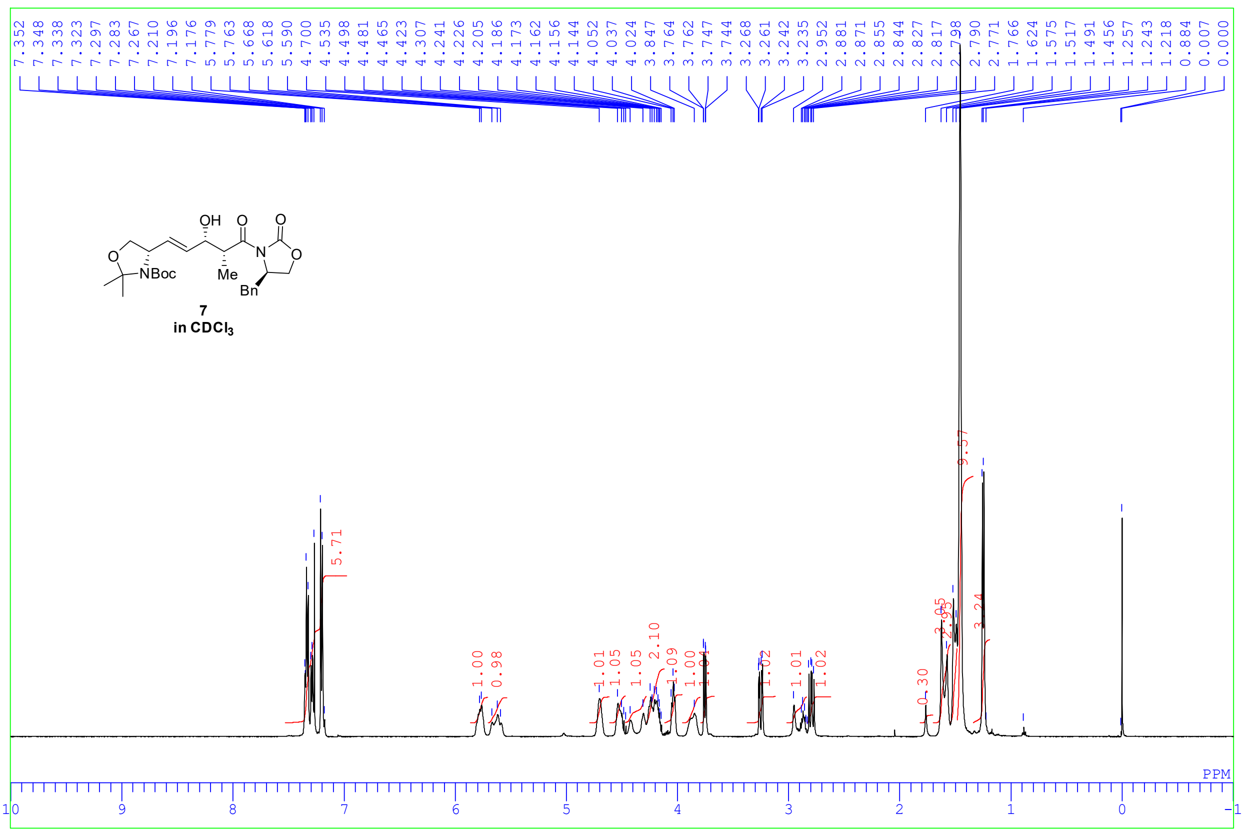

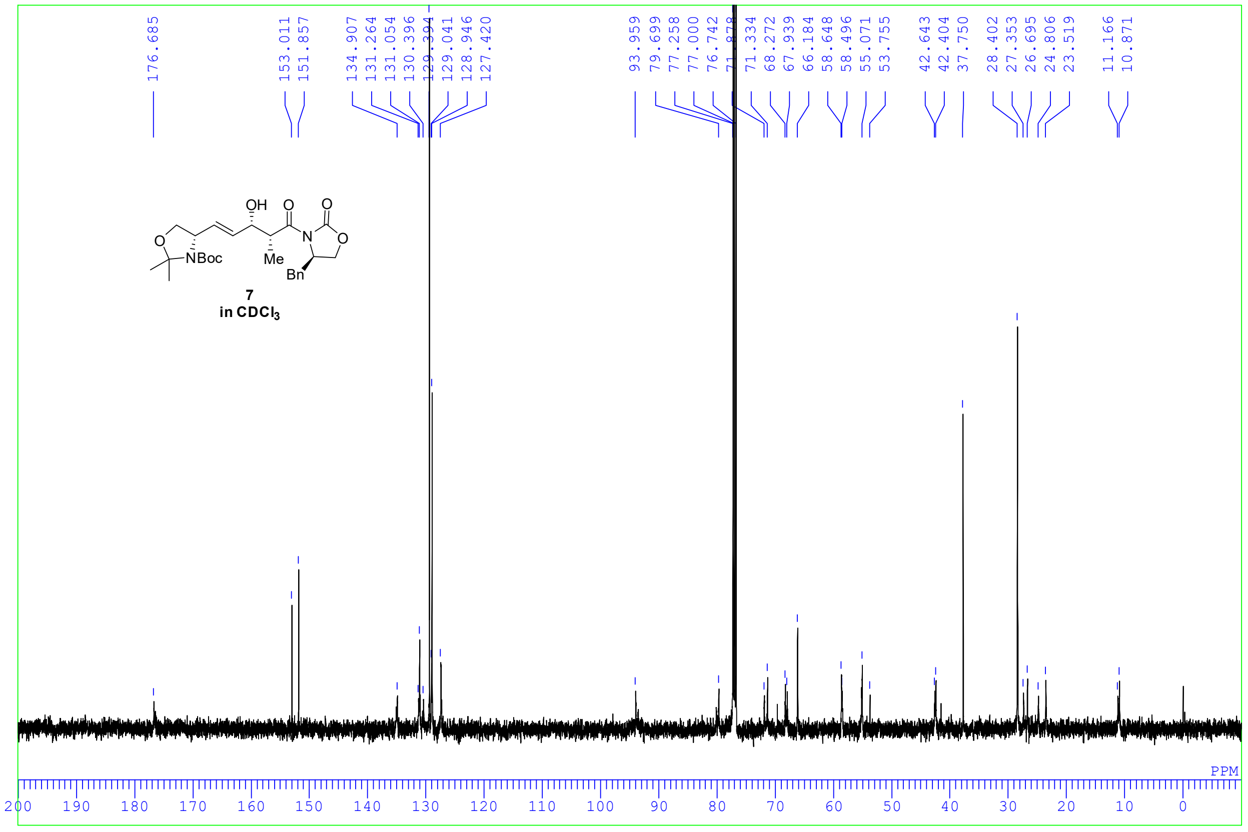

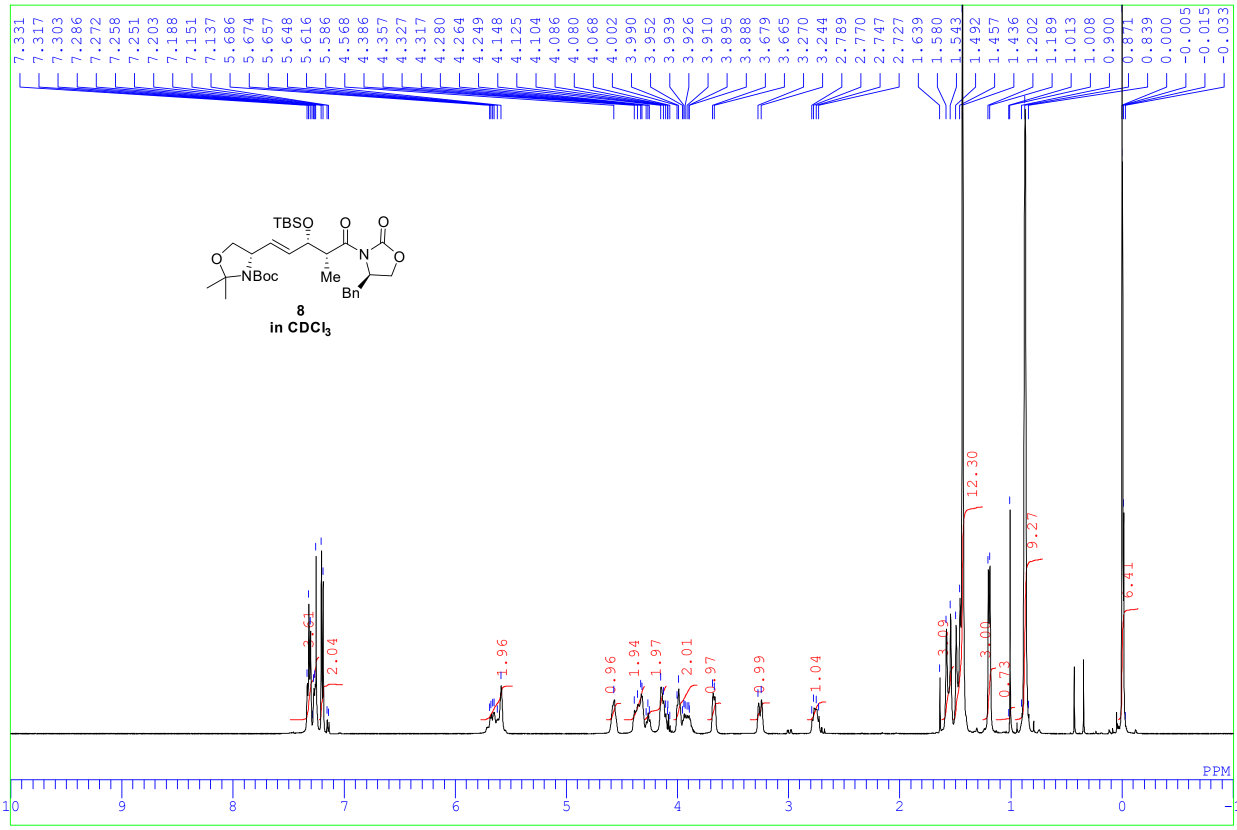

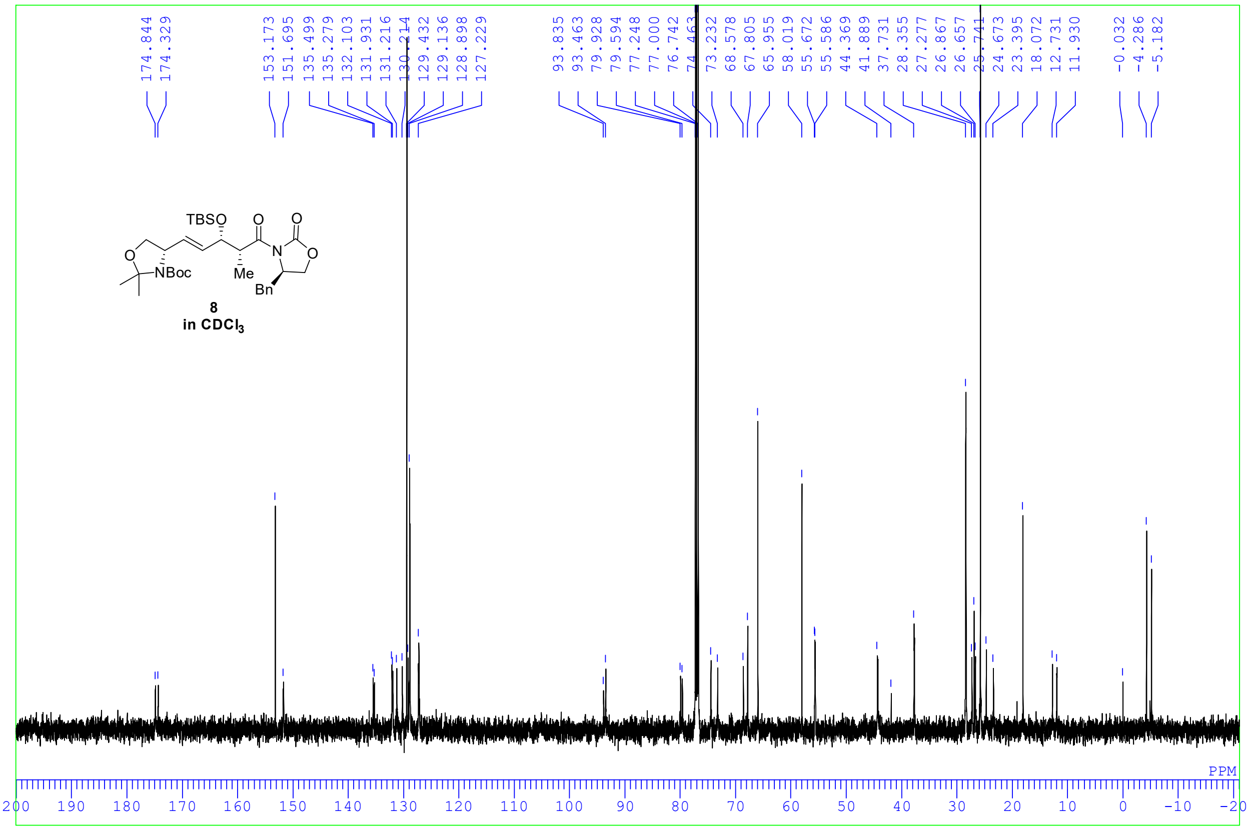

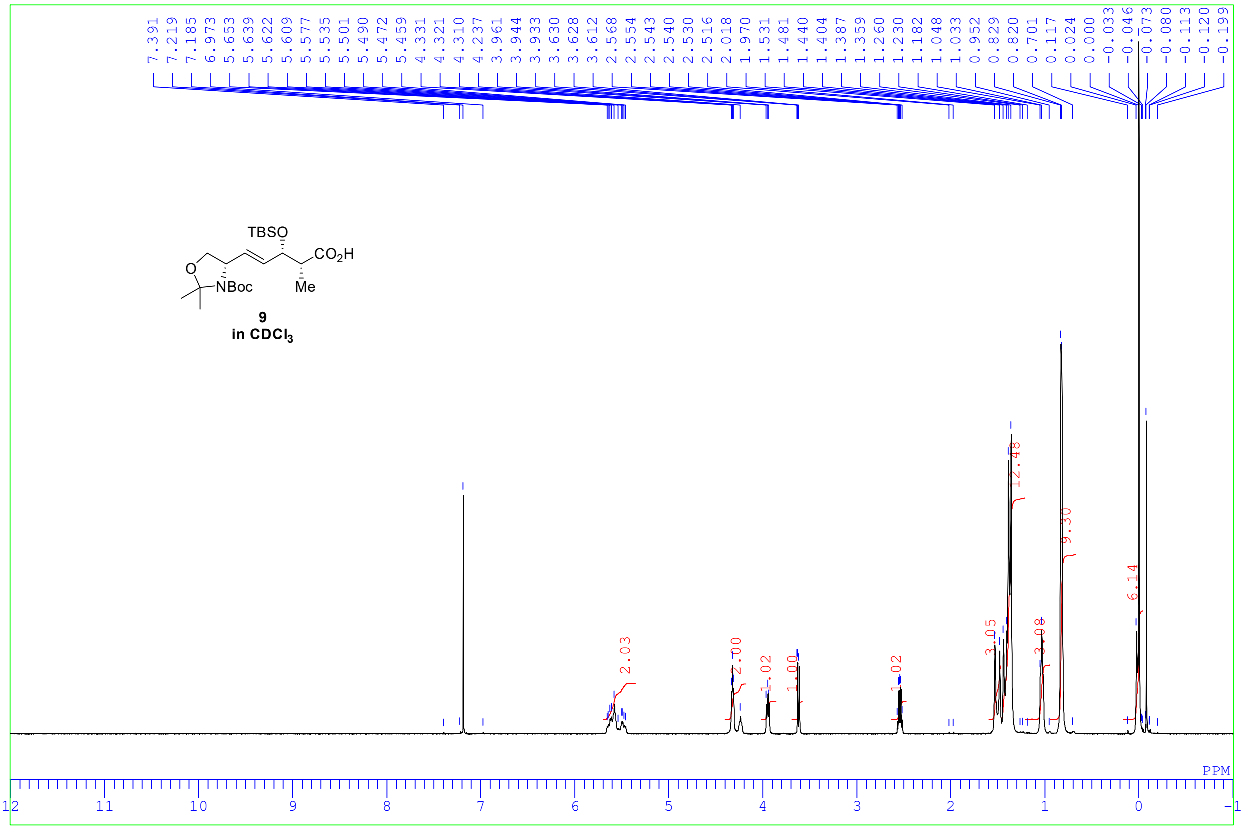
TBSO
CO_2H
O
NBoc
Me
9
in $CDCl_3$
PPM

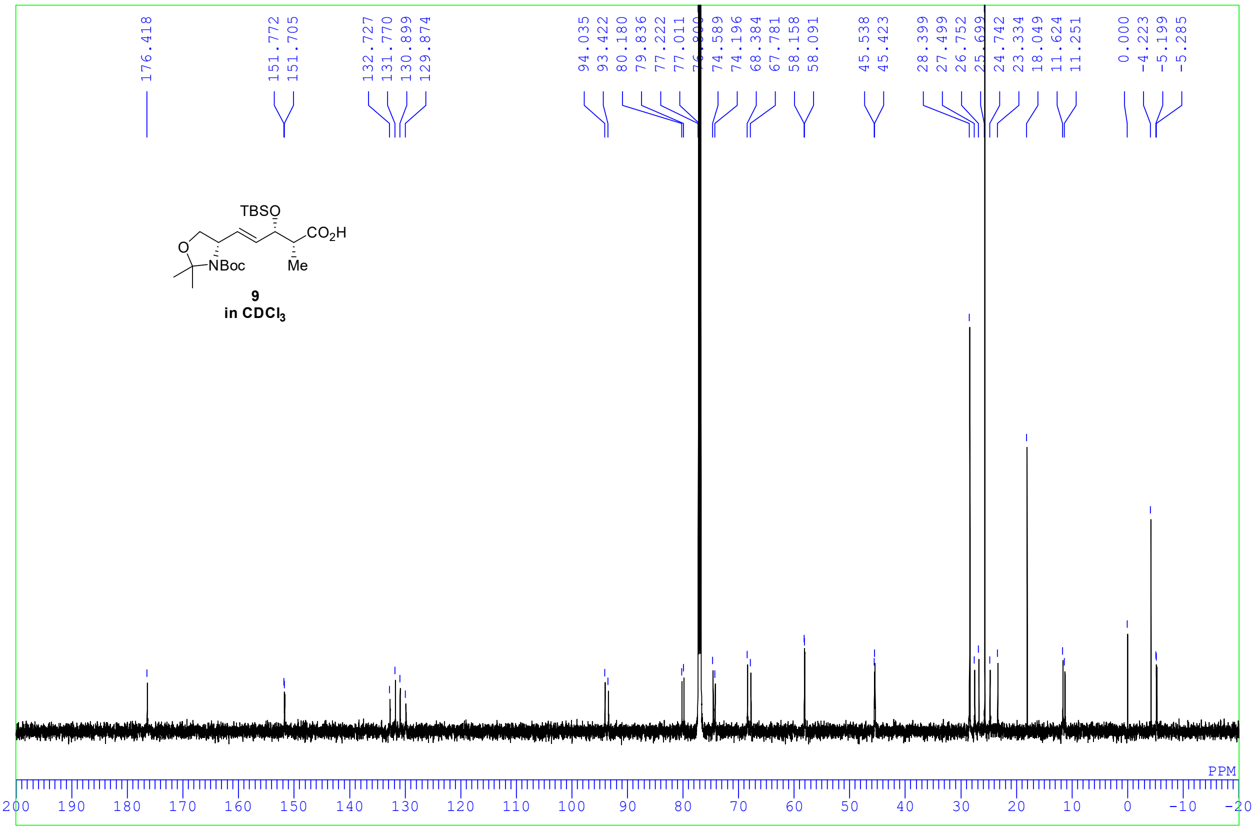
TBSO
CO_2H
O
NBoc
Me
9
in $CDCl_3$
PPM

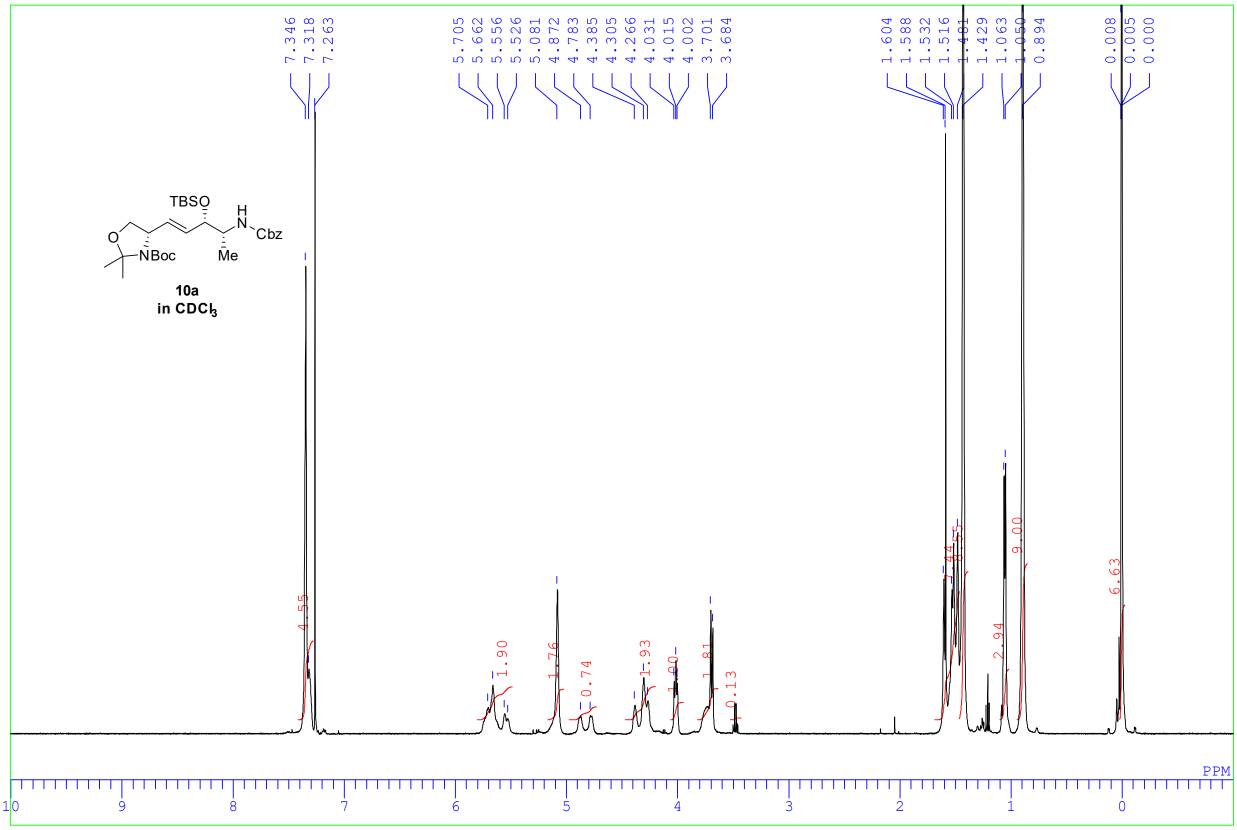
TBSO
H
N
Cbz
O
NBoc
Me
10a
in CDCl3
PPM

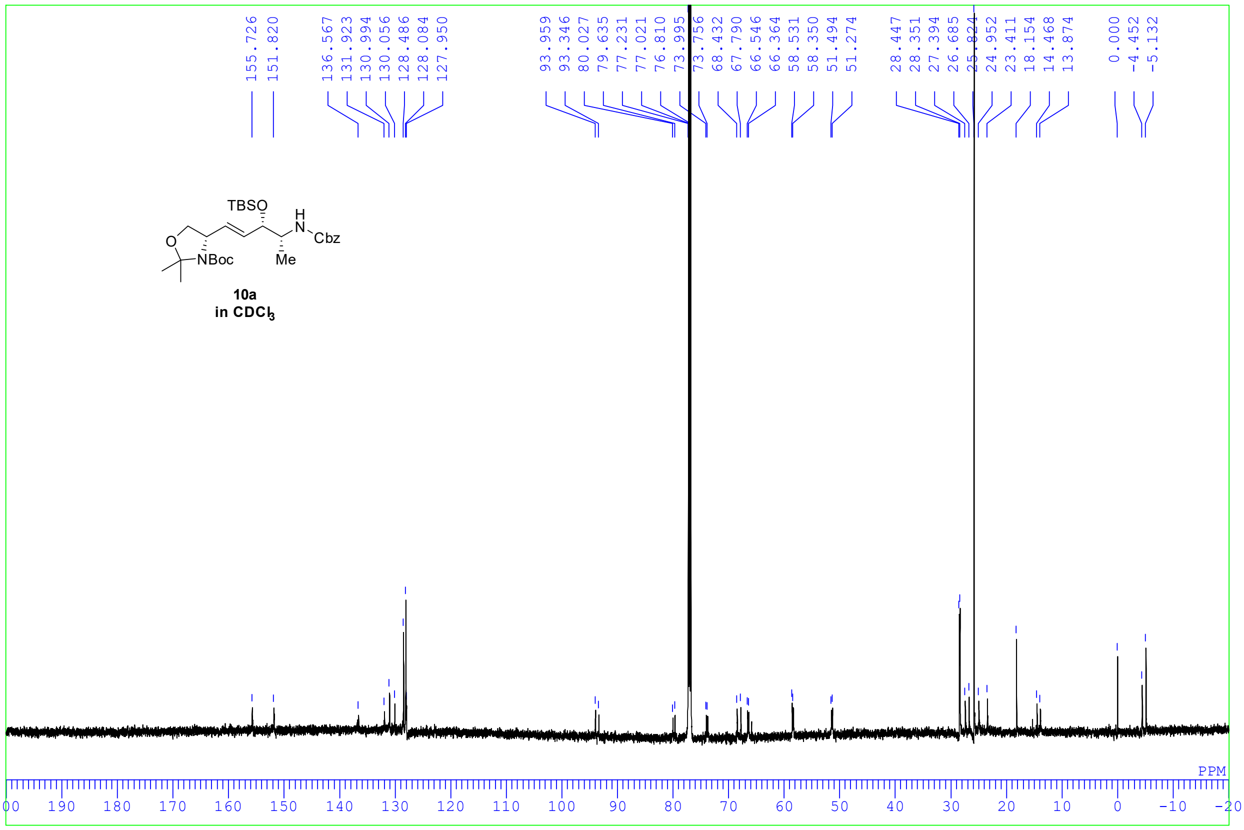
TBSO
H
N
Cbz
O
NBoc
Me
10a
in CDCl3
PPM

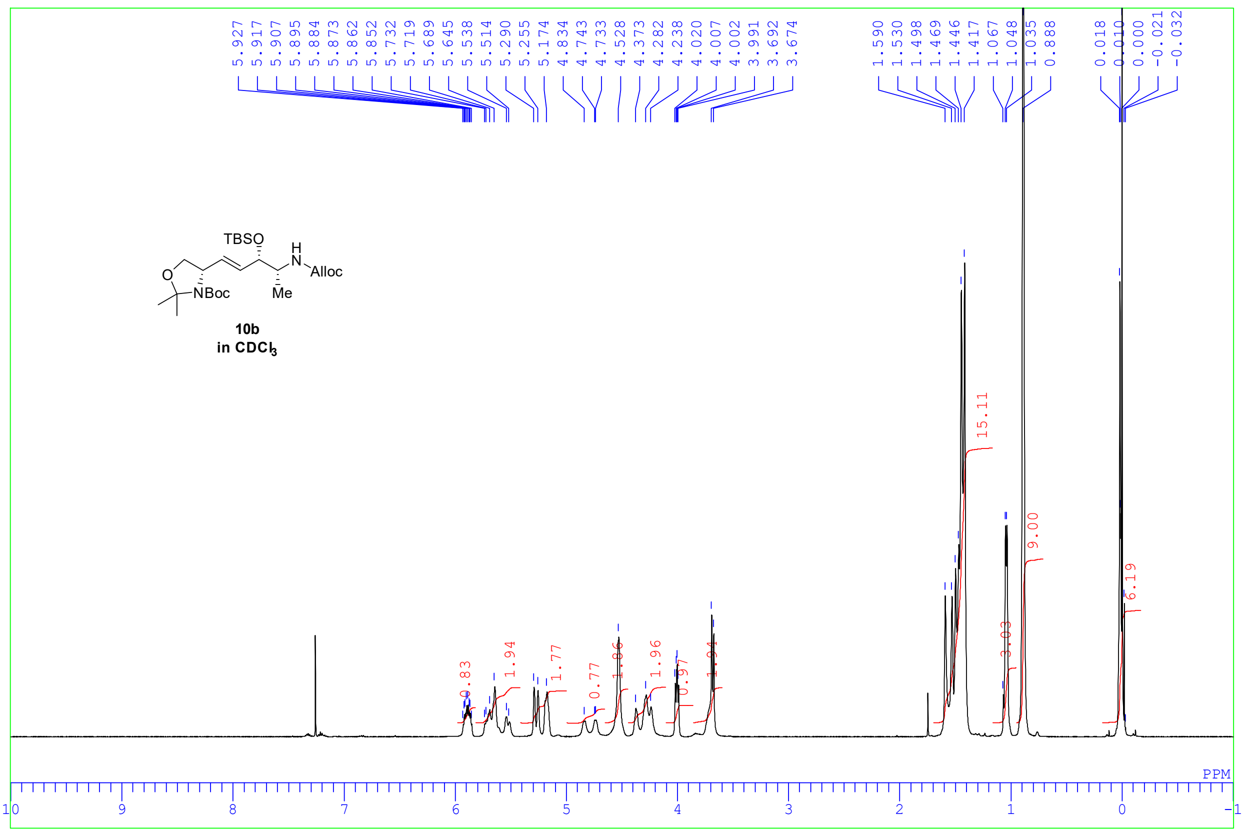

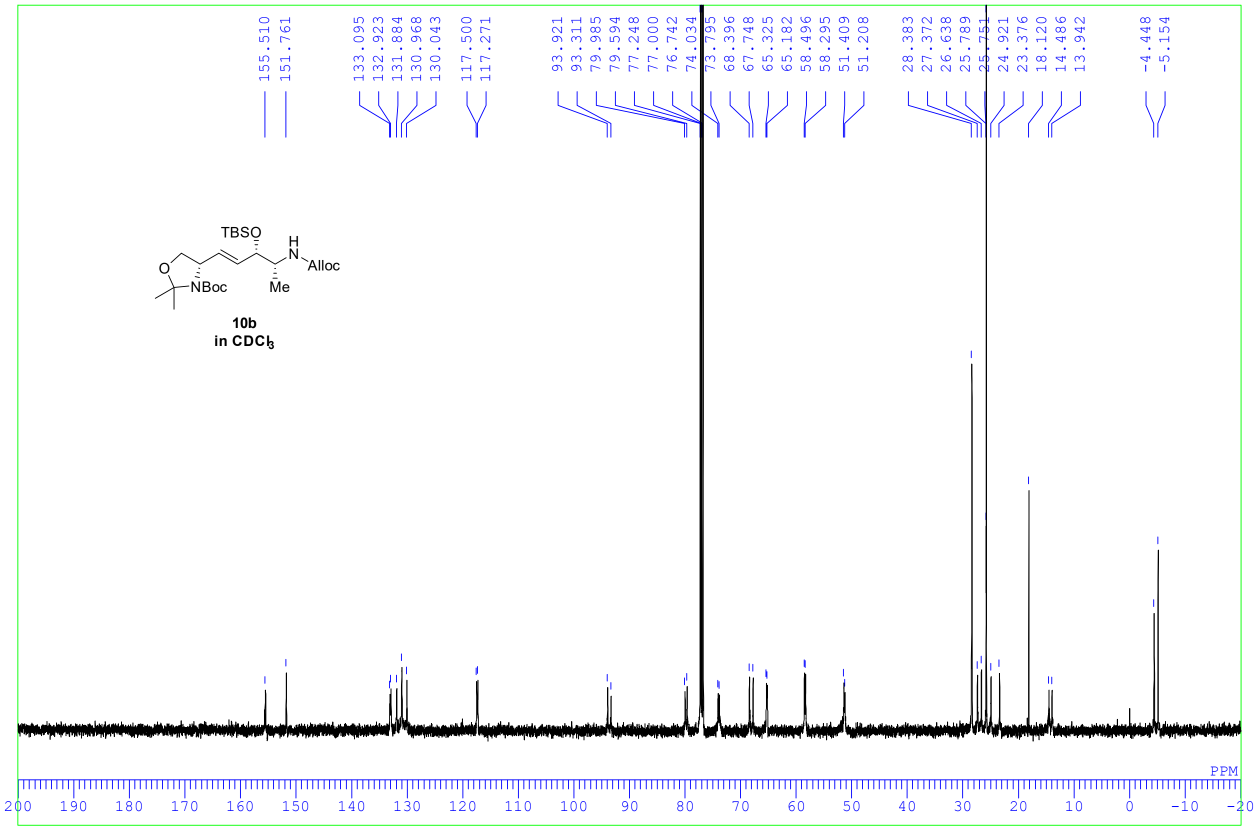

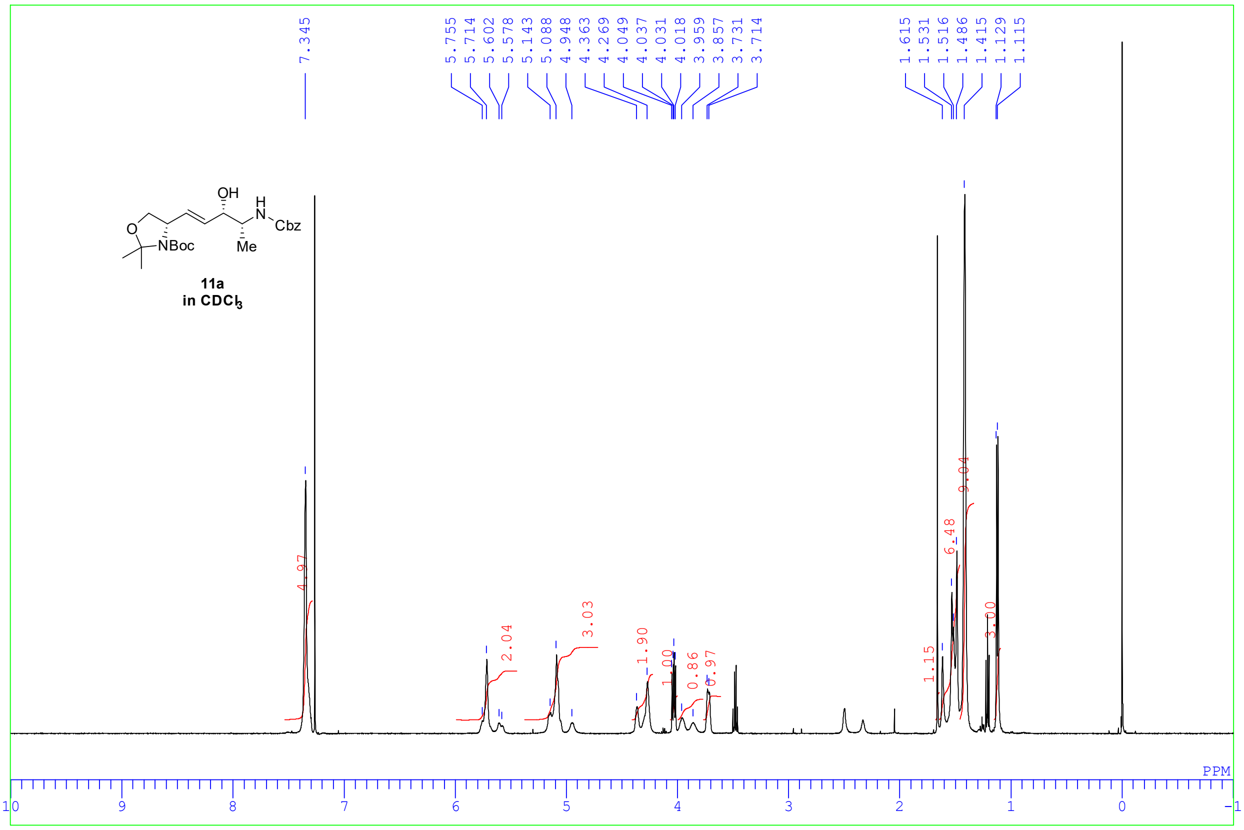

7.345
5.755
5.714
5.602
5.578
5.143
5.088
4.948
4.363
4.269
4.049
4.037
4.031
4.018
3.959
3.857
3.731
3.714
1.615
1.531
1.516
1.486
1.415
1.129
1.115
OH
H
N
Cbz
O
NBoc
Me
11a
in CDCl3
4.97
2.04
3.03
1.90
1.00
0.86
0.97
1.15
6.48
9.04
3.00
PPM

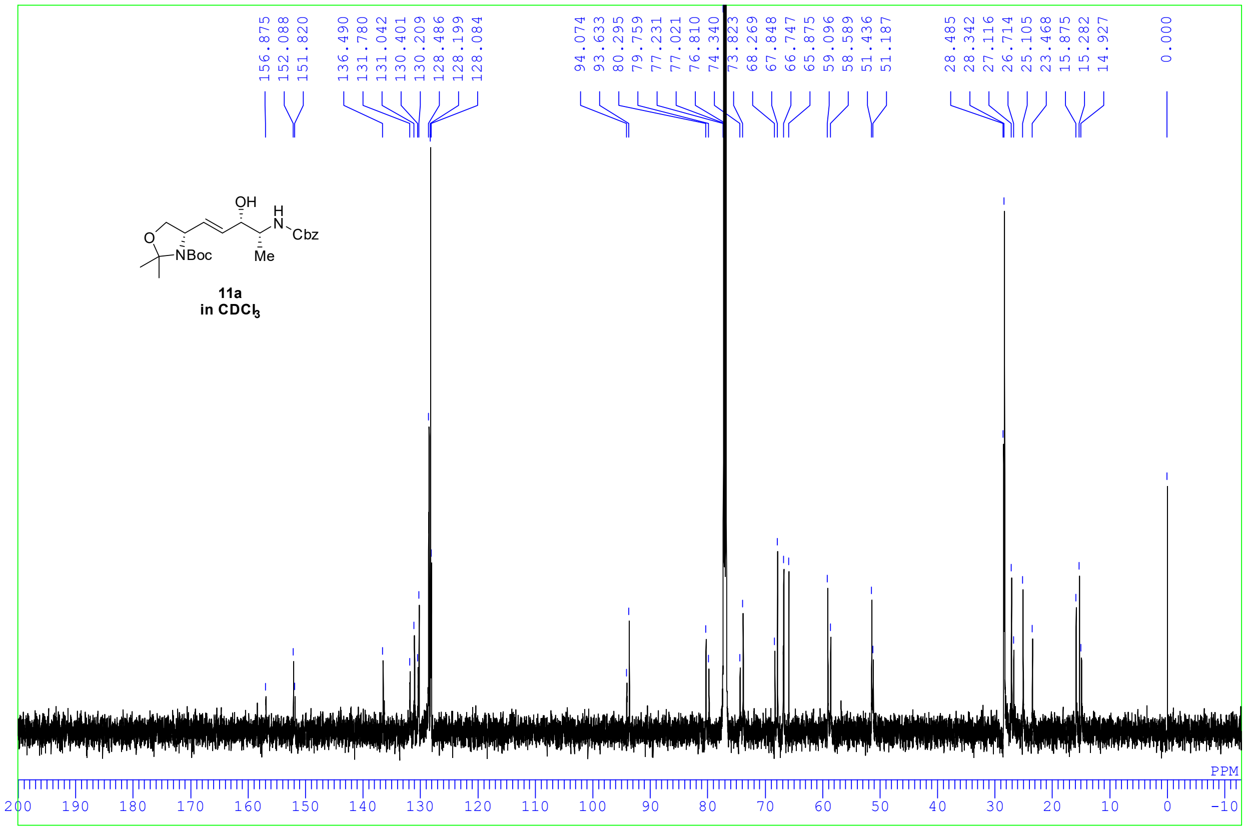

156.875
152.088
151.820
136.490
131.780
131.042
130.401
130.209
128.486
128.199
128.084
94.074
93.633
80.295
79.759
77.231
77.021
76.810
74.340
73.823
68.269
67.848
66.747
65.875
59.096
58.589
51.436
51.187
28.485
28.342
27.116
26.714
25.105
23.468
15.875
15.282
14.927
0.000
OH
H
N
Cbz
O
NBoc
Me
11a
in CDCl3
PPM

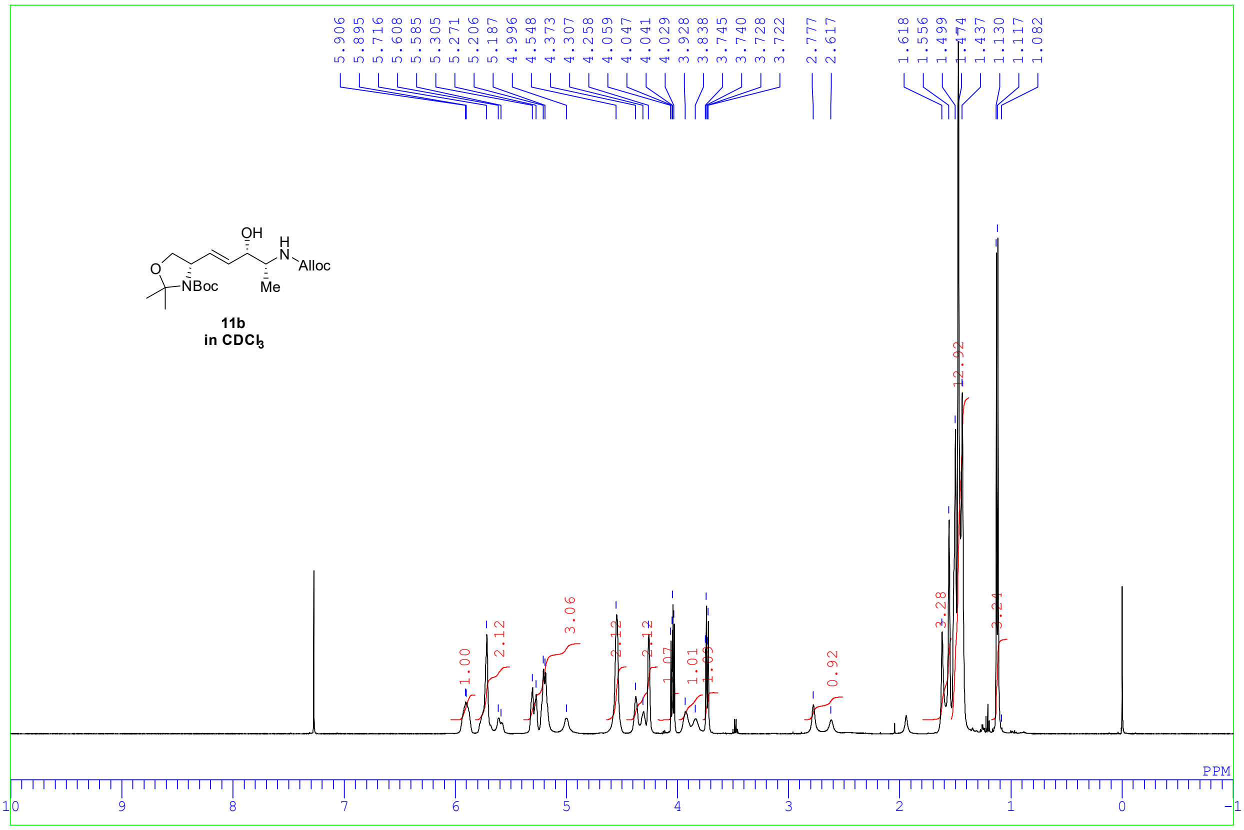
5.906 5.895 5.716 5.608 5.585 5.305 5.271 5.206 5.187 4.996 4.548 4.373 4.307 4.258 4.059 4.047 4.041 4.029 3.928 3.838 3.745 3.740 3.728 3.722 2.777 2.617 1.618 1.556 1.499 1.474 1.437 1.130 1.117 1.082
OH
H
N
Alloc
O
NBoc
Me
11b
in CDCl3
PPM

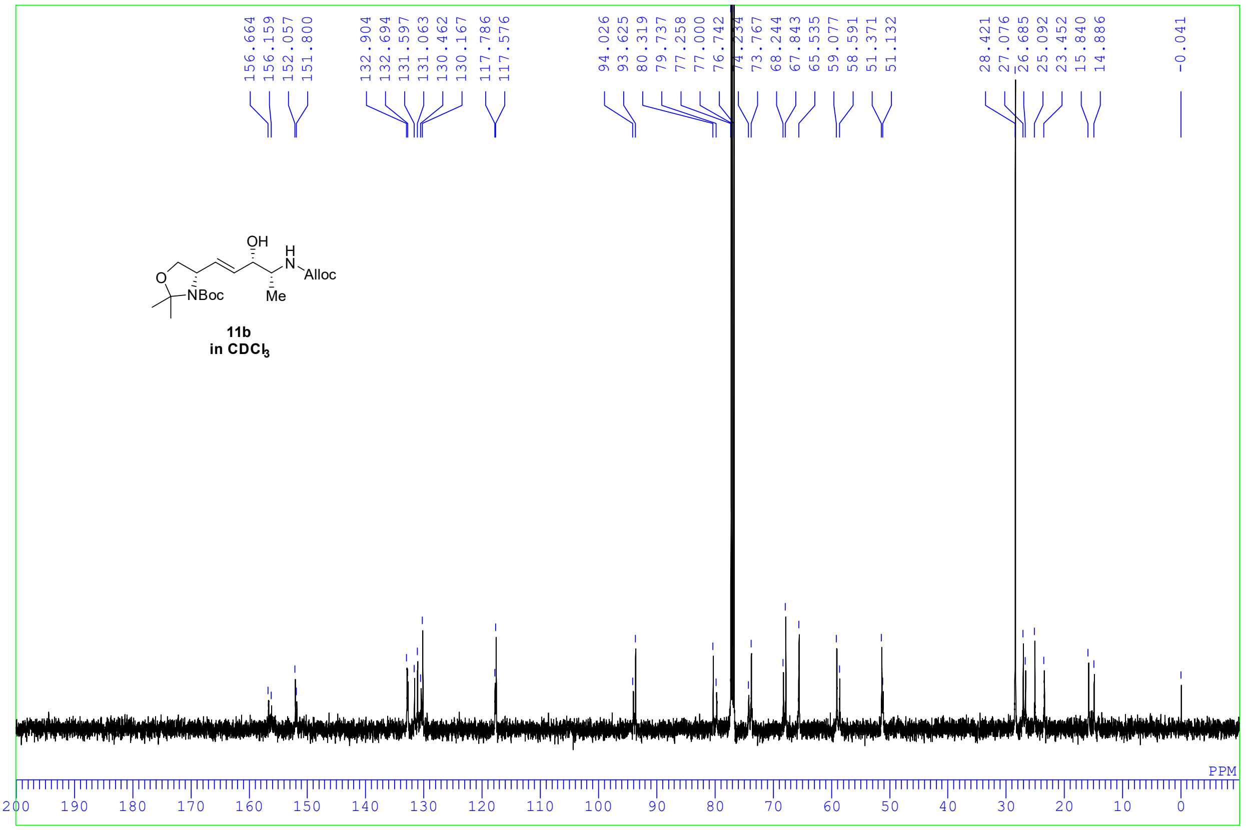
156.664 156.159 152.057 151.800 132.904 132.694 131.597 131.063 130.462 130.167 117.786 117.576 94.026 93.625 80.319 79.737 77.258 77.000 76.742 74.234 73.767 68.244 67.843 65.535 59.077 58.591 51.371 51.132 28.421 27.076 26.685 25.092 23.452 15.840 14.886 -0.041
OH
H
N
Alloc
O
NBoc
Me
11b
in CDCl3
PPM

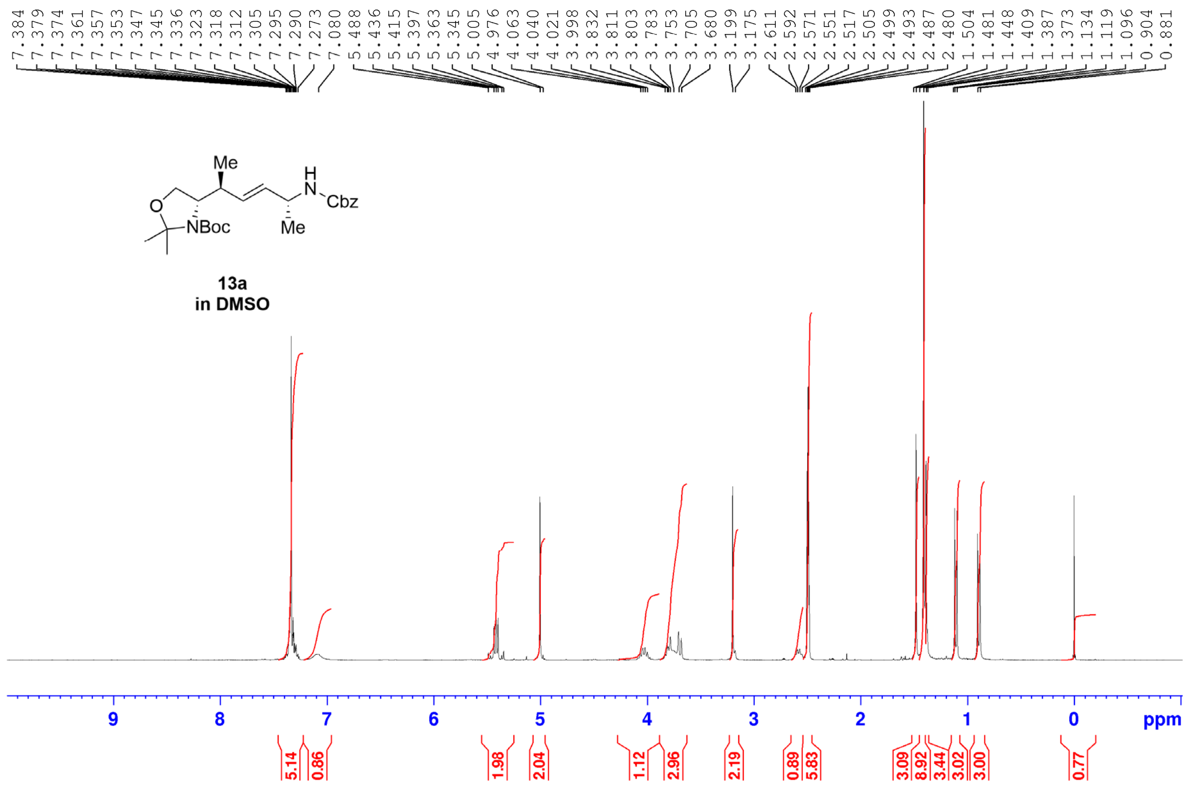

7.384 7.379 7.374 7.361 7.357 7.353 7.347 7.345 7.336 7.323 7.318 7.312 7.305 7.295 7.290 7.273 7.080 5.488 5.436 5.415 5.397 5.363 5.345 5.005 4.976 4.063 4.040 4.021 3.998 3.832 3.811 3.803 3.783 3.753 3.705 3.680 3.199 3.175 2.611 2.592 2.571 2.551 2.517 2.505 2.499 2.493 2.487 2.480 1.504 1.481 1.448 1.409 1.387 1.373 1.134 1.119 1.096 0.904 0.881
Me
H
N
Cbz
O
NBoc
Me
13a
in DMSO
9 8 7 6 5 4 3 2 1 0 ppm
5.14 0.86 1.98 2.04 1.12 2.96 2.19 0.89 5.83 3.09 8.92 3.44 3.02 3.00 0.77

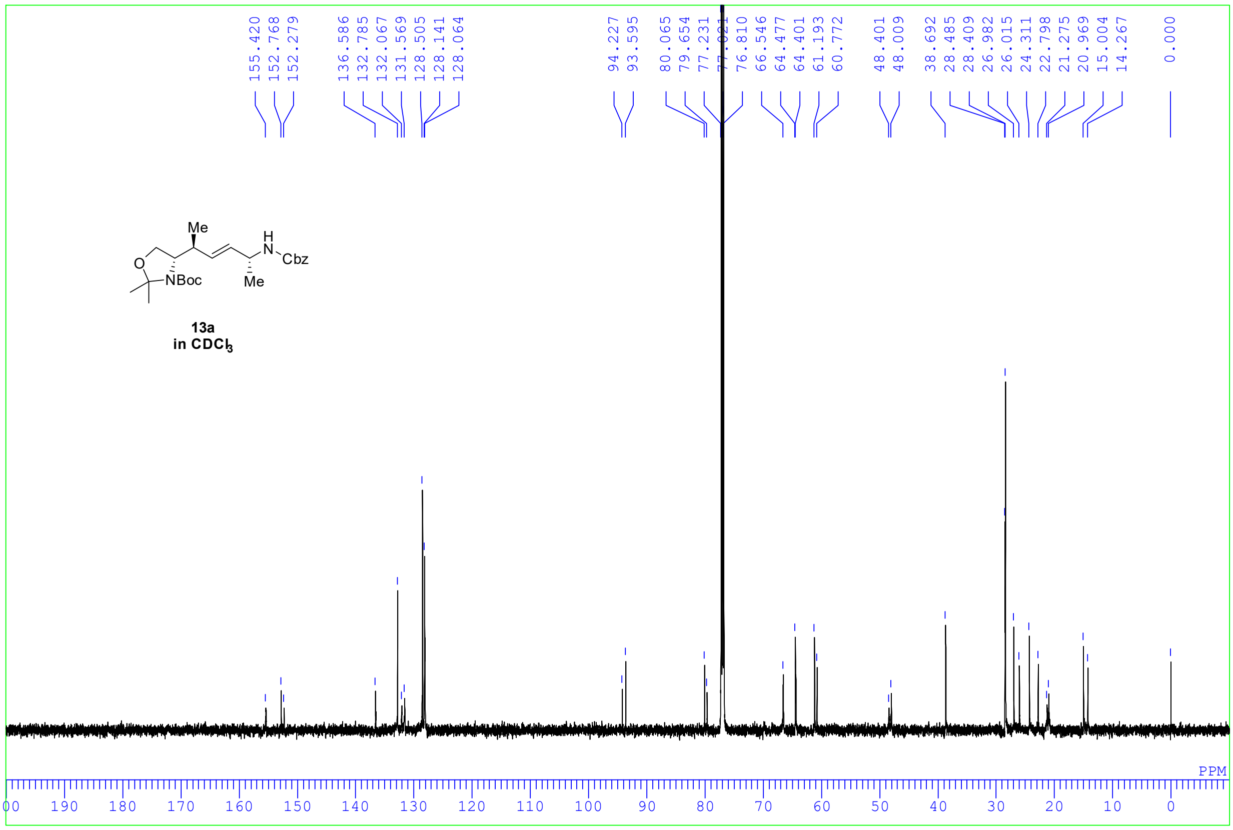

155.420 152.768 152.279 136.586 132.785 132.067 131.569 128.505 128.141 128.064 94.227 93.595 80.065 79.654 77.231 76.810 66.546 64.477 64.401 61.193 60.772 48.401 48.009 38.692 28.485 28.409 26.982 26.015 24.311 22.798 21.275 20.969 15.004 14.267 0.000
Me
H
N
Cbz
O
NBoc
Me
13a
in CDCl3
PPM
200 190 180 170 160 150 140 130 120 110 100 90 80 70 60 50 40 30 20 10 0

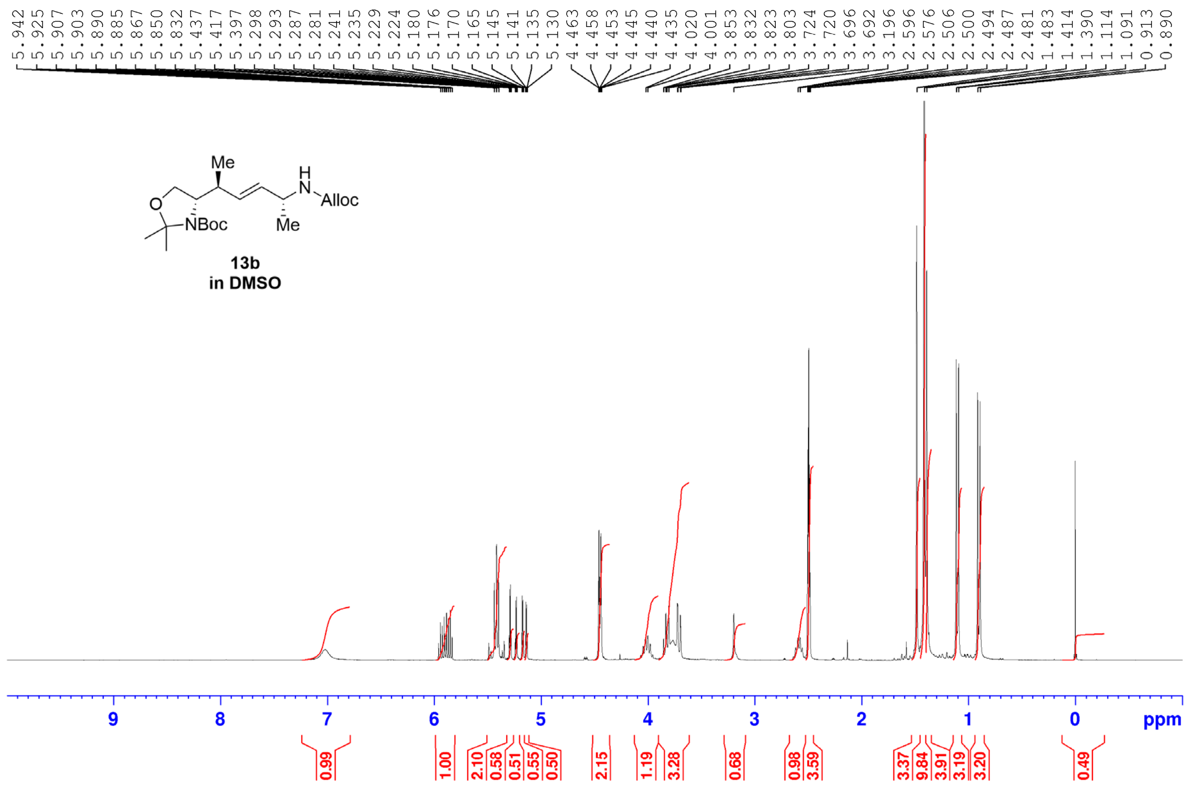
5.942
5.925
5.907
5.903
5.890
5.885
5.867
5.850
5.832
5.437
5.417
5.397
5.298
5.293
5.287
5.281
5.241
5.235
5.229
5.224
5.180
5.176
5.170
5.165
5.145
5.141
5.135
5.130
4.463
4.458
4.453
4.445
4.440
4.435
4.020
4.001
3.853
3.832
3.823
3.803
3.724
3.720
3.696
3.692
3.196
2.596
2.576
2.506
2.500
2.494
2.487
2.481
1.483
1.414
1.390
1.114
1.091
0.913
0.890
Me
H
N
Alloc
O
NBoc
Me
13b
in DMSO
9
8
7
6
5
4
3
2
1
0
ppm
0.99
1.00
2.10
0.58
0.51
0.55
0.50
2.15
1.19
3.28
0.68
0.98
3.59
3.37
9.84
3.91
3.19
3.20
0.49

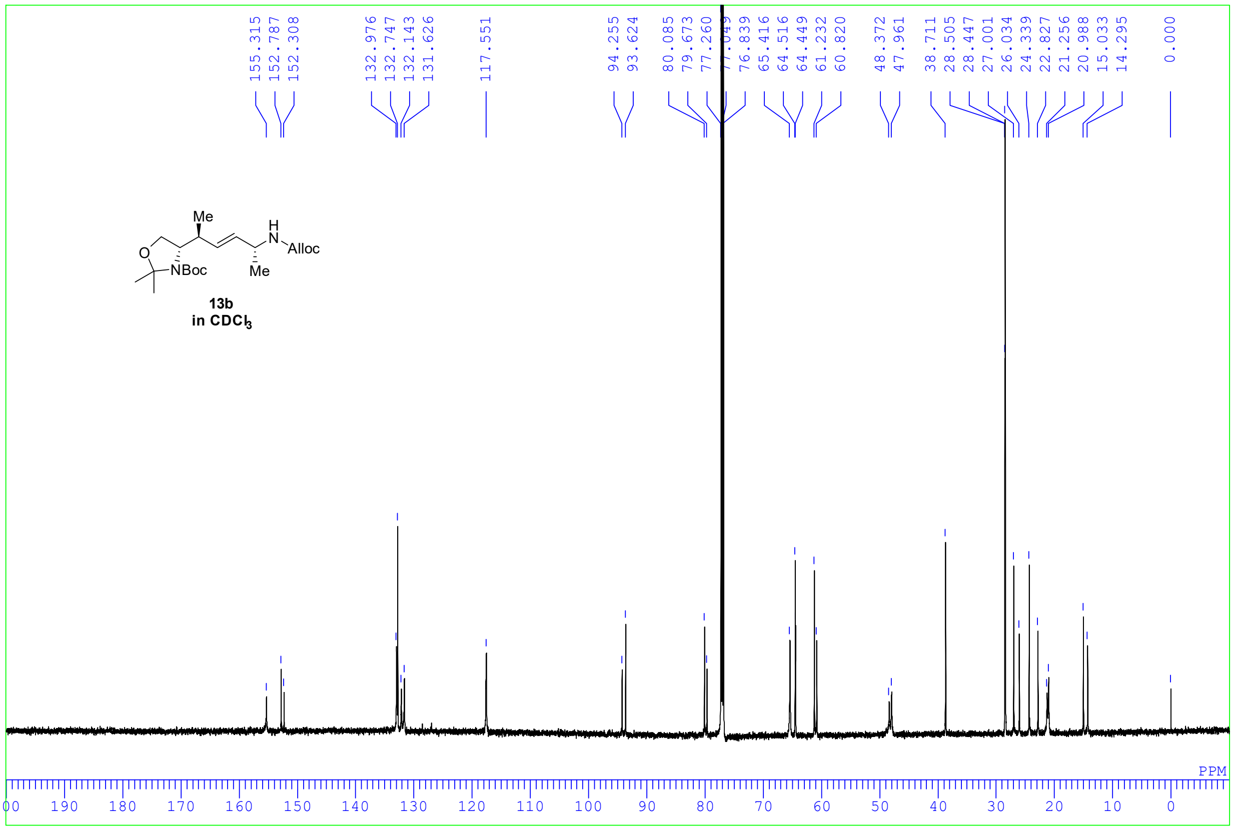
155.315
152.787
152.308
132.976
132.747
132.143
131.626
117.551
94.255
93.624
80.085
79.673
77.260
77.049
76.839
65.416
64.516
64.449
61.232
60.820
48.372
47.961
38.711
28.505
28.447
27.001
26.034
24.339
22.827
21.256
20.988
15.033
14.295
0.000
Me
H
N
Alloc
O
NBoc
Me
13b
in $CDCl_3$
PPM
200
190
180
170
160
150
140
130
120
110
100
90
80
70
60
50
40
30
20
10
0

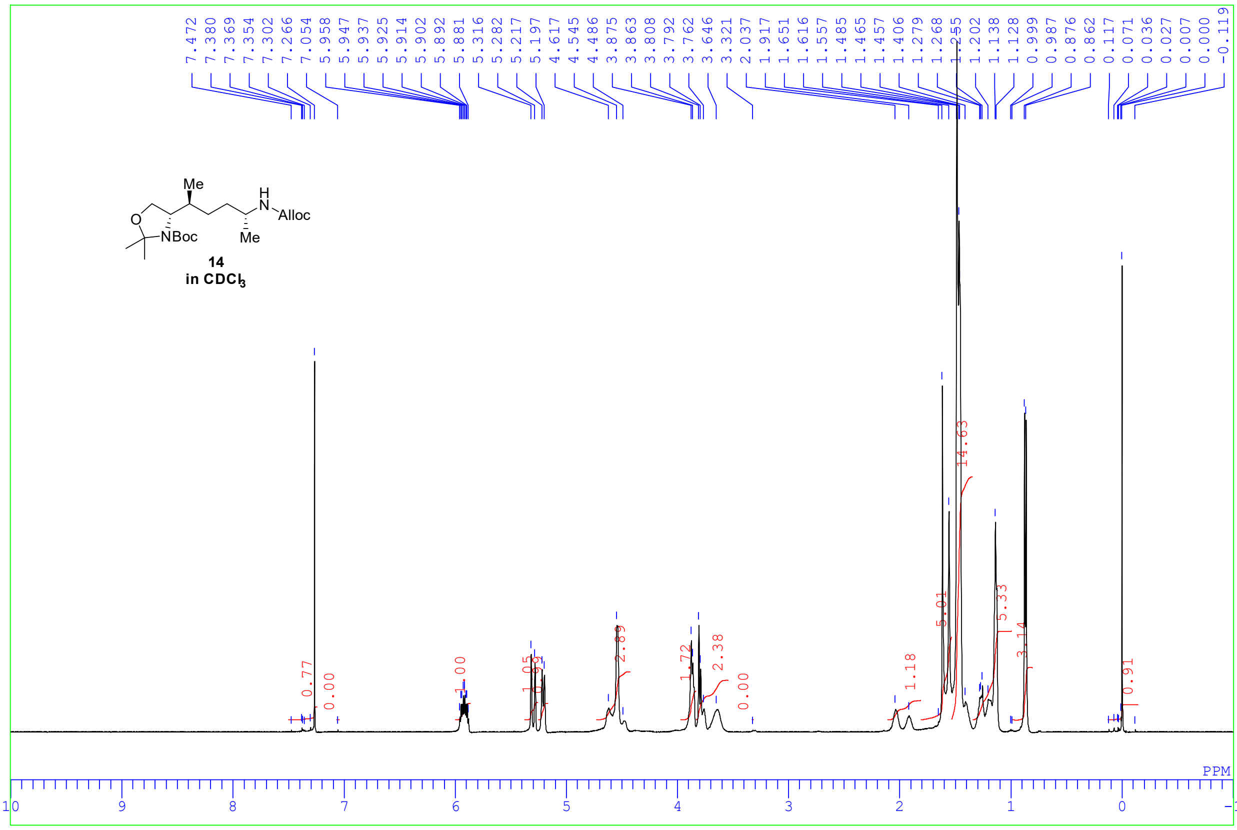

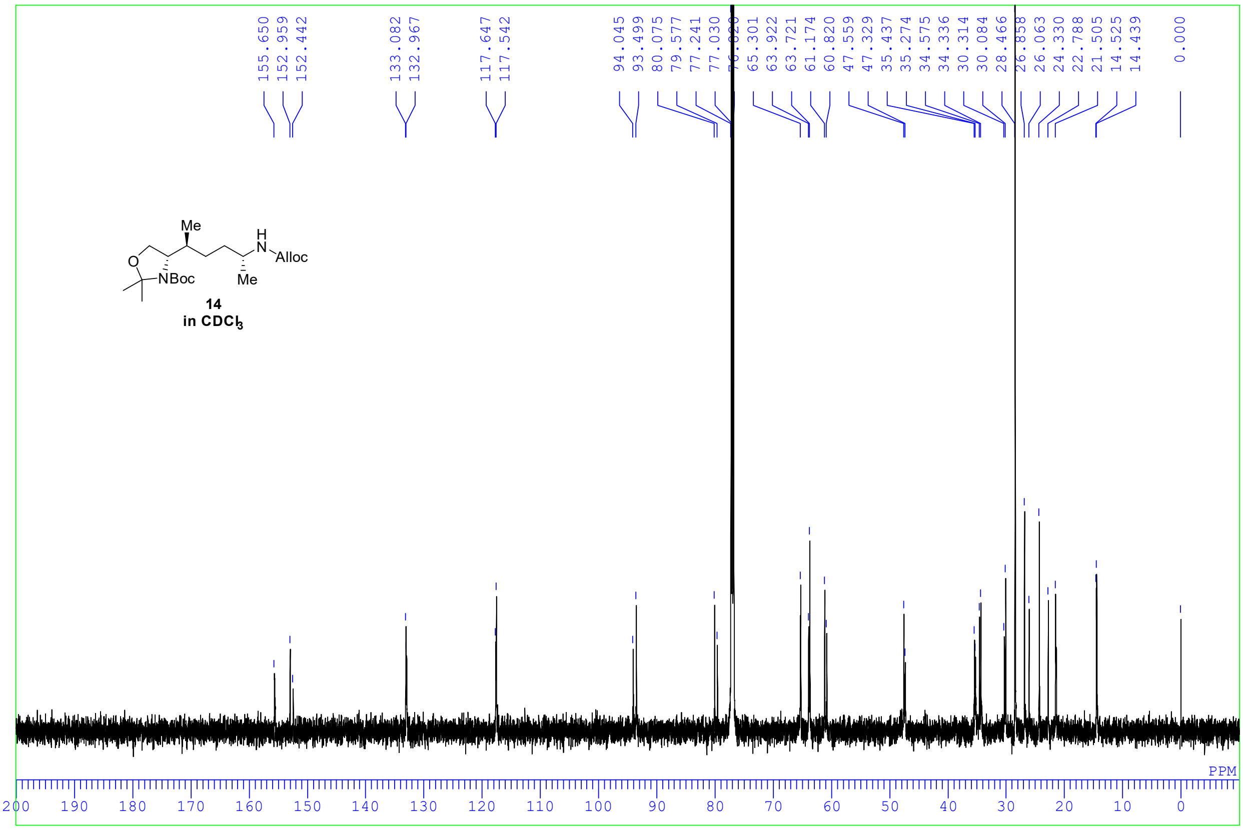

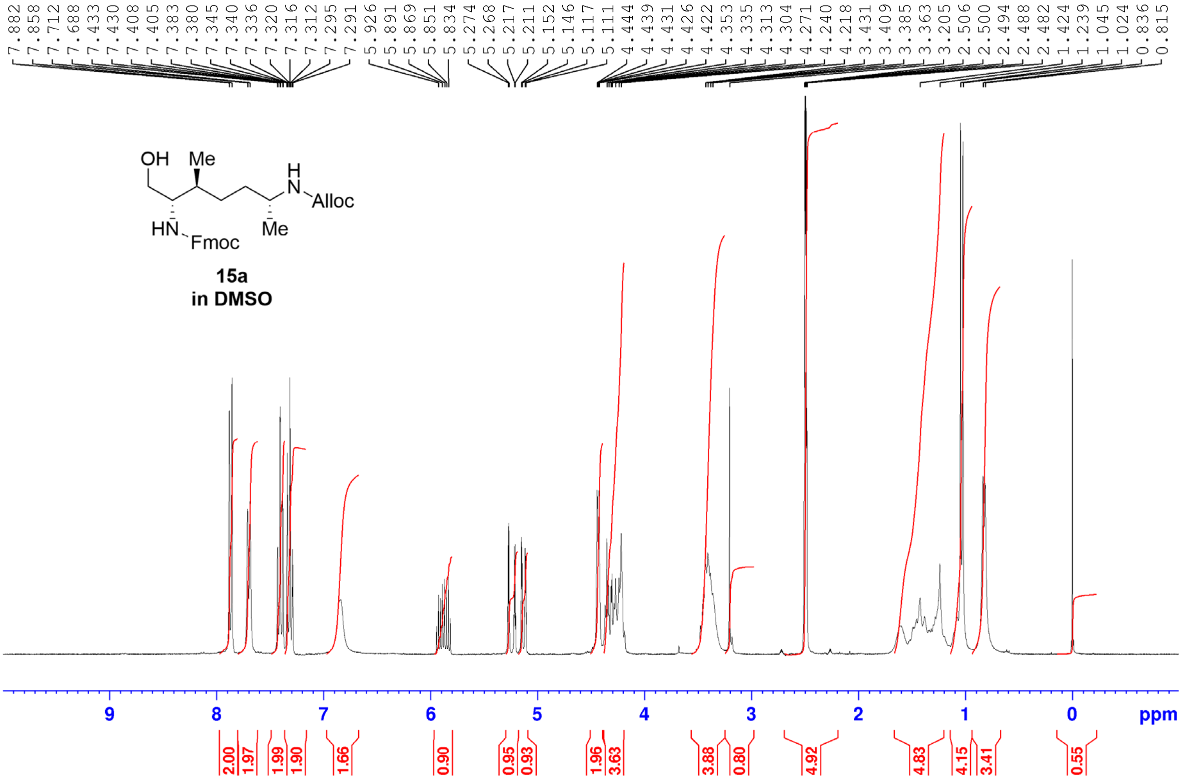
OH
Me
H
N
Alloc
HN
Fmoc
Me
15a
in DMSO
ppm

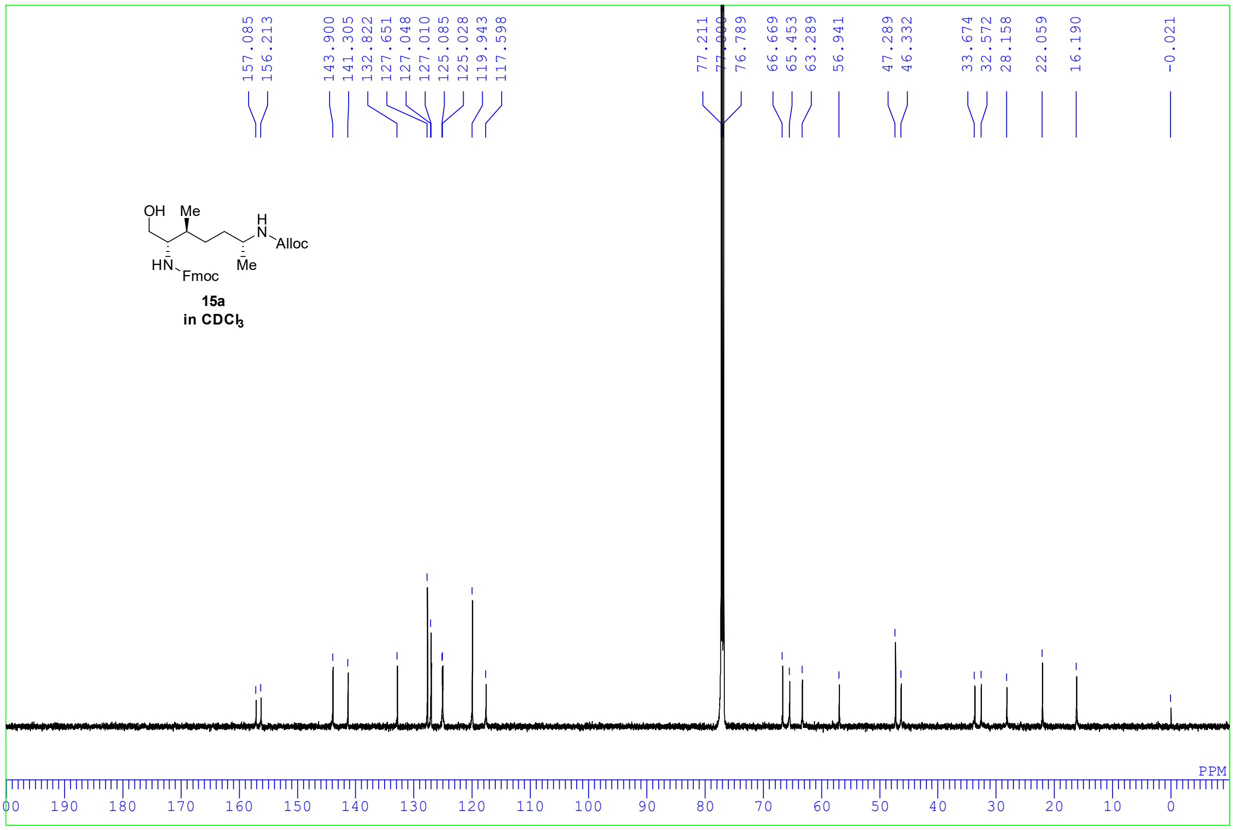
OH
Me
H
N
Alloc
HN
Fmoc
Me
15a
in CDCl3
PPM

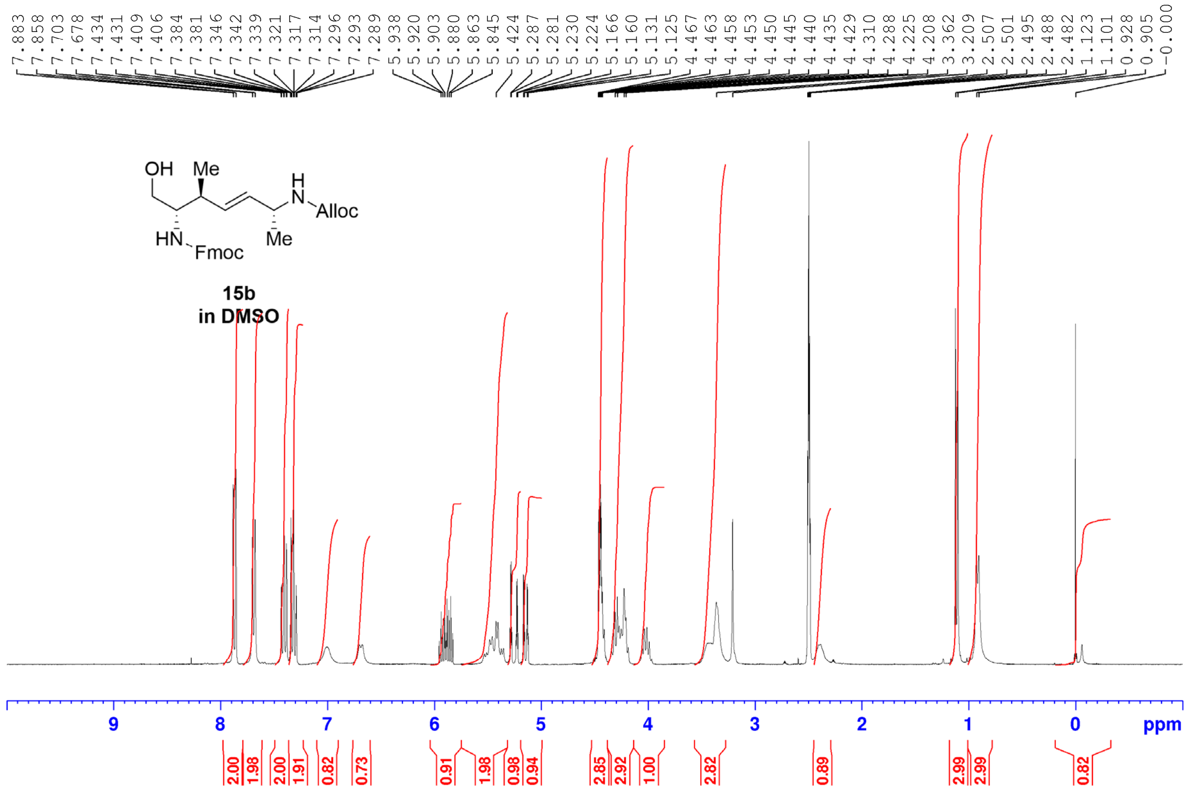
7.883 7.858 7.703 7.678 7.434 7.431 7.409 7.406 7.384 7.381 7.346 7.342 7.339 7.321 7.317 7.314 7.296 7.293 7.289 5.938 5.920 5.903 5.880 5.863 5.845 5.424 5.287 5.281 5.230 5.224 5.166 5.160 5.131 5.125 4.467 4.463 4.458 4.453 4.450 4.445 4.440 4.435 4.429 4.310 4.288 4.225 4.208 3.362 3.209 2.507 2.501 2.495 2.488 2.482 1.123 1.101 0.928 0.905 -0.000
OH Me
H
N
Alloc
HN
Fmoc
Me
15b
in DMSO
ppm
2.00 1.98 2.00 1.91 0.82 0.73 0.91 1.98 0.98 0.94 2.85 2.92 1.00 2.82 0.89 2.99 2.99 0.82

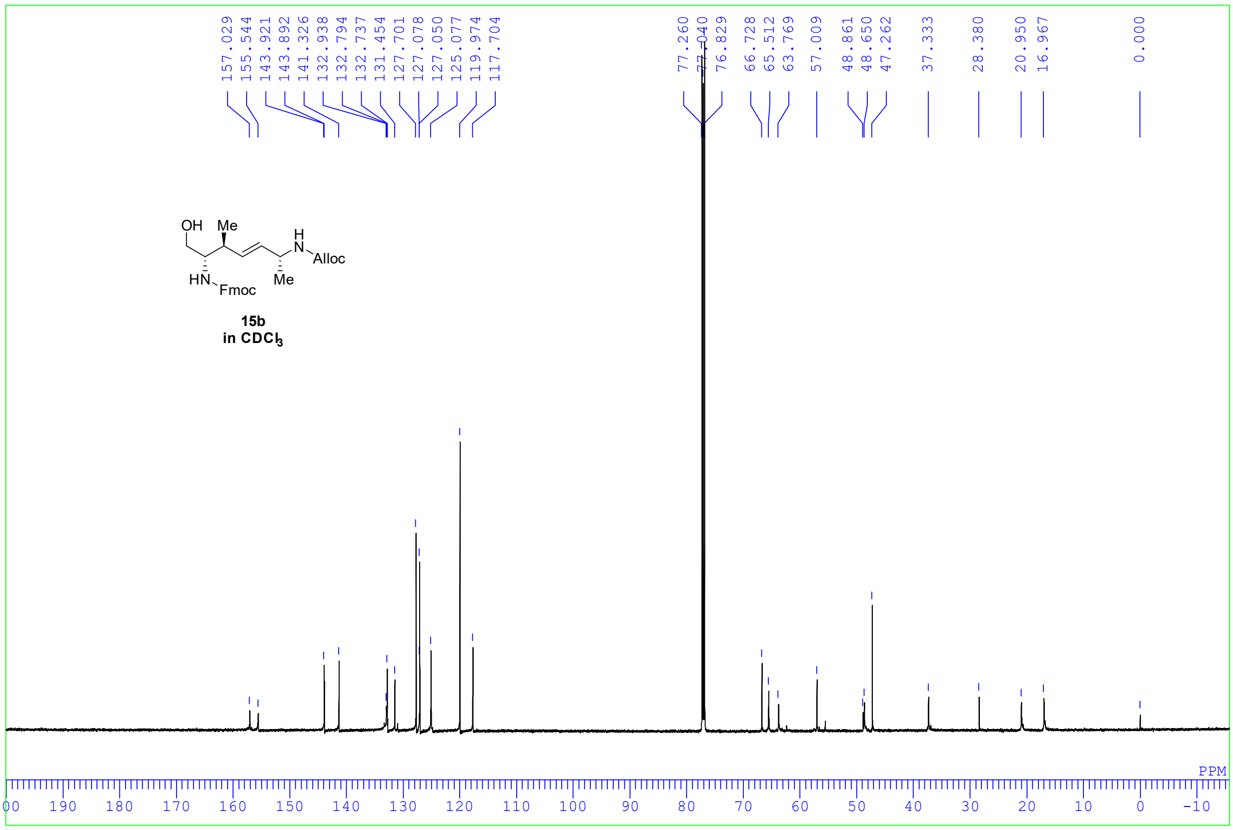
157.029 155.544 143.921 143.892 141.326 132.938 132.794 132.737 131.454 127.701 127.078 127.050 125.077 119.974 117.704
77.260 77.040 76.829 66.728 65.512 63.769 57.009 48.861 48.650 47.262 37.333 28.380 20.950 16.967 0.000
OH Me
H
N
Alloc
HN
Fmoc
Me
15b
in CDCl3
PPM

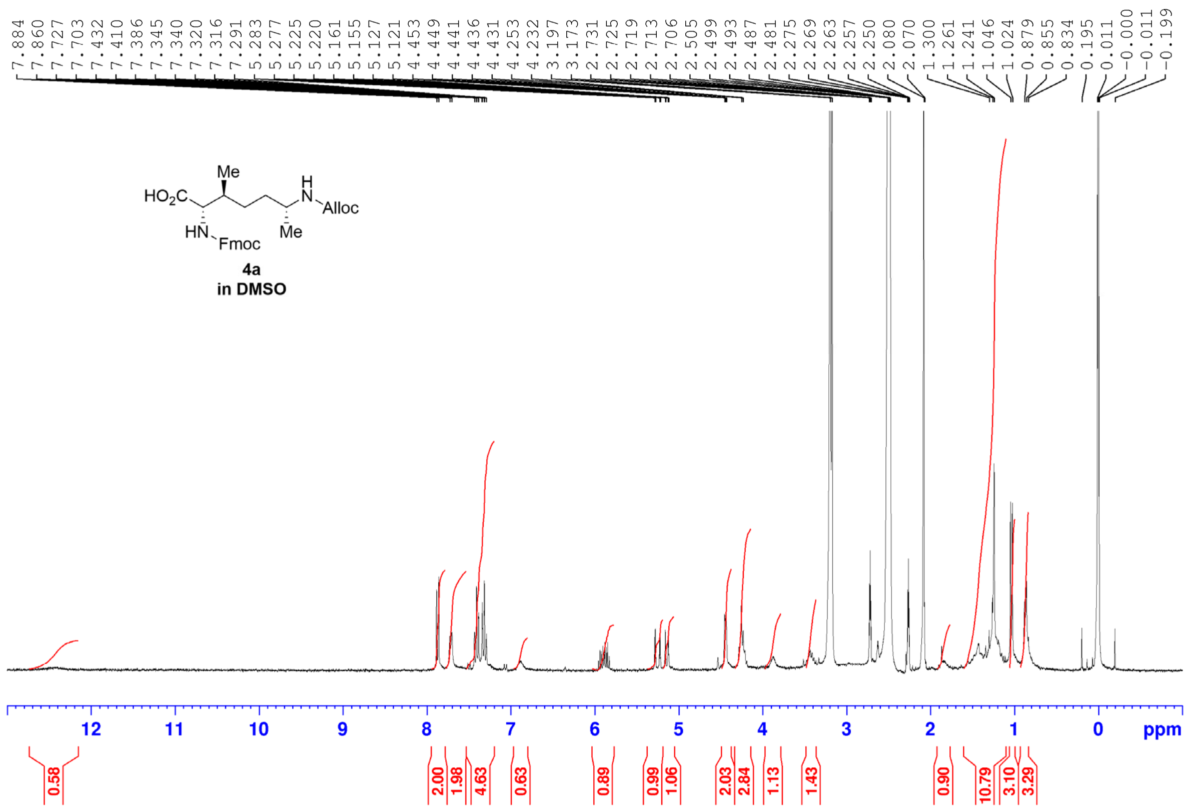

HO2C
Me
N
H
Alloc
HN
Fmoc
Me
4a
in DMSO
ppm

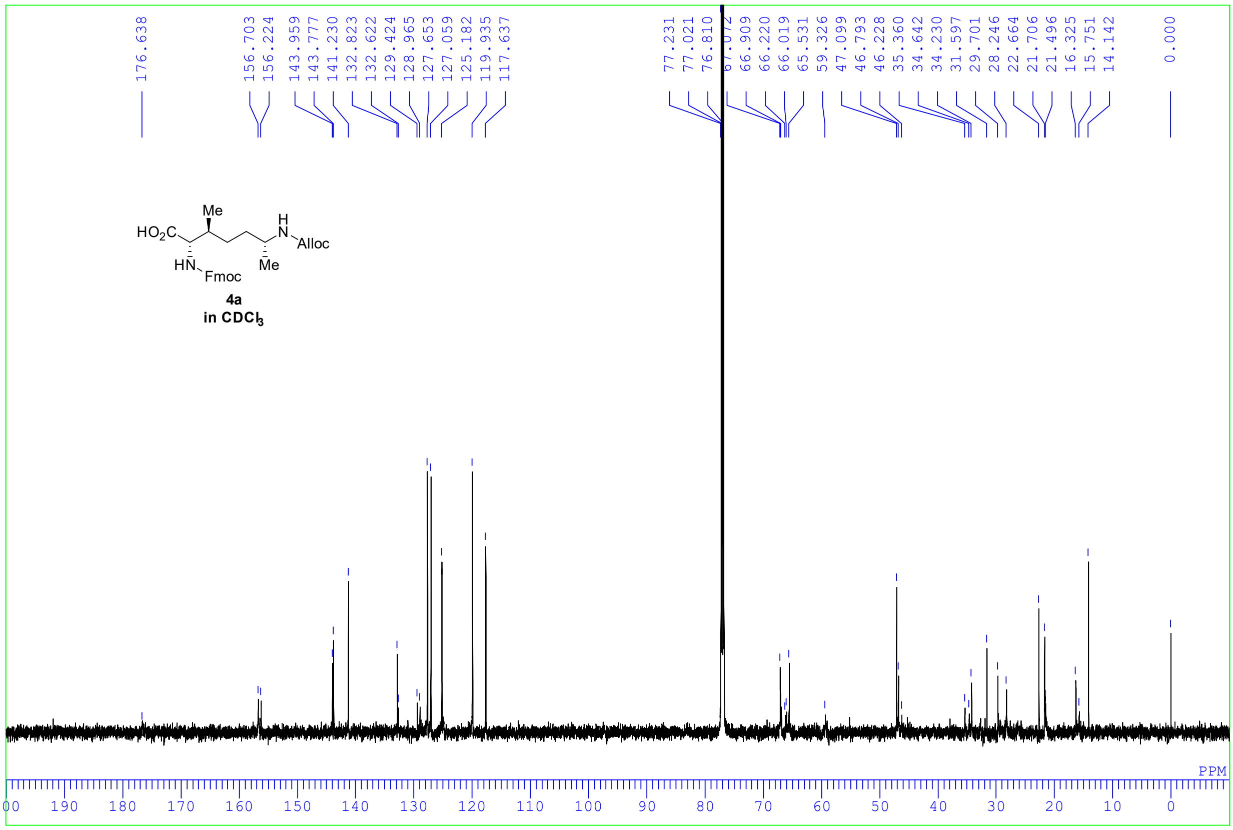

HO2C
Me
N
H
Alloc
HN
Fmoc
Me
4a
in CDCl3
PPM

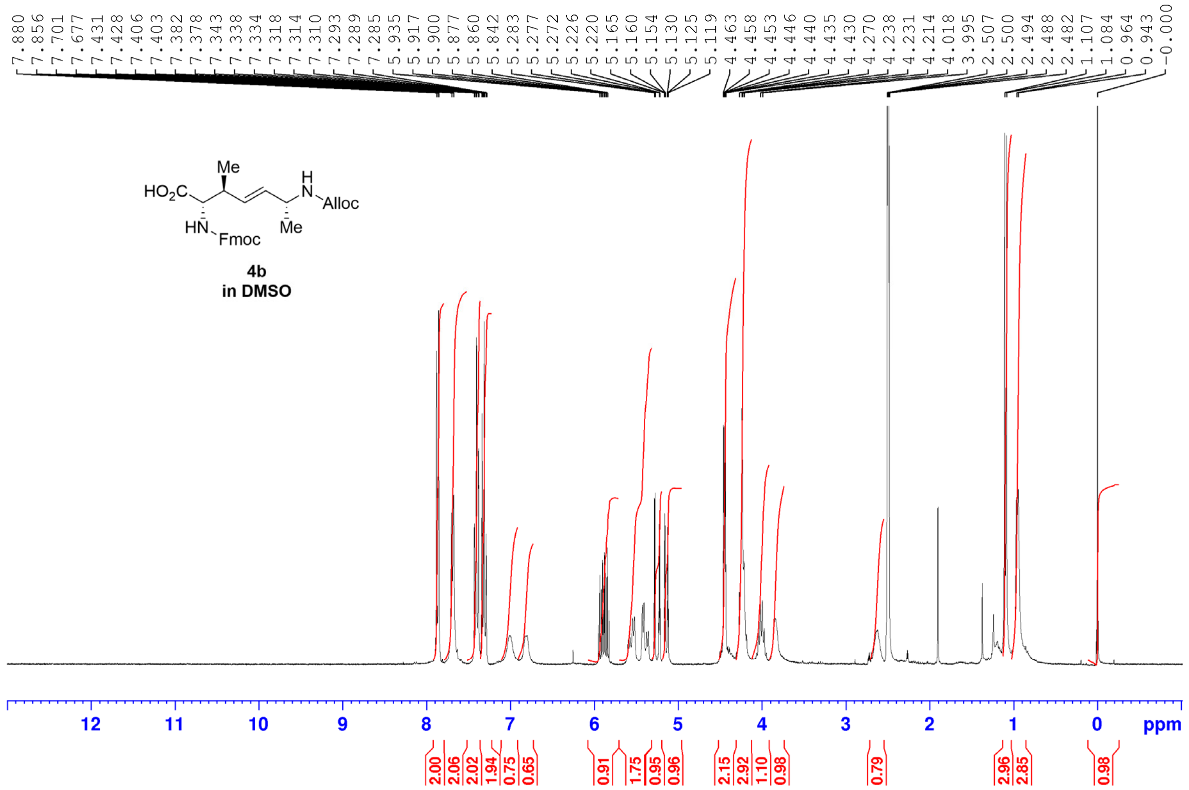
7.880
7.856
7.701
7.677
7.431
7.428
7.406
7.403
7.382
7.378
7.343
7.338
7.334
7.318
7.314
7.310
7.293
7.289
7.285
5.935
5.917
5.900
5.877
5.860
5.842
5.283
5.277
5.272
5.226
5.220
5.165
5.160
5.154
5.130
5.125
5.119
4.463
4.458
4.453
4.446
4.440
4.435
4.430
4.270
4.238
4.231
4.214
4.018
3.995
2.507
2.500
2.494
2.488
2.482
1.107
1.084
0.964
0.943
-0.000
Me
H
N
HO2C
Alloc
HN
Fmoc
Me
4b
in DMSO
12
11
10
9
8
7
6
5
4
3
2
1
0
ppm
2.00
2.06
2.02
1.94
0.75
0.65
0.91
1.75
0.95
0.96
2.15
2.92
1.10
0.98
0.79
2.96
2.85
0.98

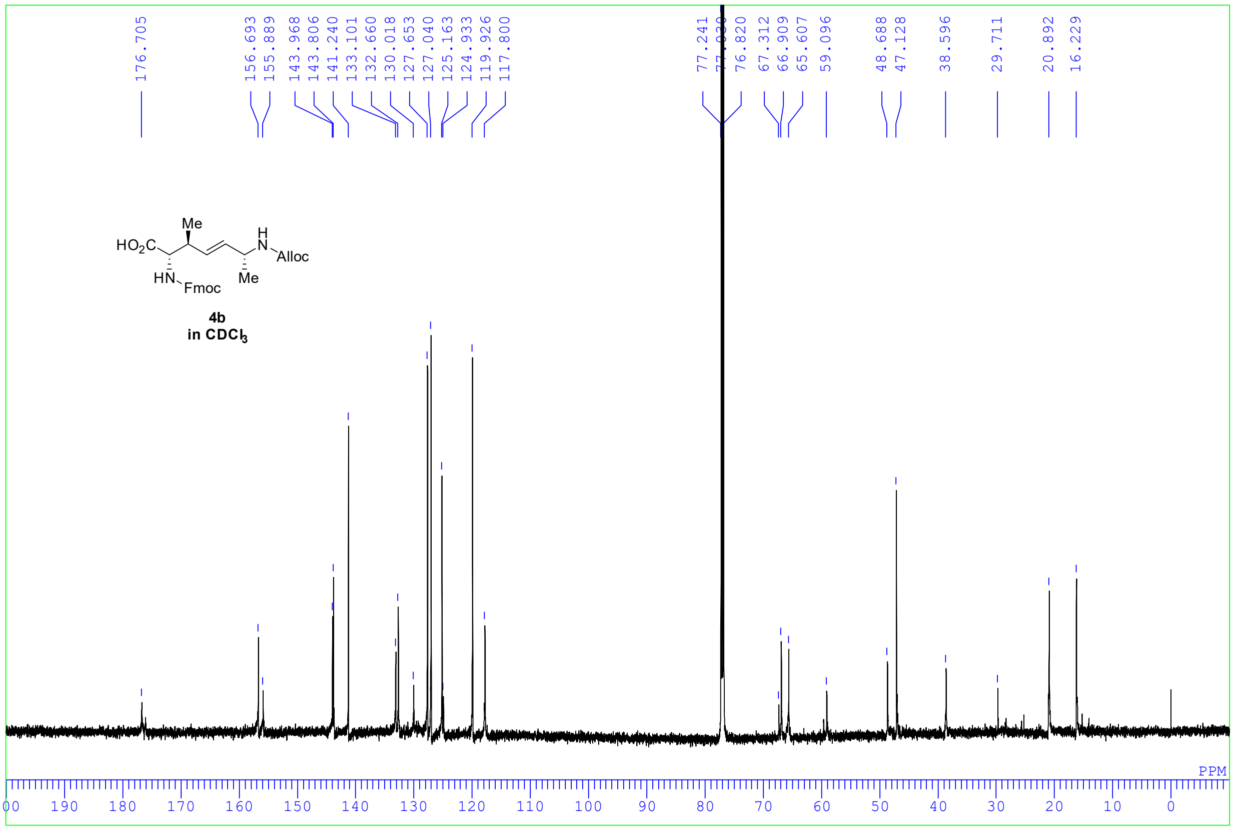
176.705
156.693
155.889
143.968
143.806
141.240
133.101
132.660
130.018
127.653
127.040
125.163
124.933
119.926
117.800
77.241
76.820
67.312
66.909
65.607
59.096
48.688
47.128
38.596
29.711
20.892
16.229
Me
H
N
HO2C
Alloc
HN
Fmoc
Me
4b
in CDCl3
PPM
200
190
180
170
160
150
140
130
120
110
100
90
80
70
60
50
40
30
20
10
0

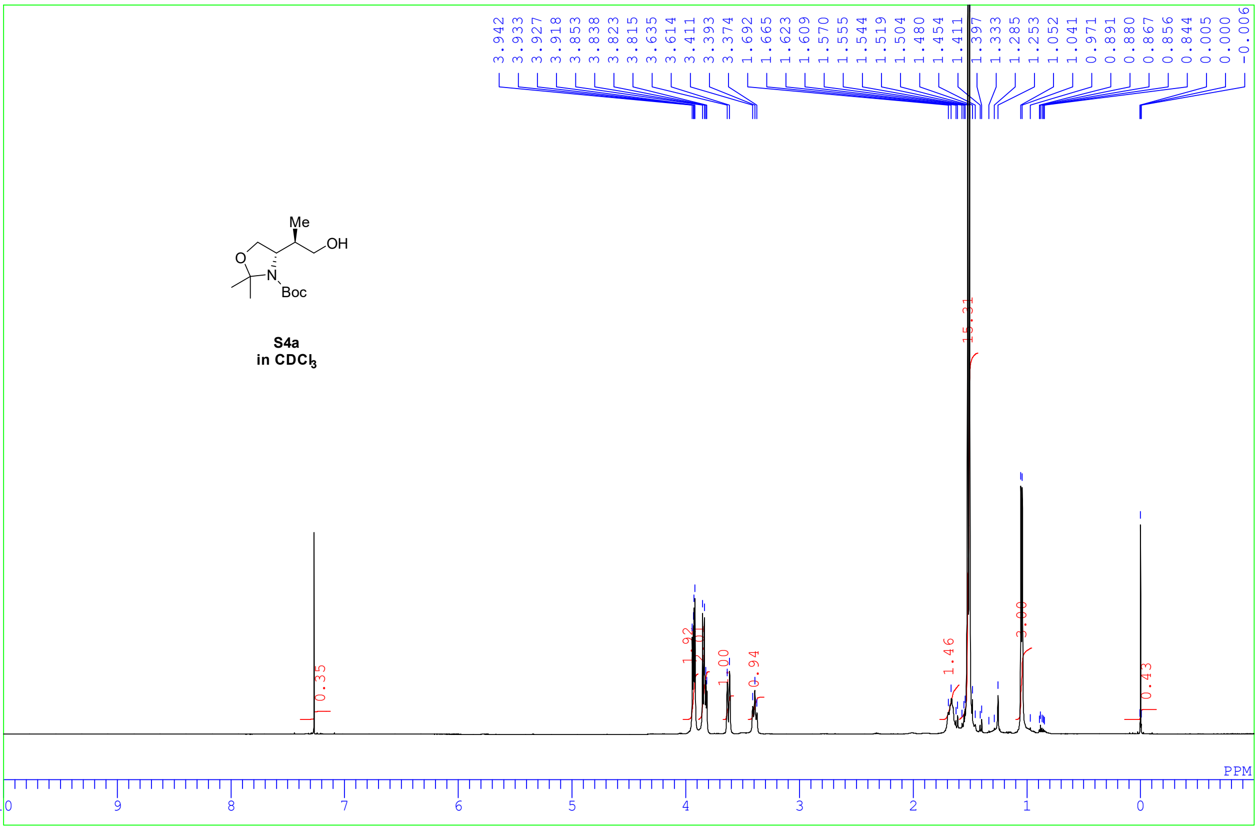
Me
OH
O
N
Boc
S4a
in CDCl3
PPM

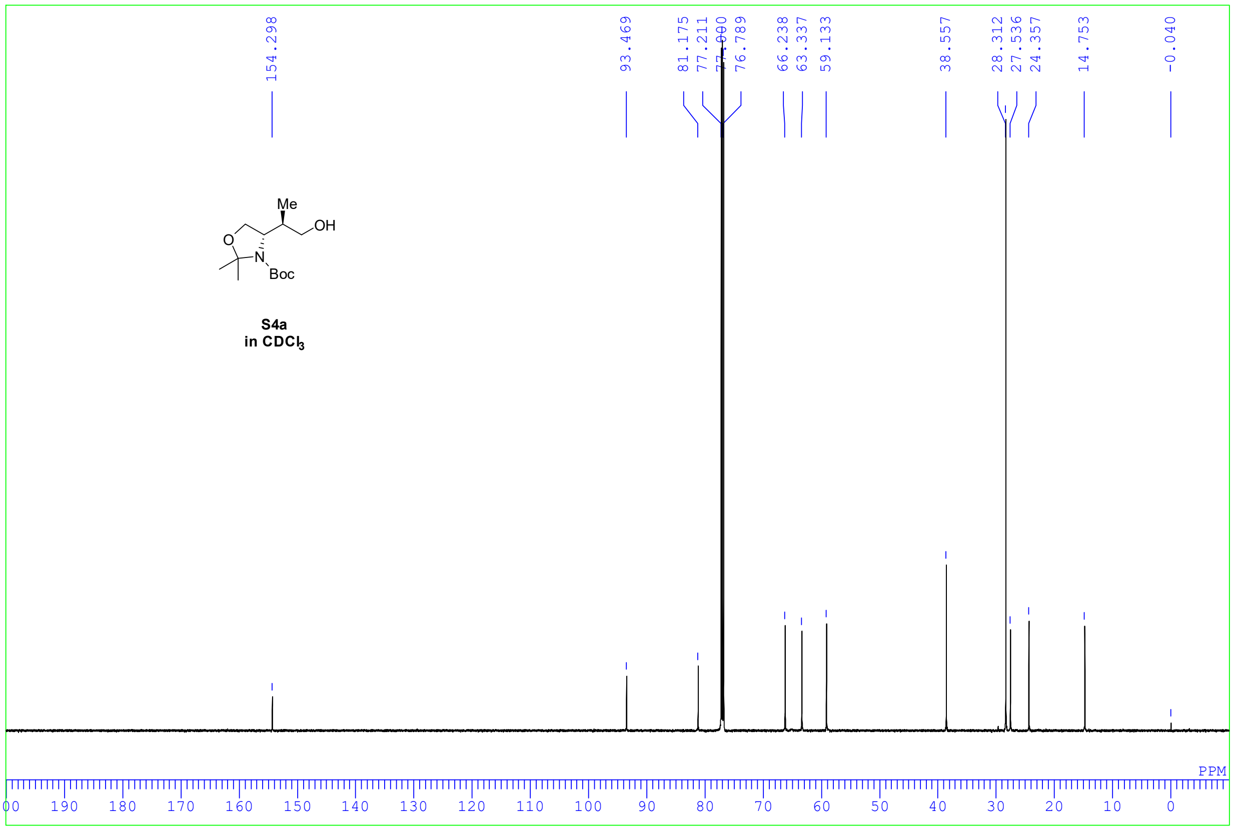
154.298
93.469
81.175
77.211
76.789
66.238
63.337
59.133
38.557
28.312
27.536
24.357
14.753
-0.040
Me
OH
O
N
Boc
S4a
in CDCl3
PPM

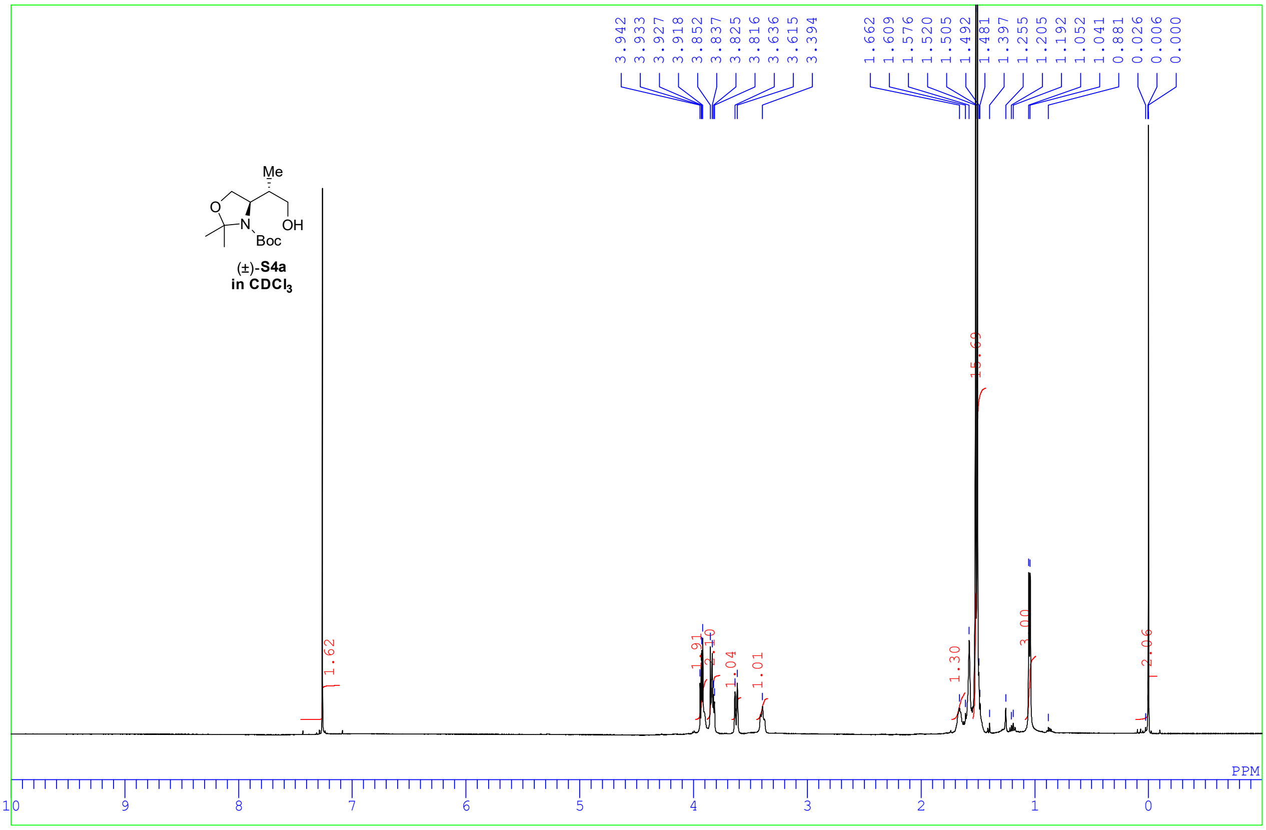
Me
O
N
OH
Boc
(±)-S4a
in CDCl3
3.942 3.933 3.927 3.918 3.852 3.837 3.825 3.816 3.636 3.615 3.394
1.662 1.609 1.576 1.520 1.505 1.492 1.481 1.397 1.255 1.205 1.192 1.052 1.041 0.881 0.026 0.006 0.000
1.62 1.91 1.04 1.01 1.30 15.69 3.00 2.06
PPM
10 9 8 7 6 5 4 3 2 1 0

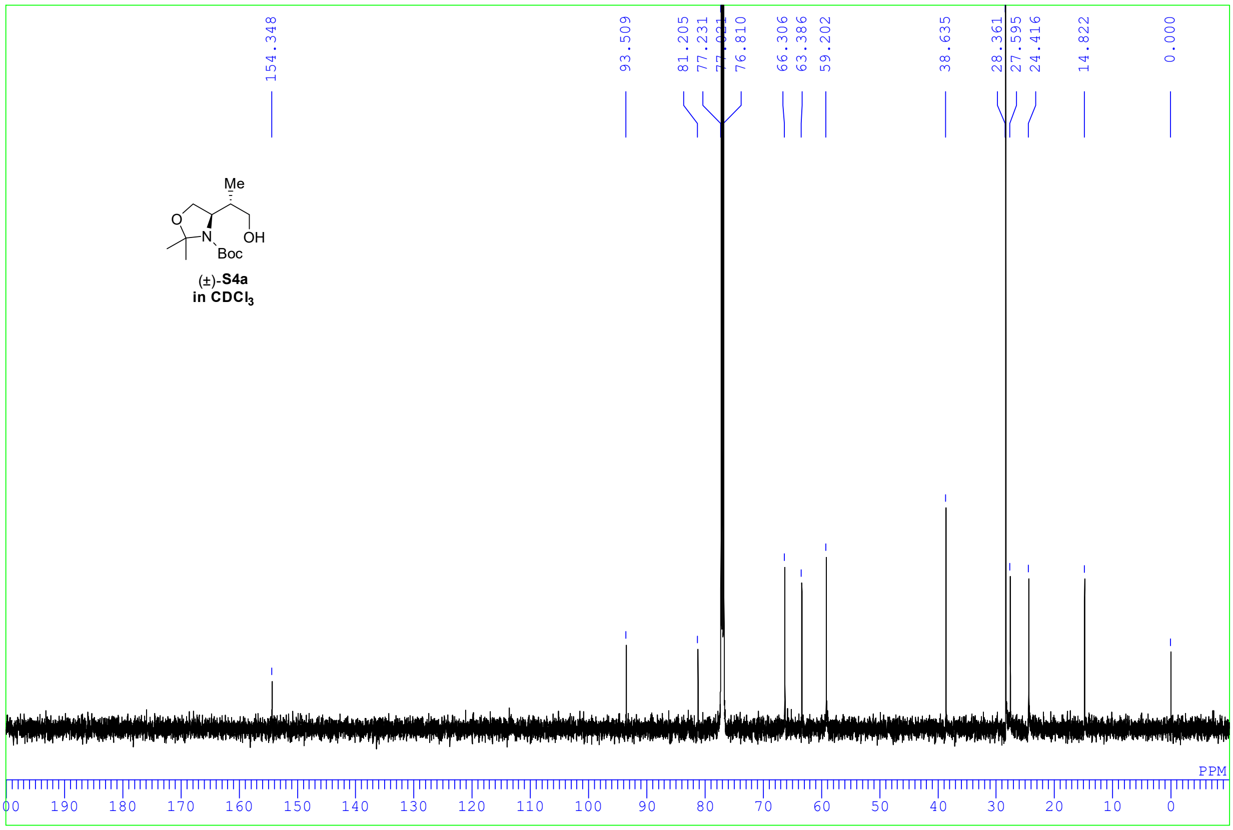
Me
O
N
OH
Boc
(±)-S4a
in CDCl3
154.348
93.509
81.205 77.231 76.810
66.306 63.386 59.202
38.635
28.361 27.595 24.416
14.822
0.000
PPM
200 190 180 170 160 150 140 130 120 110 100 90 80 70 60 50 40 30 20 10 0

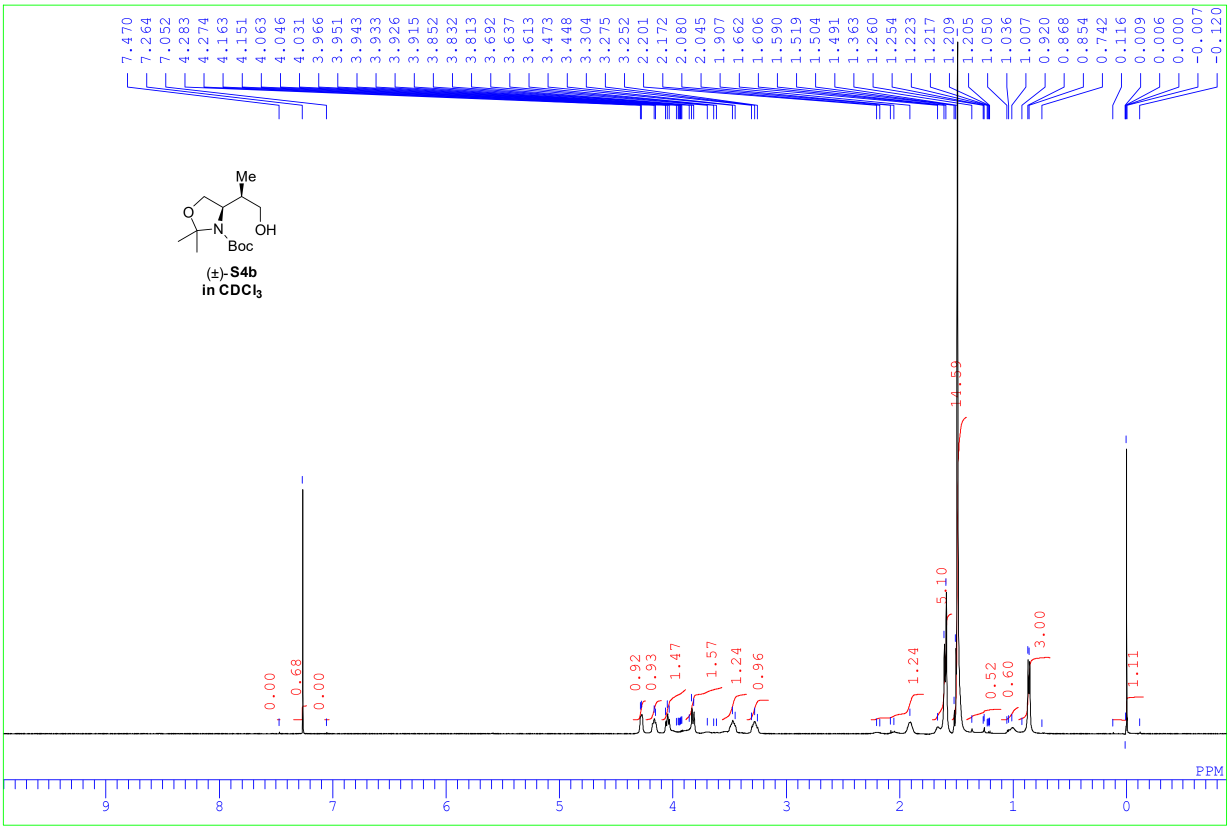

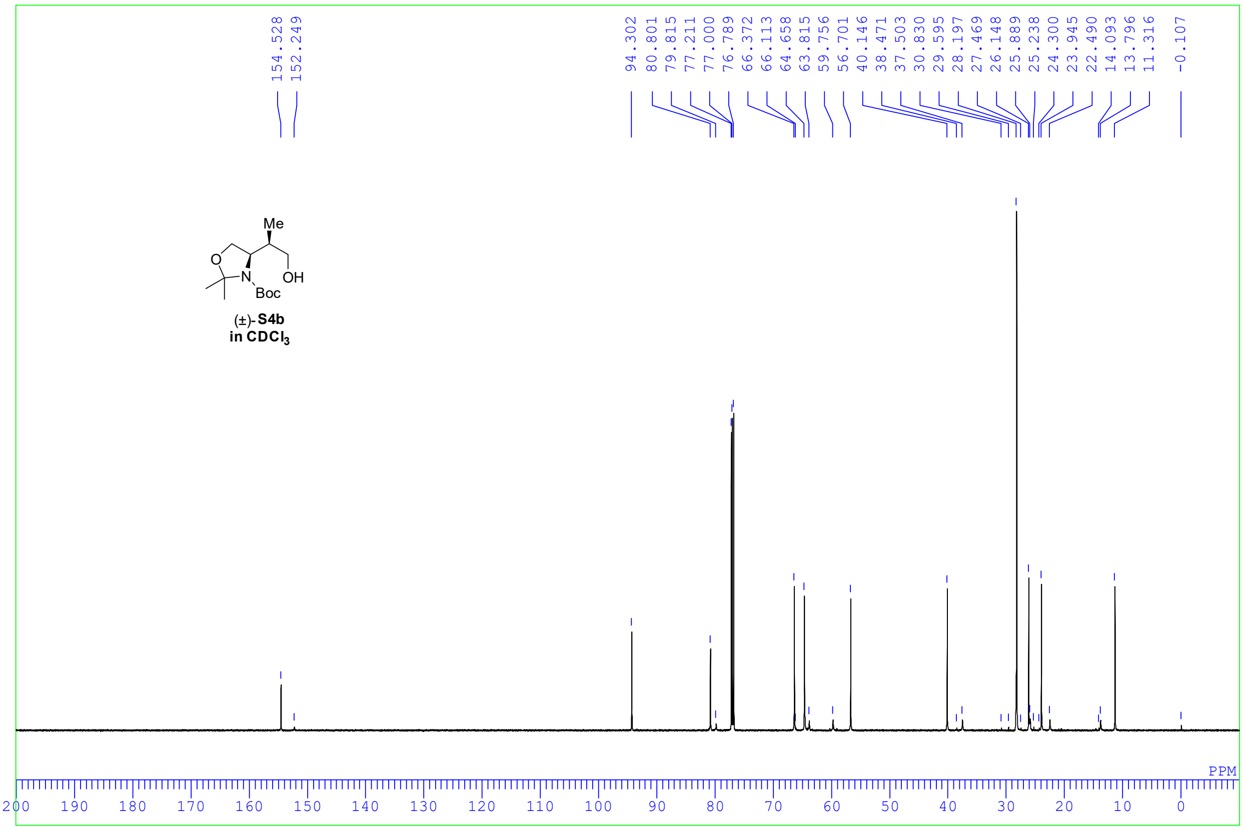

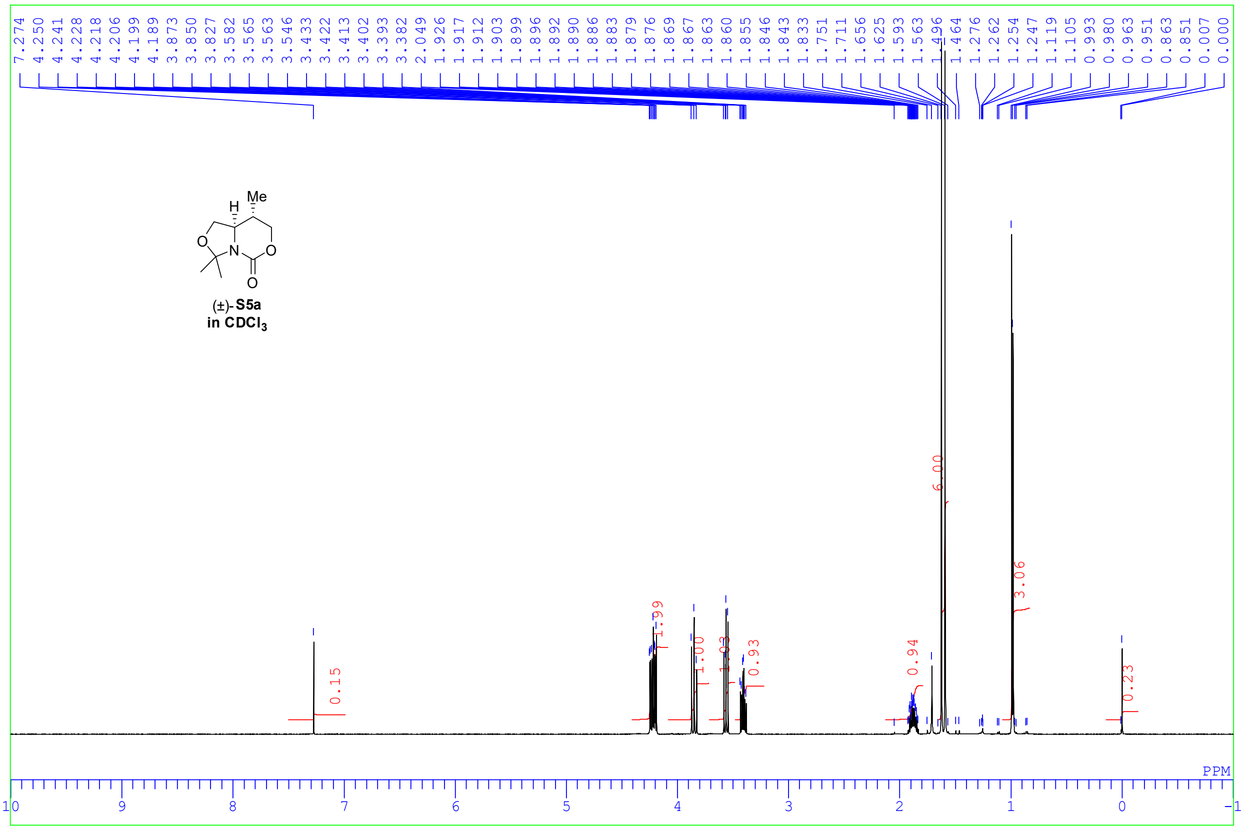

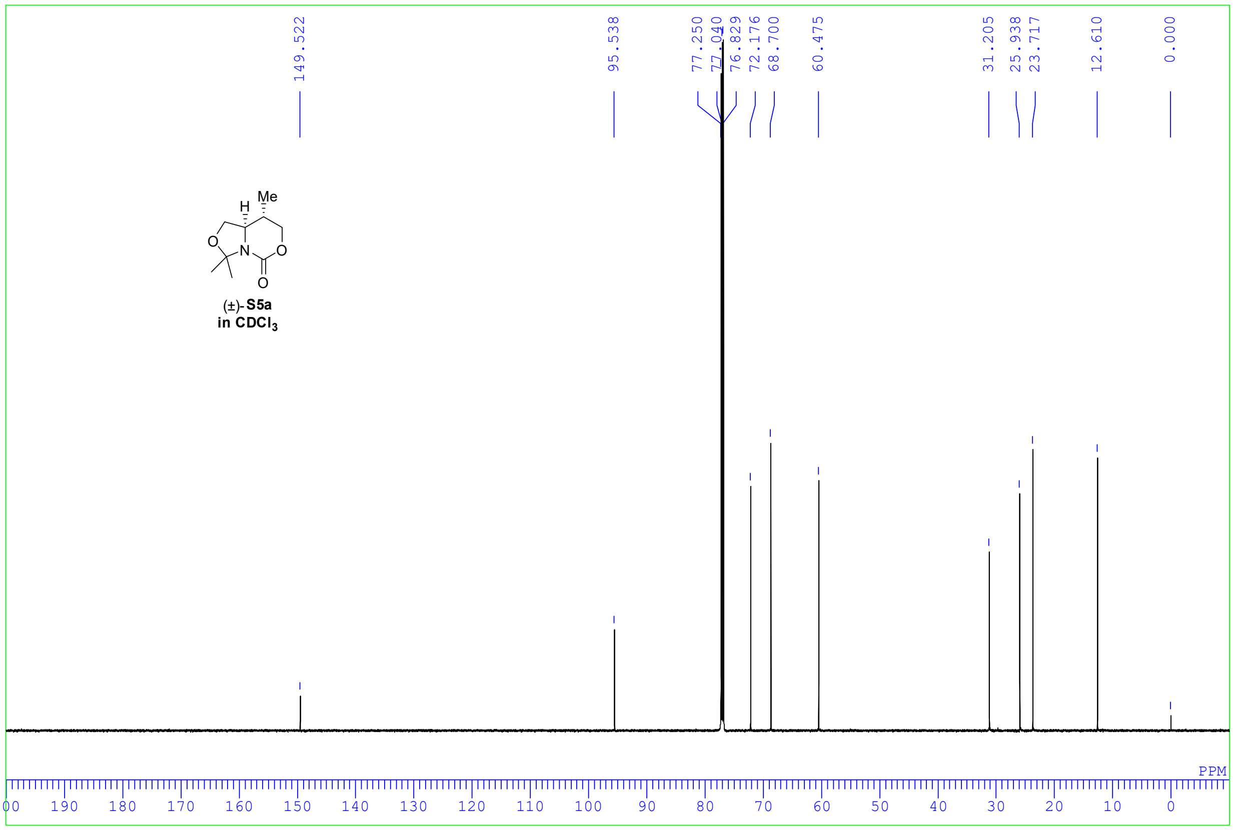

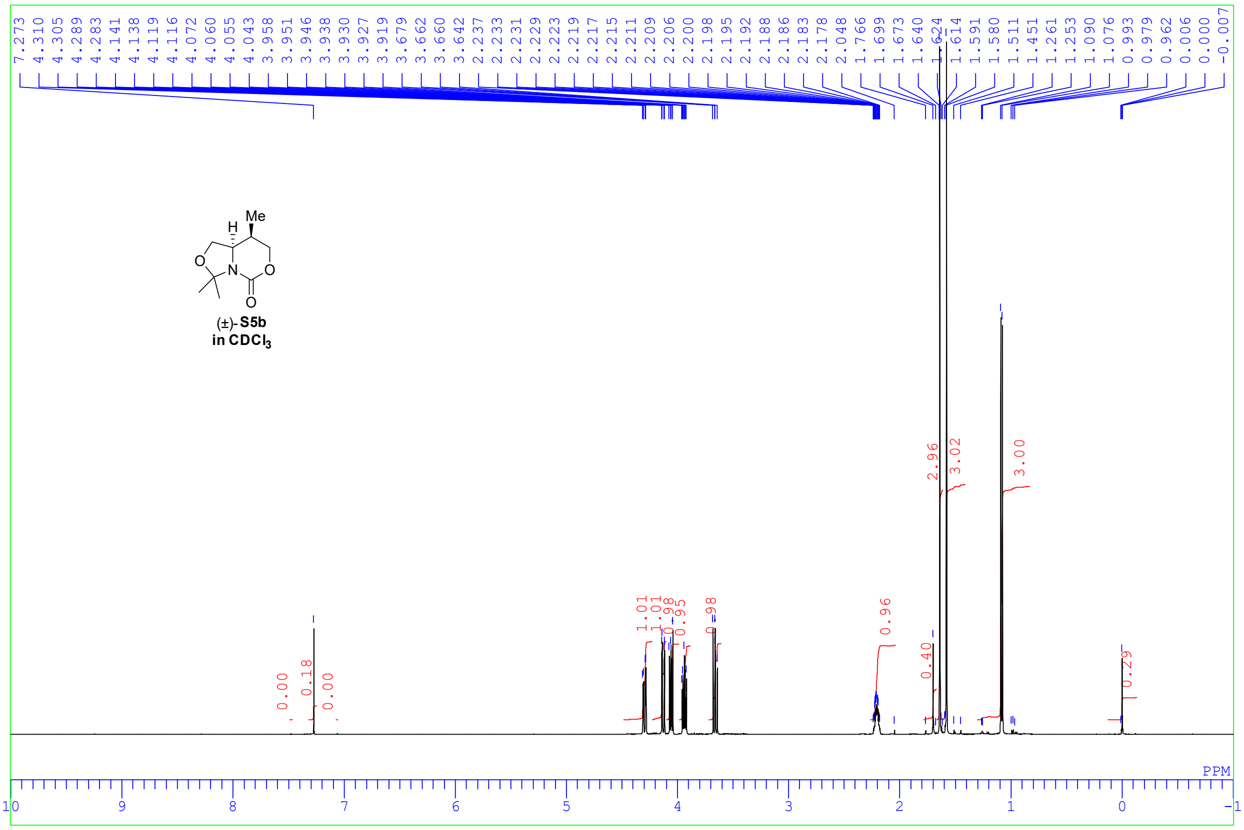

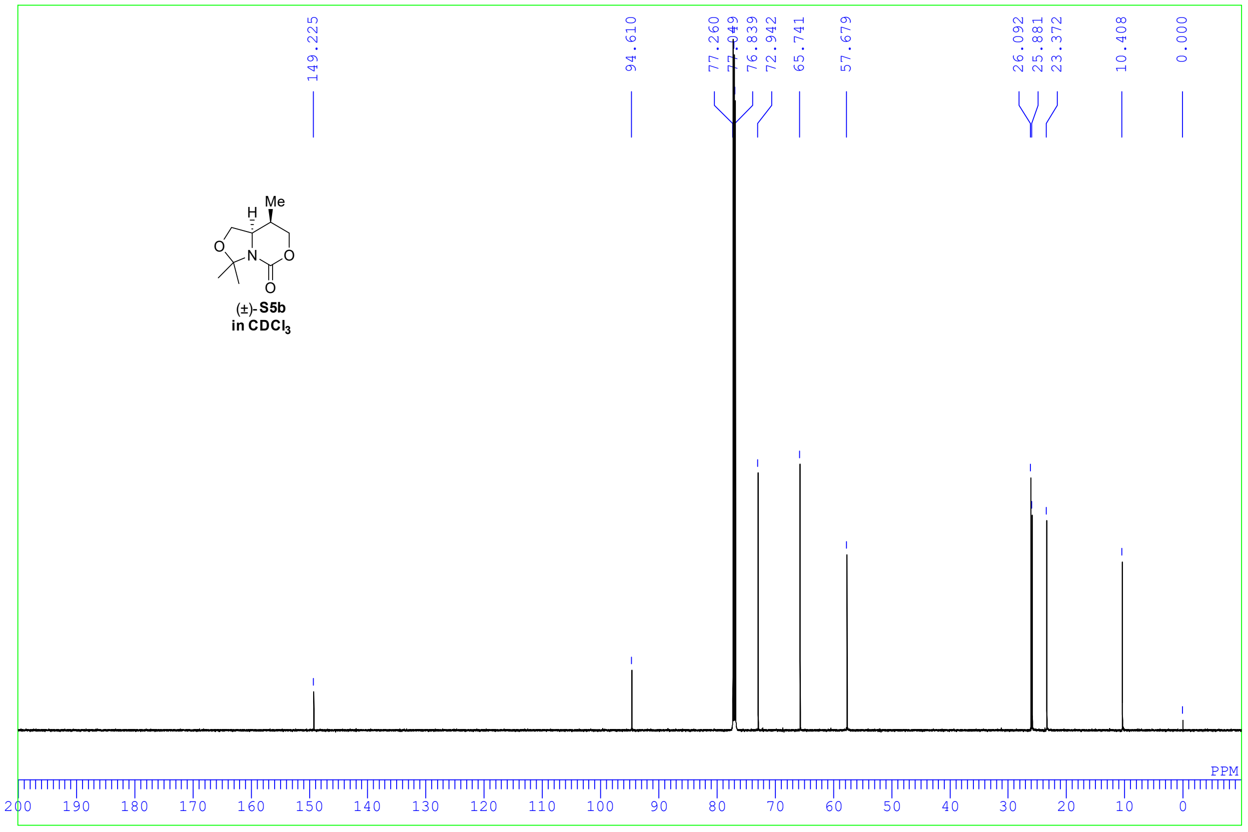

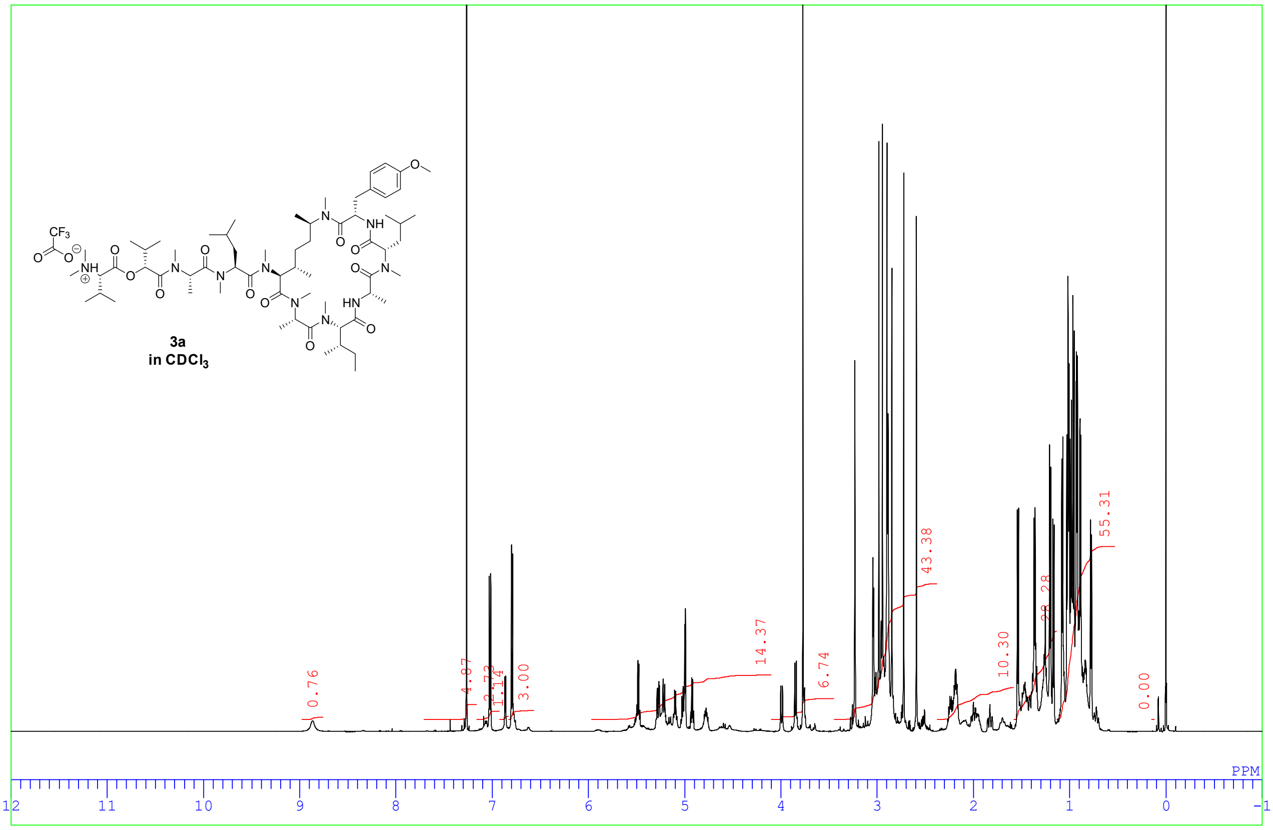

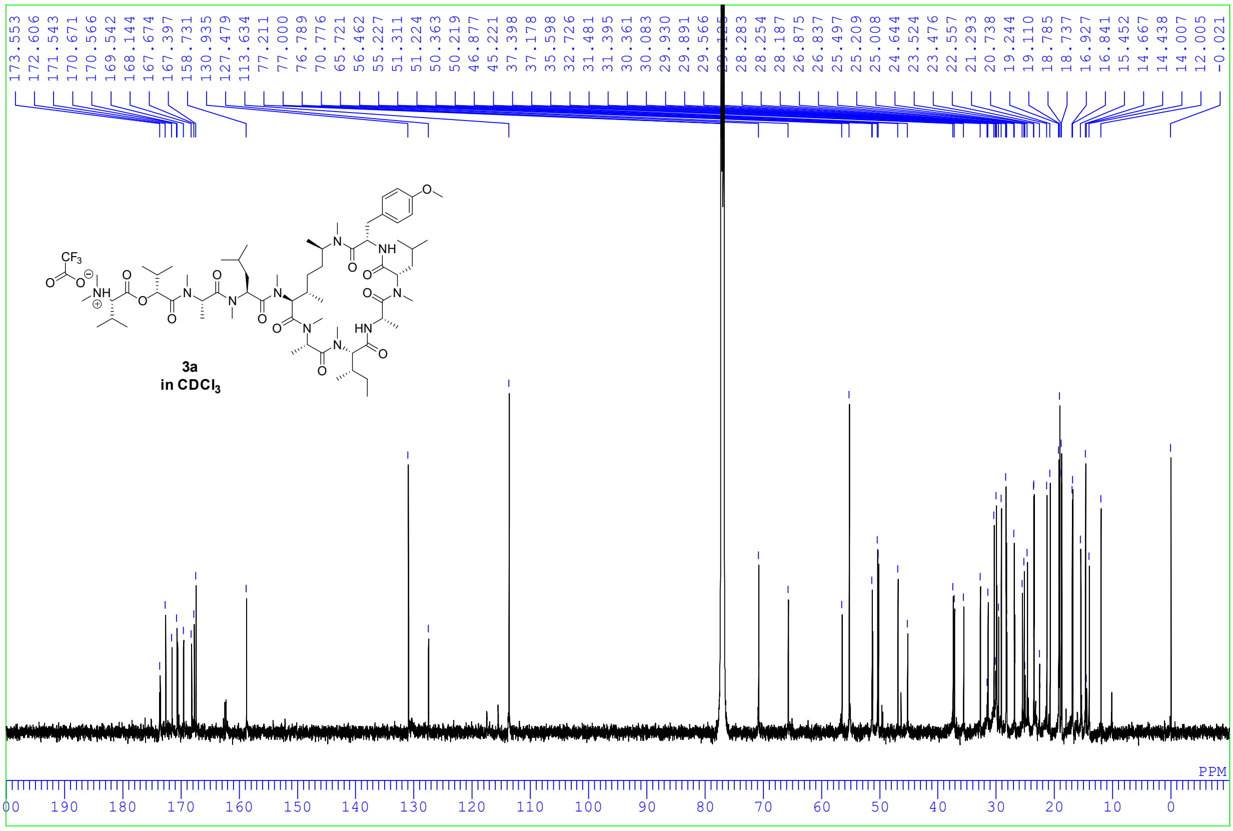

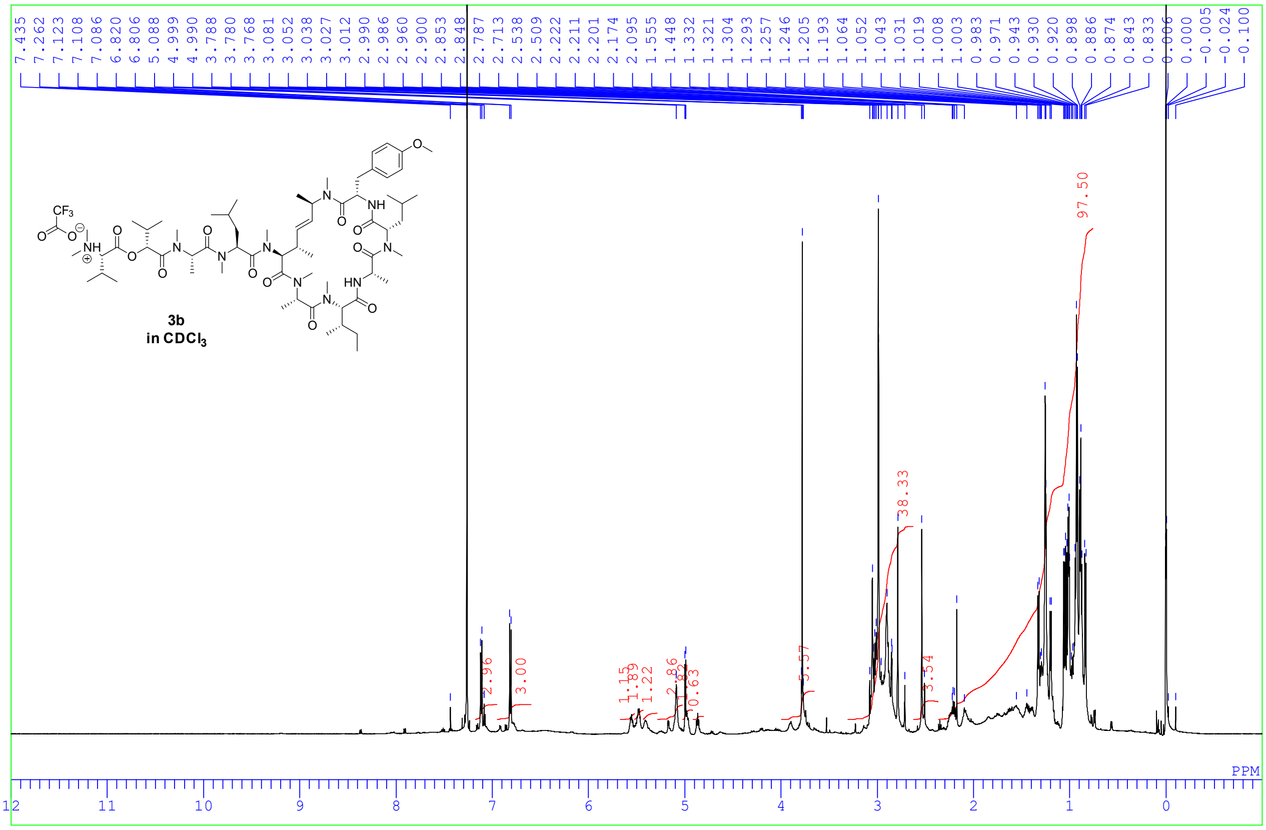
3b
in $CDCl_3$
PPM

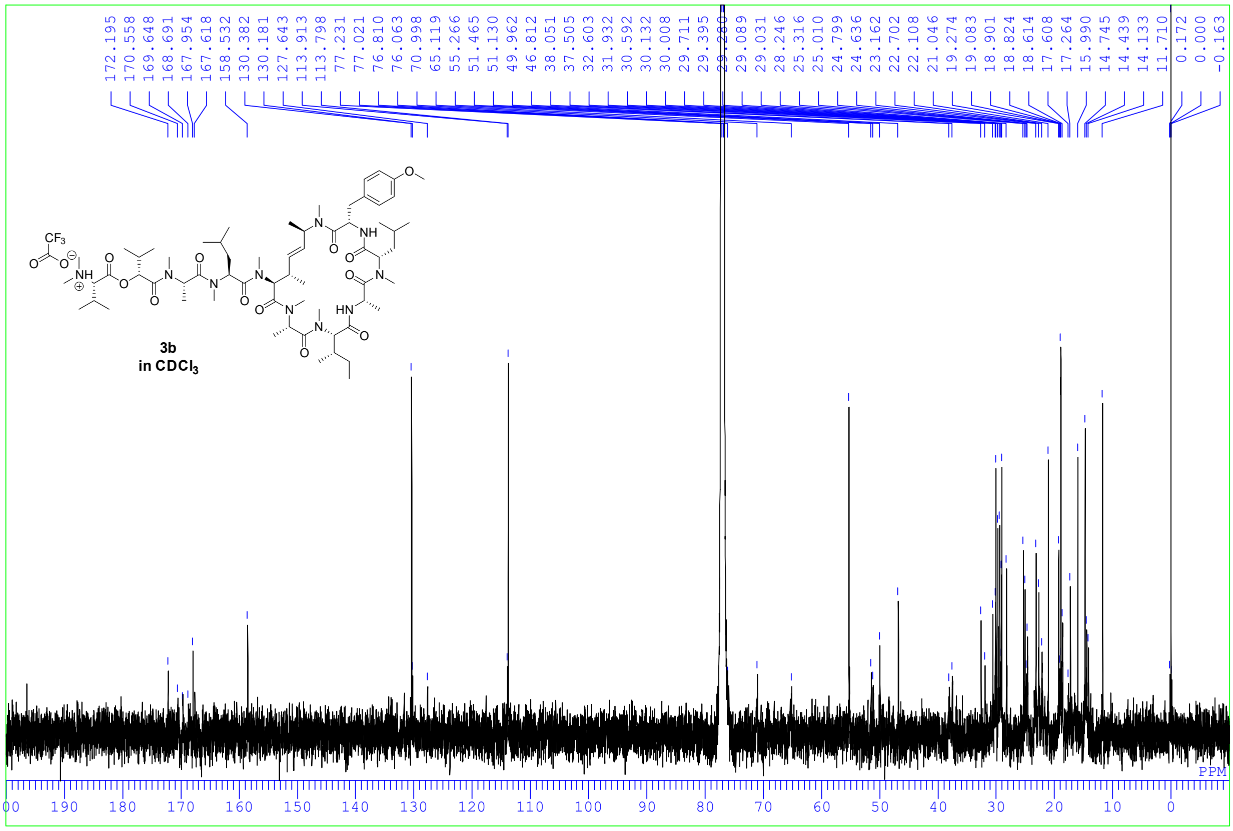
3b
in $CDCl_3$
PPM

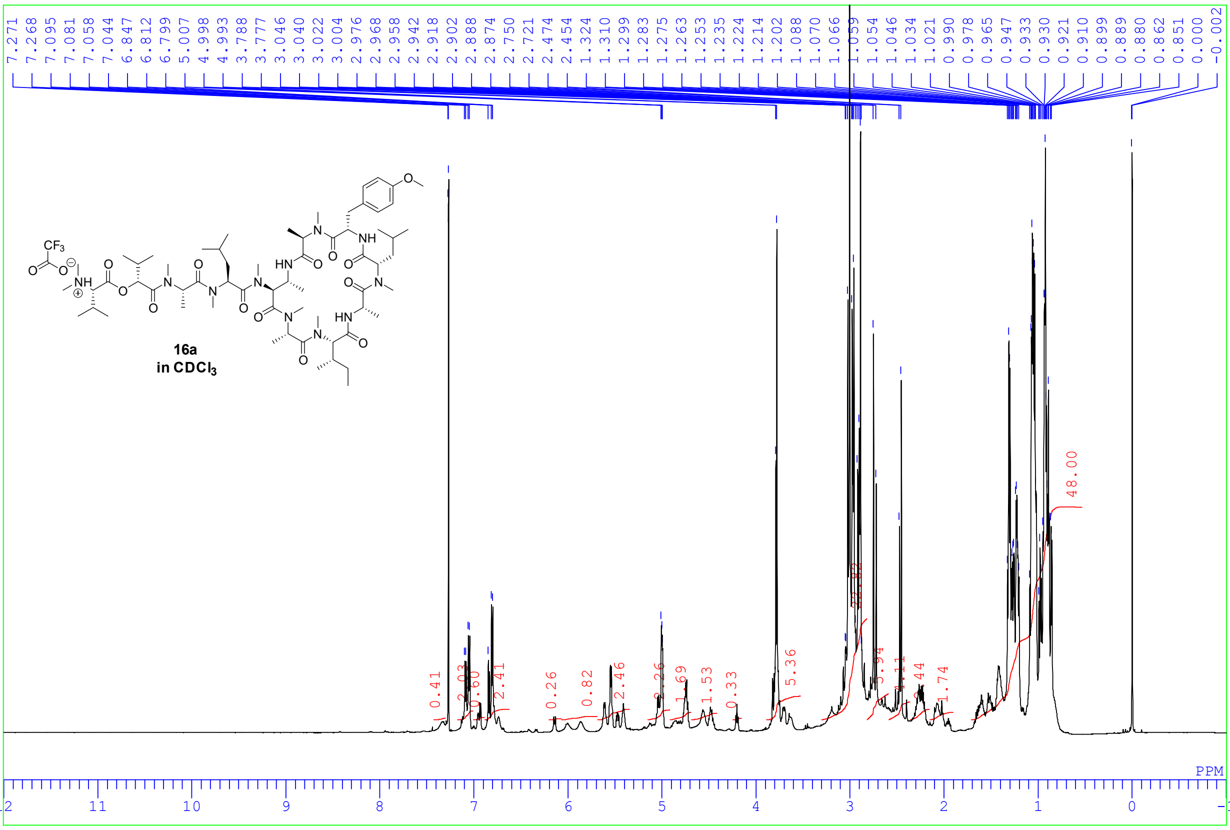
7.271 7.268 7.095 7.081 7.058 7.044 6.847 6.812 6.799 5.007 4.998 4.993 3.788 3.777 3.046 3.040 3.022 3.004 2.976 2.968 2.958 2.942 2.918 2.902 2.888 2.874 2.750 2.721 2.474 2.454 1.324 1.310 1.299 1.283 1.275 1.263 1.253 1.235 1.224 1.214 1.202 1.088 1.070 1.066 1.059 1.054 1.046 1.034 1.021 0.990 0.978 0.965 0.947 0.933 0.930 0.921 0.910 0.899 0.889 0.880 0.862 0.851 0.000 -0.002
16a
in CDCl3
PPM

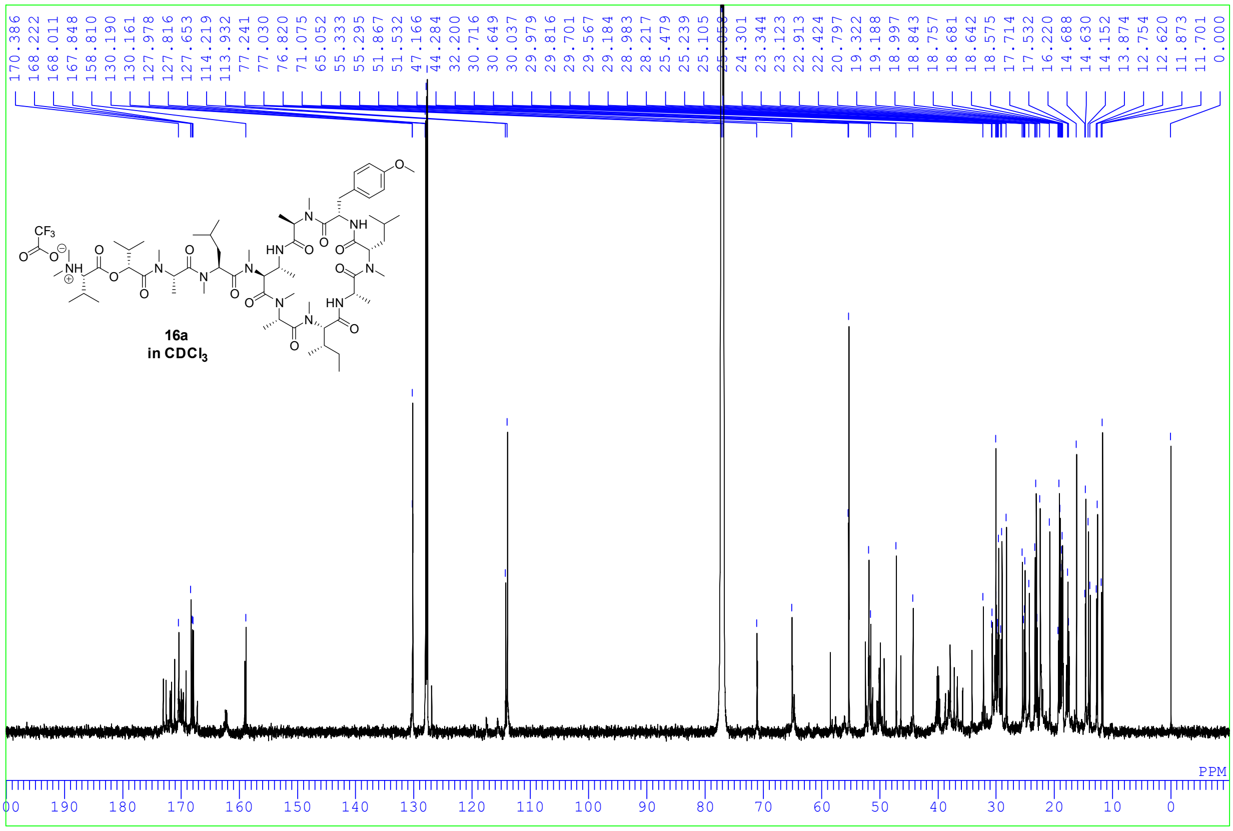
170.386 168.222 168.011 167.848 158.810 130.190 130.161 127.978 127.816 127.653 114.219 113.932 77.241 77.030 76.820 71.075 65.052 55.333 55.295 51.867 51.532 47.166 44.284 32.200 30.716 30.649 30.037 29.979 29.816 29.701 29.567 29.184 28.983 28.217 25.479 25.239 25.105 24.301 23.344 23.123 22.913 22.424 20.797 19.322 19.188 18.997 18.843 18.757 18.681 18.642 18.575 17.714 17.532 16.220 14.688 14.630 14.152 13.874 12.754 12.620 11.873 11.701 0.000
16a
in CDCl3
PPM

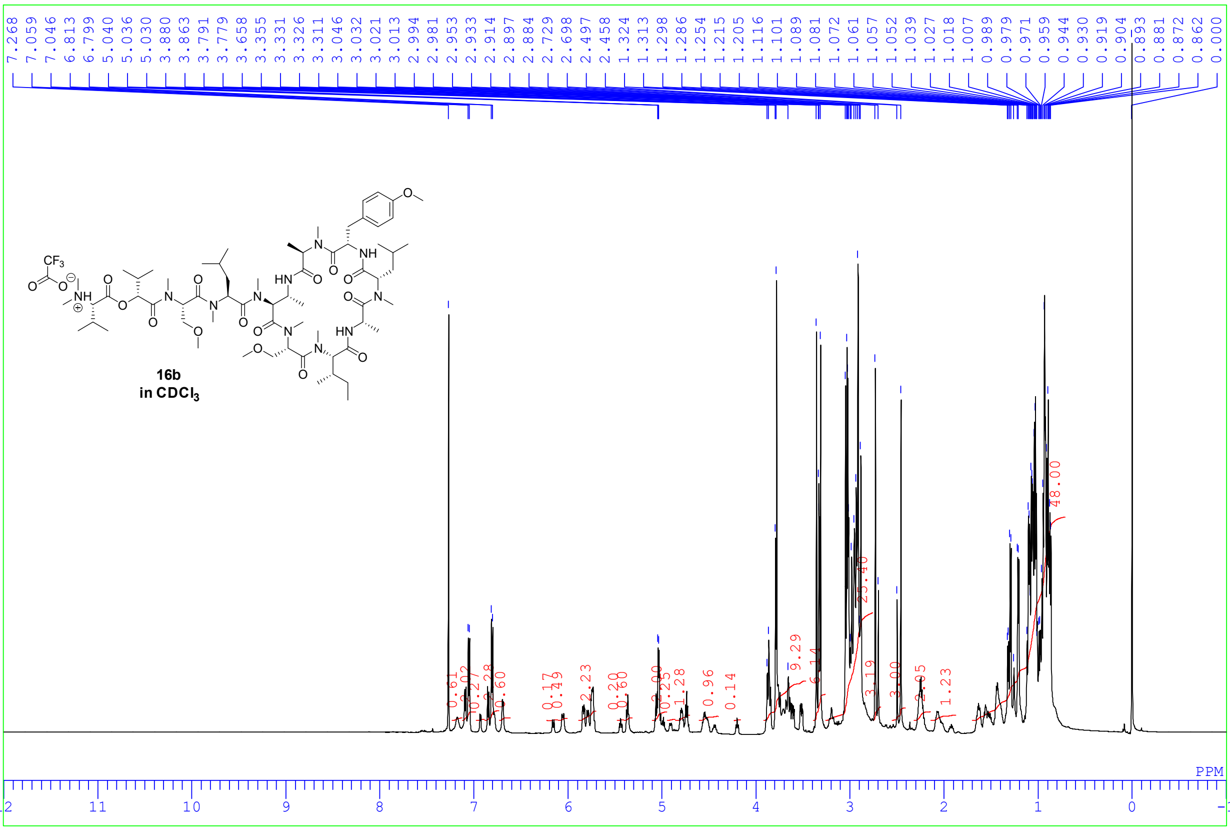
7.268 7.059 7.046 6.813 6.799 5.040 5.036 5.030 3.880 3.863 3.791 3.779 3.658 3.355 3.331 3.326 3.311 3.046 3.032 3.021 3.013 2.994 2.981 2.953 2.933 2.914 2.897 2.884 2.729 2.698 2.497 2.458 1.324 1.313 1.298 1.286 1.254 1.215 1.205 1.116 1.101 1.089 1.081 1.072 1.061 1.057 1.052 1.039 1.027 1.018 1.007 0.989 0.979 0.971 0.959 0.944 0.930 0.919 0.904 0.893 0.881 0.872 0.862 0.000
16b
in CDCl3
PPM

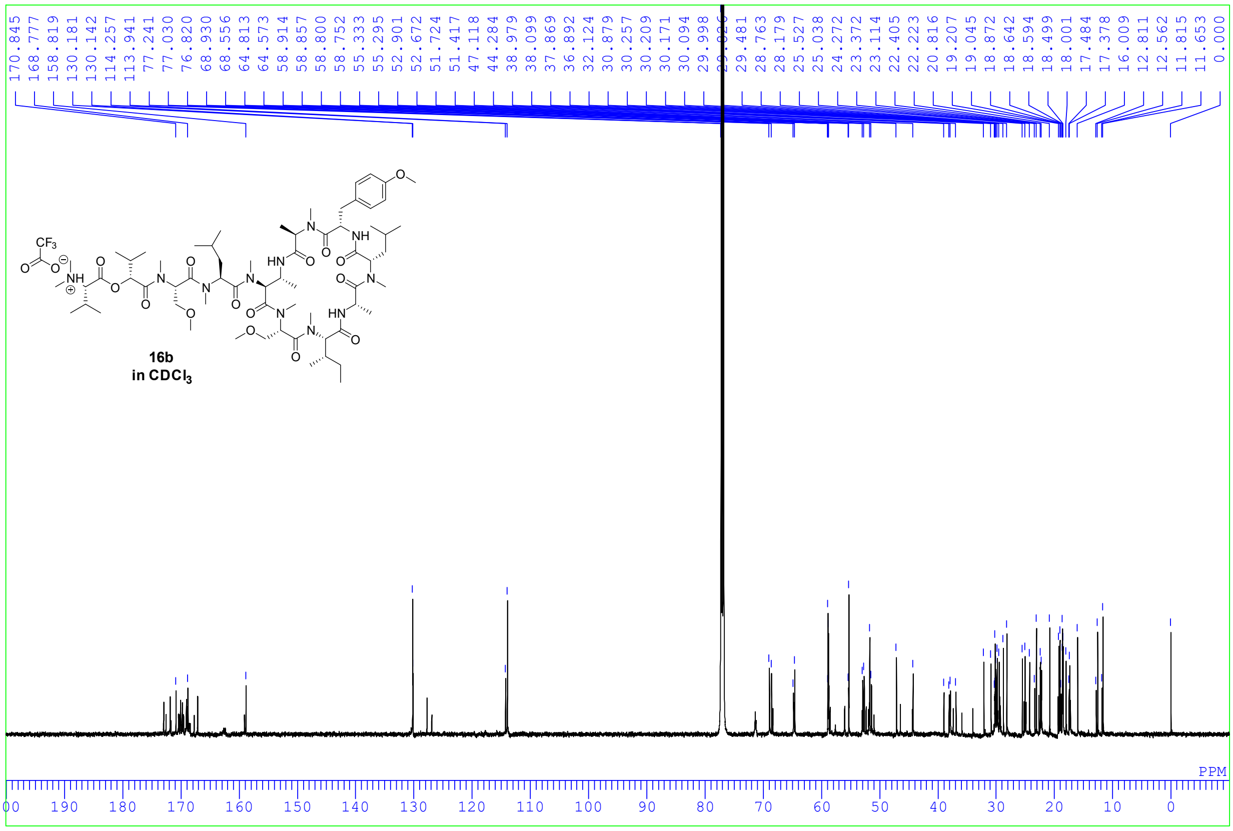
170.845 168.777 158.819 130.181 130.142 114.257 113.941 77.241 77.030 76.820 68.930 68.556 64.813 64.573 58.914 58.857 58.800 58.752 55.333 55.295 52.901 52.672 51.724 51.417 47.118 44.284 38.979 38.099 37.869 36.892 32.124 30.879 30.257 30.209 30.171 30.094 29.998 29.481 28.763 28.179 25.527 25.038 24.272 23.372 23.114 22.405 22.223 20.816 19.207 19.045 18.872 18.642 18.594 18.499 18.001 17.484 17.378 16.009 12.811 12.562 11.815 11.653 0.000
16b
in CDCl3
PPM

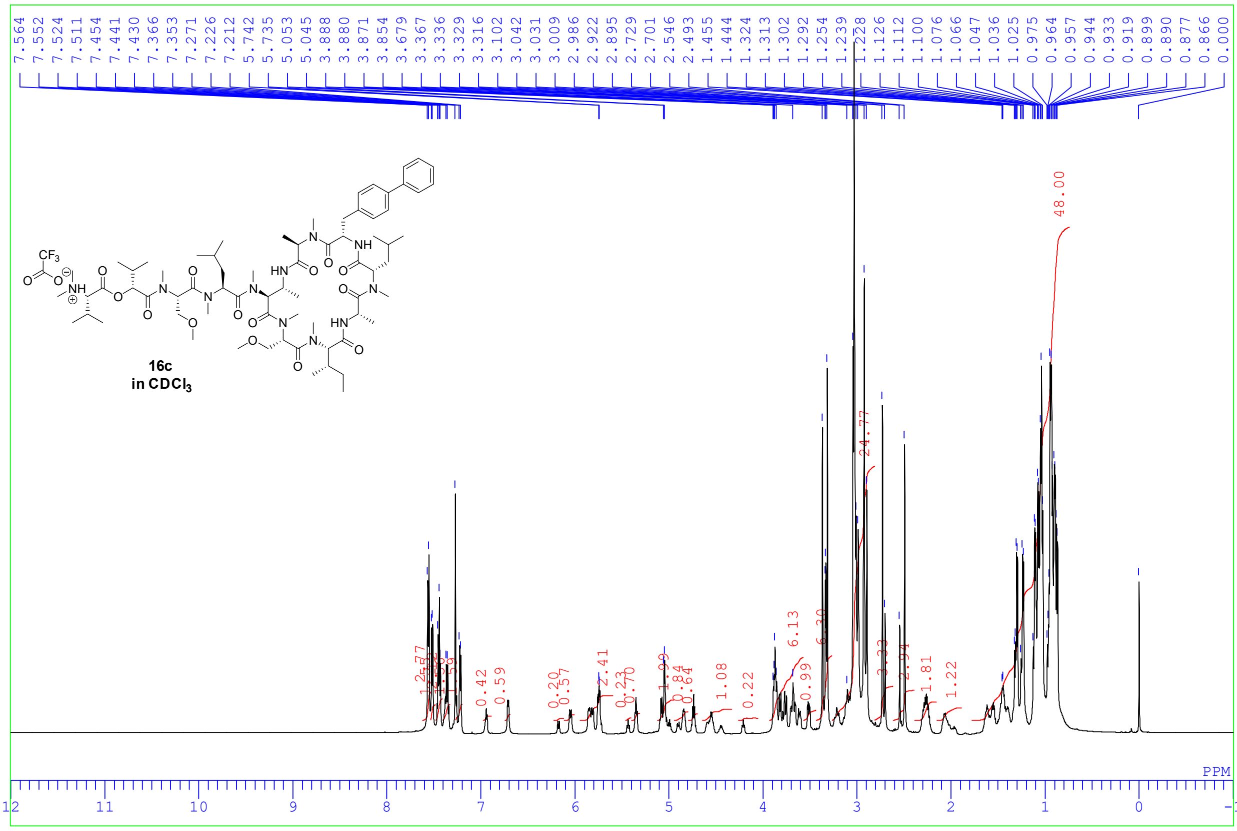

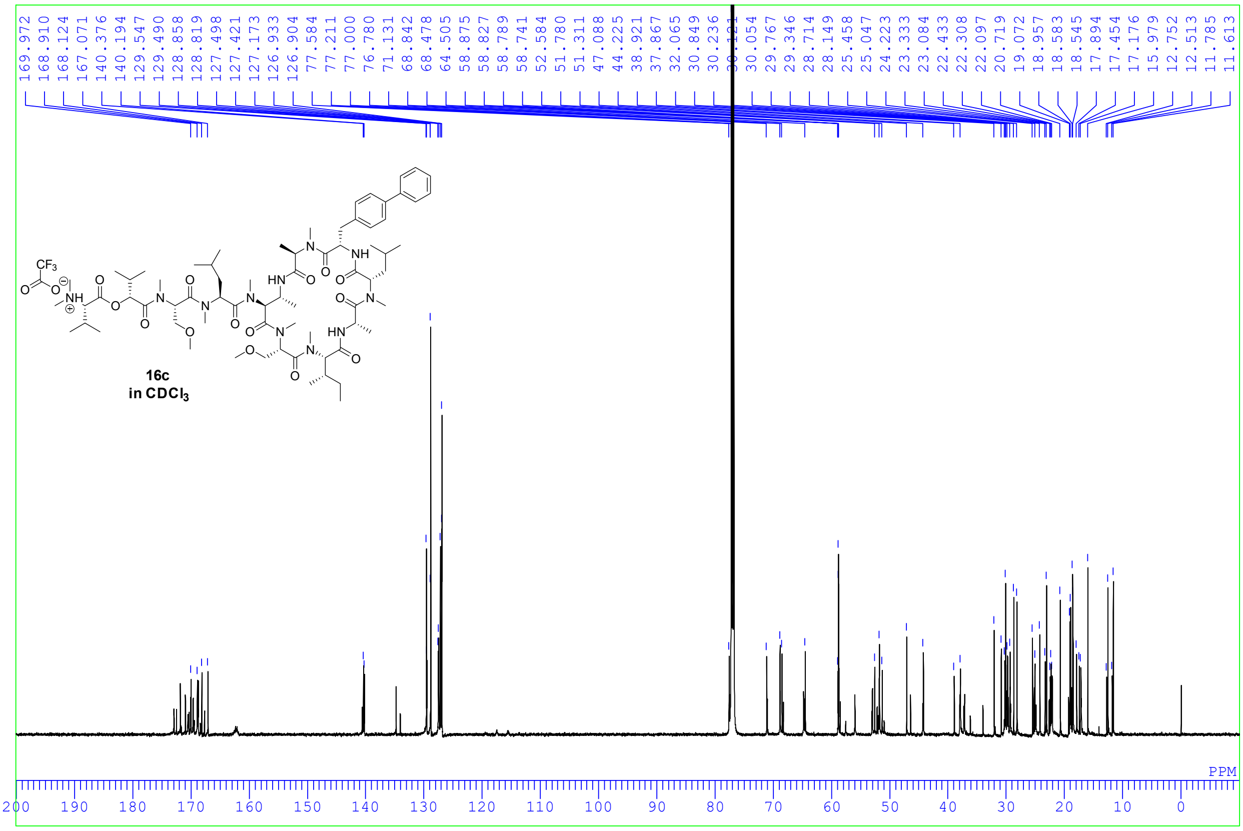

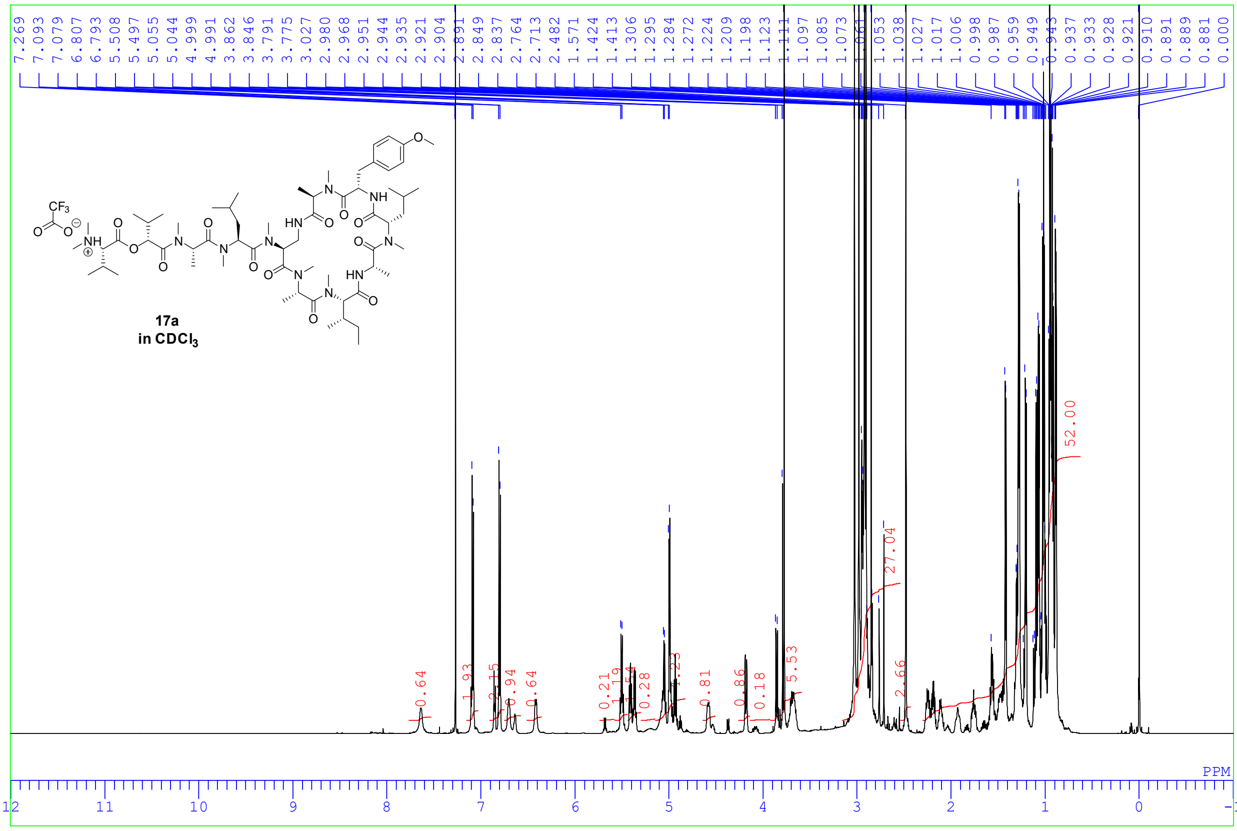

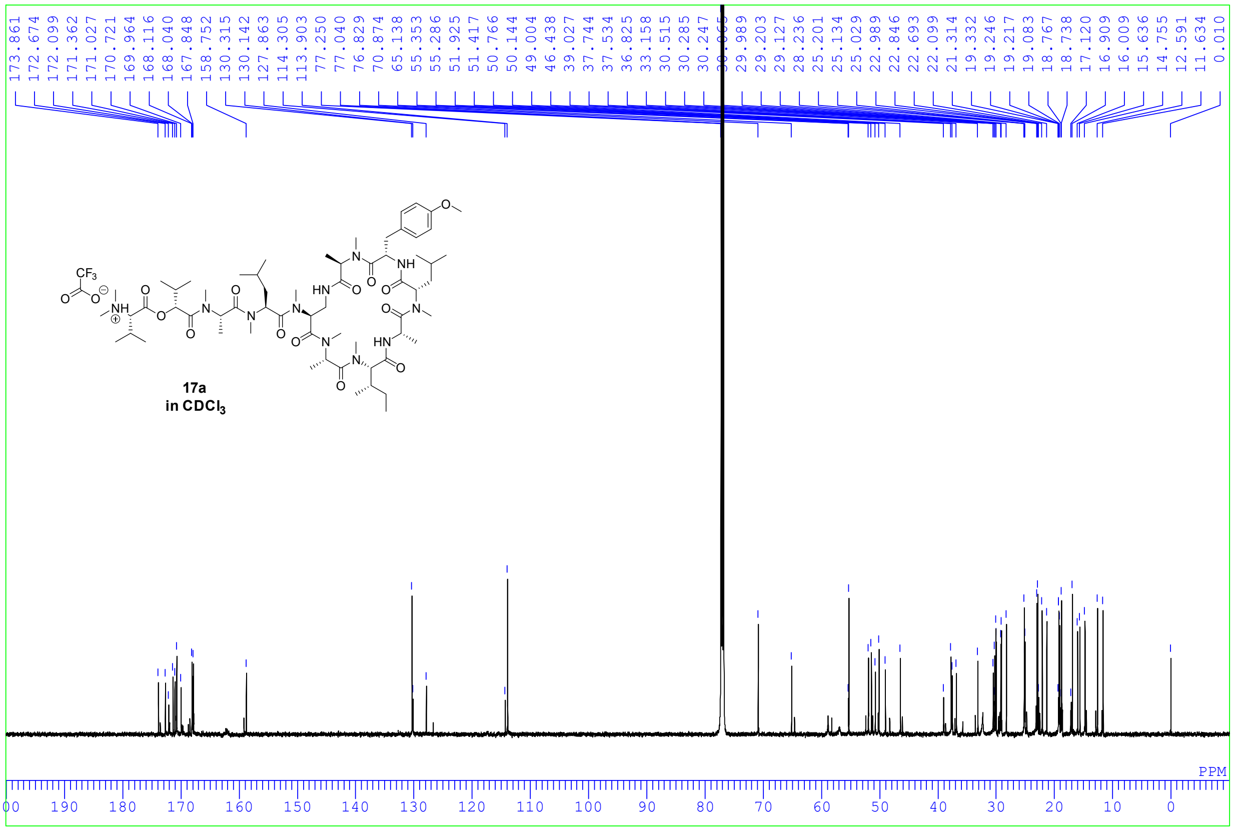

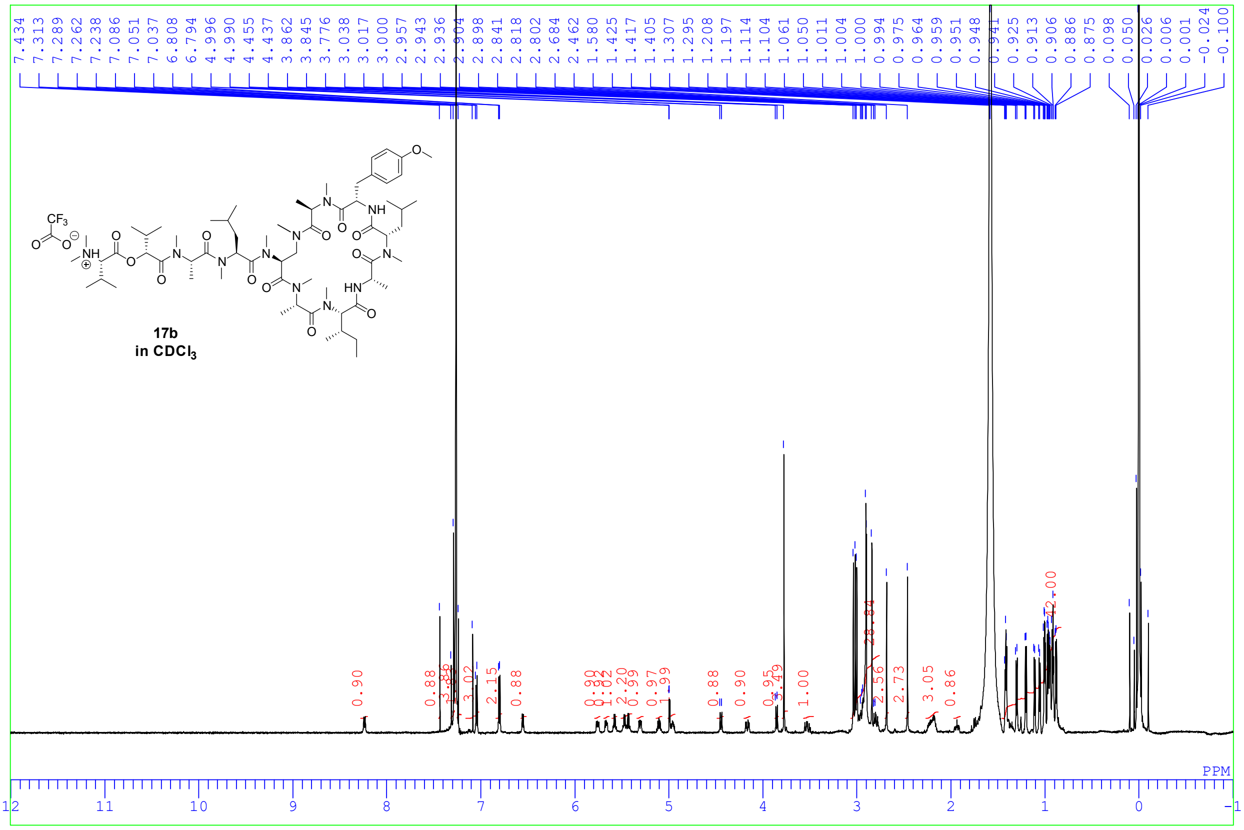

17b
in $CDCl_3$
